# Stathmin 1 regulates mitophagy and non-proliferative cellular function in hematopoietic stem and progenitor cells

**DOI:** 10.1016/j.isci.2026.117458

**Published:** 2026-10-14

**Authors:** Luana Chiquetto, Meg Schuetz, Qian Dong, Mia Warmka, Liana Valin, Ashley Jones, Patrick Hunt, Copper Petermeier, Jie Wang, Nate Roundy, Zev J. Greenberg, Wei Yang, Christine R. Zhang, Grant A. Challen, Cliff J. Luke, Robert A.J. Signer, Wandy L. Beatty, Stephen Sykes, Weikai Li, David J. Kast, Laura G. Schuettpelz

**Affiliations:** 1 Department of Pediatrics, Washington University School of Medicine, St. Louis, MO 63110, USA; 2 Department of Cell Biology and Physiology, Washington University School of Medicine, St. Louis, MO 63110, USA; 3 Department of Genetics, Washington University School of Medicine, St. Louis, MO 63110, USA; 4 Department of Medicine, Washington University School of Medicine, St. Louis, MO 63110, USA; 5 Department of Medicine, Sanford Stem Cell Institute, Moores Cancer Center, University of California, San Diego, La Jolla, CA 92093, USA; 6 Department of Molecular Microbiology, Washington University School of Medicine, St. Louis, MO 63110, USA; 7 Department of Biochemistry and Molecular Biophysics, Washington University School of Medicine, St. Louis, MO 63110, USA

**Keywords:** stathmin, hematopoietic stem cells, metabolism, autophagy, mitophagy, microtubules

## Abstract

Stathmin 1 is a cytoplasmic phosphoprotein that regulates microtubule dynamics. Stathmin 1 expression is a feature of proliferating cells, and it is overexpressed in many cancers, including leukemias. In contrast to its roles in proliferating cells, considerably less is known of stathmin 1 in quiescent cells. Importantly, stathmin 1 is highly expressed in hematopoietic stem and progenitor cells (HSPCs), and herein we found that stathmin 1 loss is associated with altered microtubule architecture and markedly impaired HSPC function. Transcriptomic studies suggested metabolic differences in *Stmn1*^−/−^ HSPCs, and further mechanistic studies revealed defective mitochondrial structure and function. Microtubules facilitate autophagosome formation and mitophagy, and indeed we found that this critical mitochondrial quality control process is impaired in *Stmn1*^−/−^ HSPCs. Finally, stimulation of autophagy improved *Stmn1*^−/−^ HSPC colony-forming ability. Together, these data identify a non-proliferative role for stathmin 1 in the regulation of HSPC mitochondrial quality control and cellular function.

## Highlights

• Loss of stathmin 1 markedly impairs hematopoietic stem and progenitor cell (HSPC) function
• Loss of stathmin 1 alters associations between microtubules and mitochondria in HSPCs
• Loss of stathmin 1 impairs HSPC mitophagy and mitochondrial function

## Graphical abstract

**Figure:**
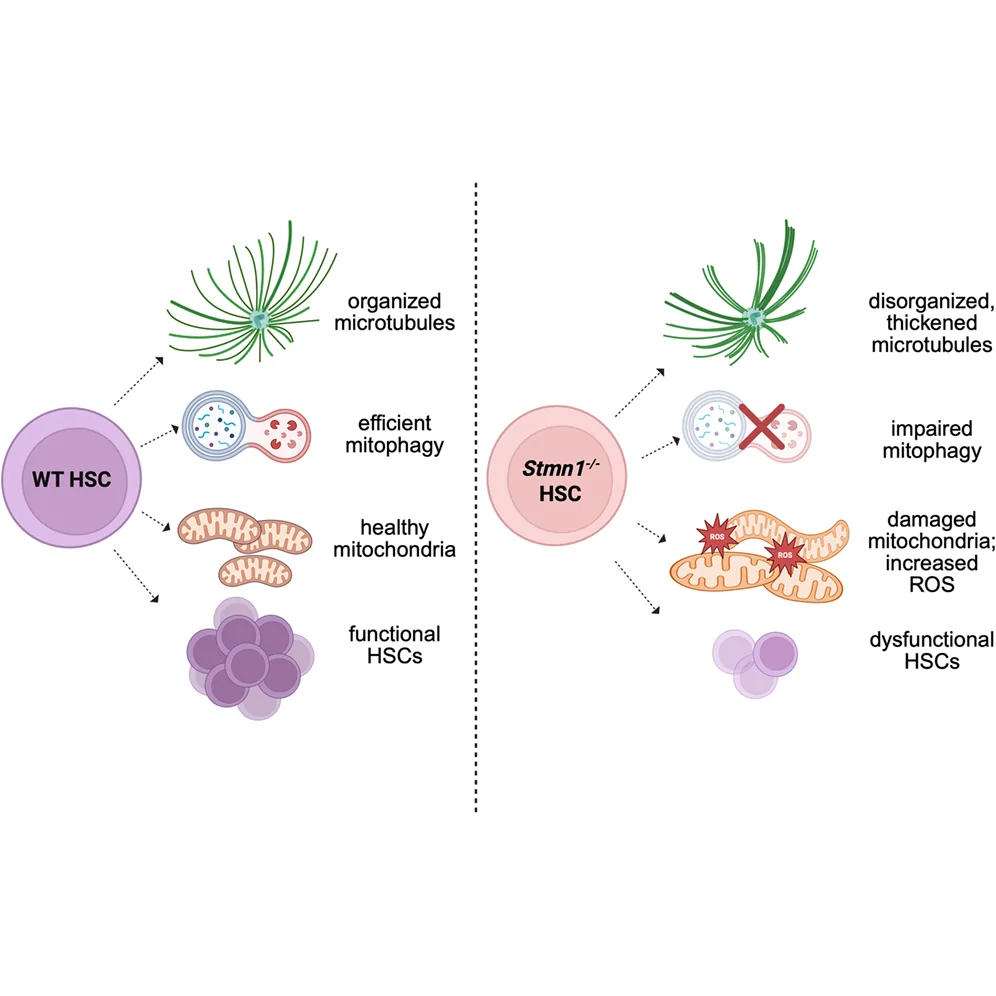

## Teaser

Molecular biology; Cell biology; Stem cells research

## Introduction

Stathmin 1 is a cytoplasmic phosphoprotein that regulates microtubule dynamics via directly promoting microtubule (MT) catastrophe or by regulating microtubule assembly through the sequestration of alpha/beta tubulin heterodimers.^1^^,^^2^^,^^3^ Consequently, stathmin 1 has been implicated in a myriad of MT-dependent processes, including vesicle trafficking, cell spreading, polarization, and cell cycle regulation. Also known as oncoprotein 18 (OP18) or leukemia-associated phosphoprotein p18 (LAP18), stathmin 1 is highly expressed in a wide variety of tumors, including myeloid and lymphoid leukemias, and its downregulation can promote apoptosis and diminish cell proliferation and migration.^4^^,^^5^^,^^6^^,^^7^

In the normal hematopoietic system, stathmin 1 is highly expressed in hematopoietic stem and progenitor cells (HSPCs), and its expression declines with cellular differentiation.^8^ Several studies have implicated stathmin 1 in the regulation of hematopoietic lineage maturation.^9^ For example, stathmin 1 expression is important for normal erythroid differentiation,^10^ and inhibition of stathmin 1 in erythroleukemic cells inhibits their differentiation in response to hemin. In addition, downregulation of stathmin 1 expression is important for normal megakaryocyte maturation and platelet production.^11^ A recent study^12^ showed that *Stmn1*^−/−^ mice have reduced erythrocyte progenitors and a mild, progressive macrocytic anemia that persists throughout life. Megakaryocytes (MKs) and platelets are also altered in the *Stmn1*^−/−^ mice. Early on (age 3 weeks), these mice have reduced megakaryocytes in the marrow and spleen, with reduced circulating platelet counts. As they age, however, platelet counts increase above normal controls, and the bone marrow MKs of aged *Stmn1*^−/−^ mice display enlarged size and ploidy compared to their normal counterparts.^12^

While prior studies have established a role for stathmin 1 in normal erythro-megakaryopoiesis, its role in hematopoiesis remains unclear. Specifically, the role of stathmin 1 in regulating HSPC function is not known. Further, stathmin 1 has largely been studied in proliferative cells, where it is known to regulate cell cycle progression and division,^13^ and its role in comparatively quiescent cells such as HSCs is not well understood. Given the high expression of stathmin 1 in HSCs, as well as the importance of microtubule dynamics to HSPC differentiation,^14^ we hypothesized that loss of stathmin 1 may impact normal hematopoietic stem cell (HSC) and progenitor function.

Indeed, we found that while *Stmn1*^−/−^ mice have largely normal numbers of immunophenotypic HSPCs, their function is markedly impaired in competitive transplants. Further, *Stmn1*^−/−^ mice have delayed hematopoietic recovery from the chemotherapeutic agent 5-fluorouracil (5-FU). Consistent with its role as a regulator of microtubules, *Stmn1*^−/−^ HSPCs have altered microtubule networks. Transcriptome analysis of *Stmn1*^−/−^ HSCs suggested mitochondrial metabolism defects, and subsequent metabolic studies confirmed that *Stmn1*^−/−^ HSPCs have impaired mitochondrial respiratory capacity and increased reactive oxygen species (ROS) production. Further, *Stmn1*^−/−^ HSPCs have abnormal mitochondrial morphology, as revealed by transmission electron microscopy (TEM). Microtubule-mitochondria associations facilitate mitophagy, an essential process for regulating of mitochondrial homeostasis, and indeed this process is impaired in the absence of stathmin 1. Together, these data identify a role for stathmin 1 in the regulation of microtubules in a largely quiescent cell population (HSPCs) to support mitochondrial quality control and cellular function.

## Results

### Stmn1^−/−^ HSPCs have an altered microtubule network

To assess the role of stathmin 1 in HSCs, we first visualized stathmin 1 protein expression and localization by confocal microscopy. Consistent with previous reports of high RNA expression in HSCs,^8^ we detected stathmin 1 protein in sorted HSCs (lineage-c-Kit+ Sca-1+ CD150+ CD48- cells) from young adult (8–10 weeks) wild-type (WT) mice (Figure S1A). Further, in accordance with its known role as a microtubule destabilizer,^2^ stathmin 1 expression in HSCs largely co-localizes with tubulin (Figure S1B). To assess the effects of stathmin 1 loss, we next examined the microtubule network in sorted c-Kit+ HSPCs from *Stmn1*^−/−^ mice and WT controls using a methanol-fixation approach to better visualize the microtubule network. Confocal imaging revealed several differences between the microtubules in *Stmn1*^−/−^ HSPCs compared to WT. Consistent with prior reports, microtubules are distributed in a polar fashion in the WT HSPCs, with fine microtubule networks emanating from the microtubule-organizing center (MTOC) (Figure 1A, left images).^14^^,^^15^ In contrast, *Stmn1*^−/−^ HSPCs display thickened microtubules (Figure 1A, right images). In addition, tubulin levels are reduced in *Stmn1*^−/−^ HSPCs, and *Stmn1*^−/−^ HSPCs are significantly smaller than WT HSPCs (Figures 1B–1D). Thus, stathmin 1 is expressed in HSPCs, and the microtubule architecture is altered in its absence.

**Figure 1:**
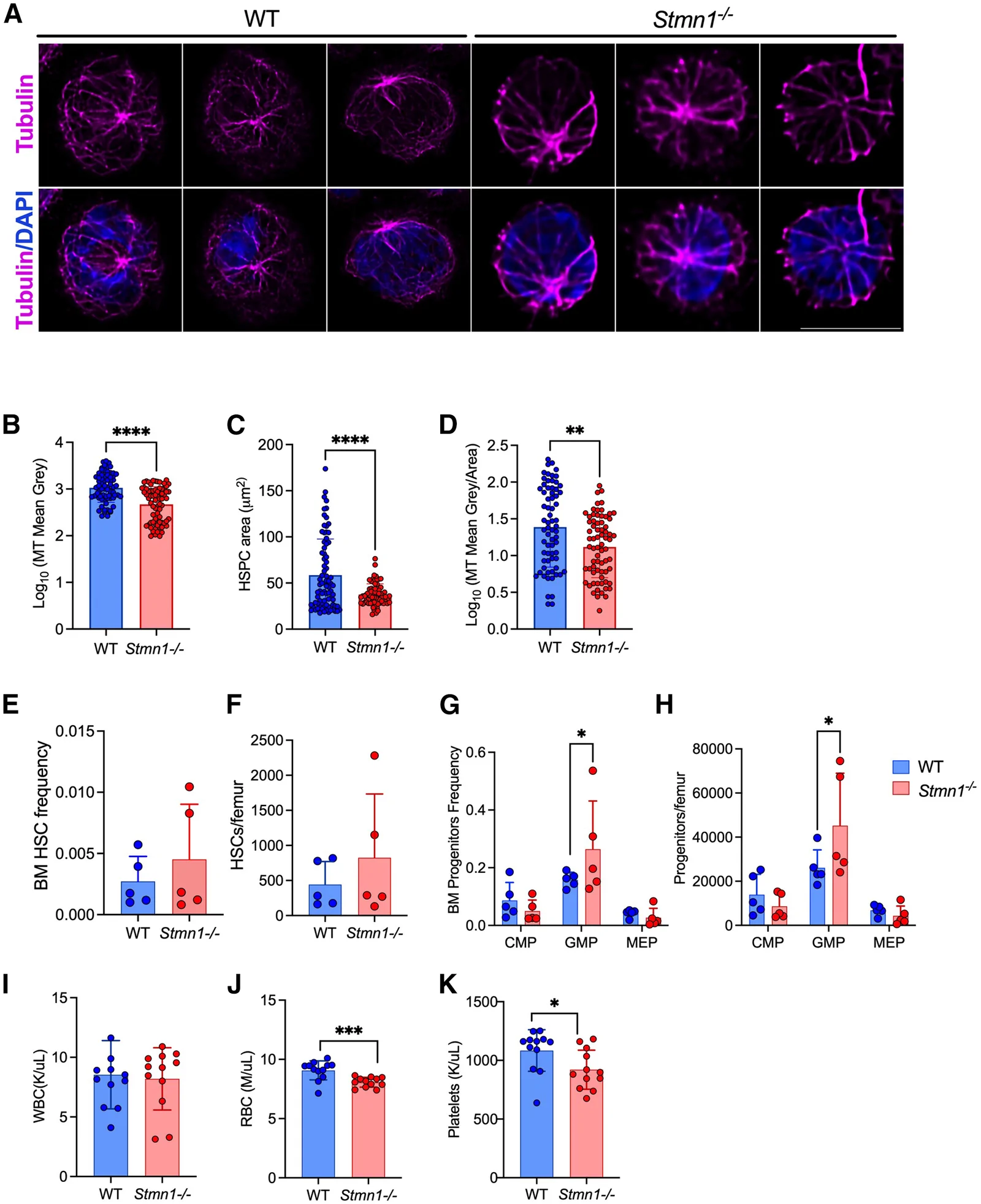
*Stmn1*^*-/-*^ HSPCs have an altered microtubule network (A) Representative images of the microtubule network (see STAR Methods) in HSPCs (c-Kit+ cells) from WT and *Stmn1*^−/−^ mice. Microtubules were stained with alpha-beta tubulin (magenta) and DAPI (blue). (B–D) Quantification of tubulin intensity (B), HSPC area (C), and the ratio of tubulin intensity to area (D). Data represent two biological replicates per group across two independent experiments per group with 72–82 cells. (E–H) Shown are bone marrow HSC frequency (E) and absolute numbers (F) from young adult (8–10-week old) WT and *Stmn1*^−/−^ mice, as well as myeloid progenitor frequencies (G) and absolute numbers (H). See Figure S2A for HSC and progenitor gating strategy. Data represent five biological replicates over three independent experiments. (I–K) Peripheral blood counts from young adult *Stmn1*^−/−^ mice show equivalent numbers of WBCs (I) and modest decreases in RBCs (J) and platelets (K) compared to WT. Data represent 12 mice per group over three independent experiments. Error bars represent mean ± SD. ∗*p* < 0.05, ∗∗∗*p* < 0.001, ∗∗∗∗*p* < 0.001 by unpaired Student’s *t*-test (E–K), Mann-Whitney (C), or Welch’s *t* test (B and D). Scale bars in all representative images represent 5 μm.

### Stmn1^−/−^ HSPCs have impaired function

To further address the specific role of stathmin 1 in HSPCs, we assessed HSPC numbers and function in *Stmn1*^−/−^ mice. Young adult (8–10 weeks) *Stmn1*^−/−^ mice have no significant differences in the frequency or absolute numbers of HSCs in the bone marrow compared to WT littermates (Figures 1E and 1F; Figure S2). However, there is a modest increase in granulocyte-monocyte progenitors (GMPs) (Figures 1G and 1H), indicating a potential role of stathmin 1 in the differentiation of these progenitors. Peripheral blood analysis showed, in accordance with prior reports, that *Stmn1*^−/−^ mice have mild anemia and thrombocytopenia (Figures 1I–1K).^11^^,^^12^

Next, we assessed HSC function using competitive transplants. Whole bone marrow (WBM) cells from *Stmn1*^−/−^ mice or their WT littermates (CD45.2) were mixed 1:1 with WBM from WT competitor (CD45.1), transplanted into lethally irradiated WT recipients (CD45.1/45.2), and peripheral blood chimerism was monitored over time (Figure 2A). Despite having normal HSC numbers (Figures 1E and 1F), the loss of stathmin 1 led to a marked reduction in multilineage repopulating activity, with approximately 80% decrease in peripheral blood chimerism of *Stmn1*^−/−^ cells compared to WT (Figures 2B and 2C). Bone marrow chimerism of *Stmn1*^−/−^ cells (both total WBCs and HSCs) was similarly reduced at 24 weeks post-transplant (Figures 2D and 2E). To further test self-renewal, bone marrow from primary recipients was transplanted into secondary recipients after 24 weeks. Unlike WT bone marrow, *Stmn1*^−/−^ bone marrow did not engraft upon secondary transplantation (Figures S3A and S3B). Importantly, bone marrow homing was not grossly different between WT and *Stmn1*^−/−^ c-Kit+ HSPCs (Figure 2F), and similar engraftment results were obtained when donor cells were transplanted intra-tibially (Figures S3C–S3G), suggesting that the functional defect was not due to impaired homing.

**Figure 2:**
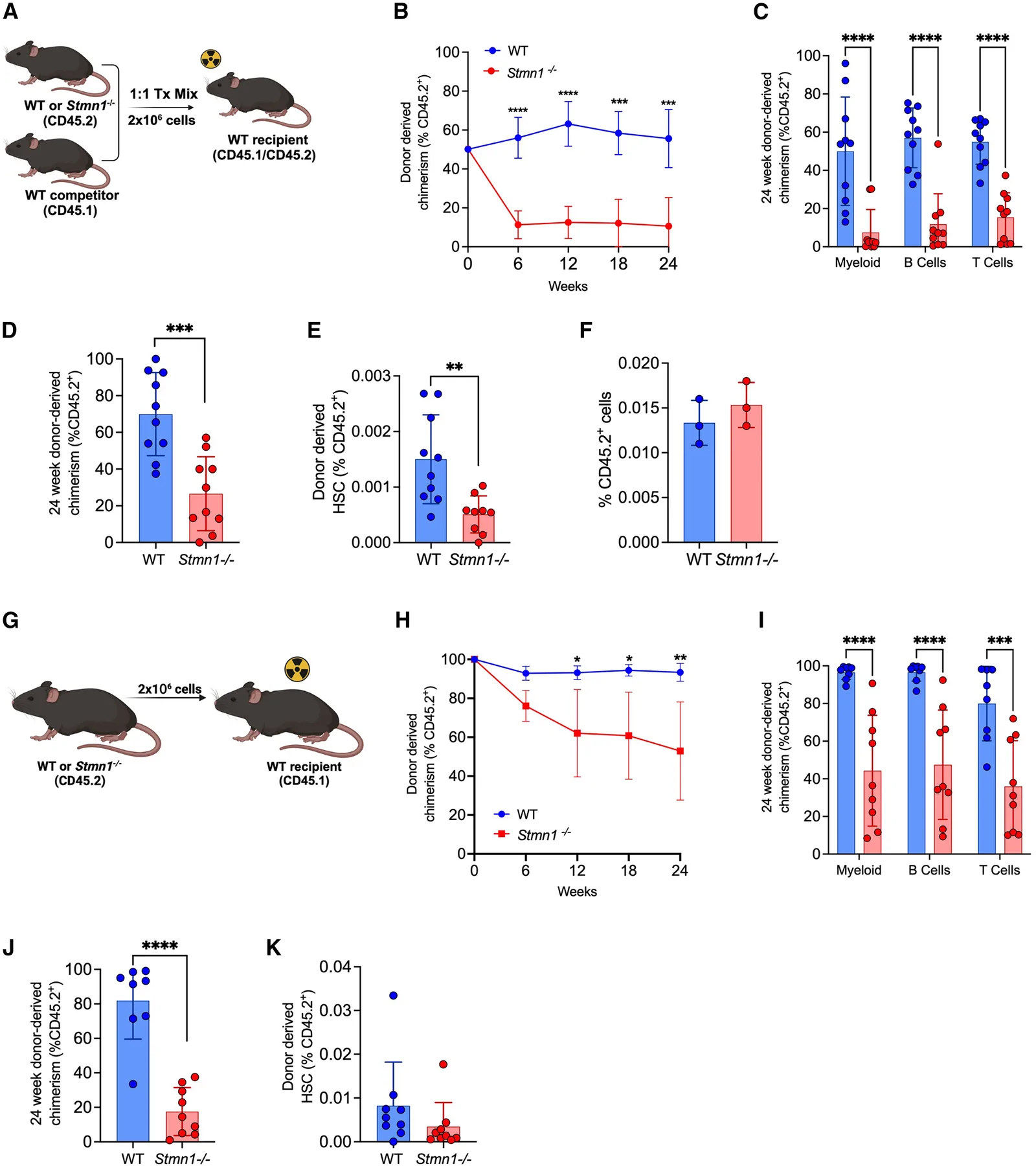
*Stmn1*^−/−^ HSCs have impaired repopulating activity in transplantation assays (A–E) Outline of whole bone marrow (WBM) competitive transplants. WT or *Stmn1*^−/−^ WBM cells (CD45.2) were transplanted in a 1:1 ratio with WT competitor (CD45.1) WBM into irradiated WT recipients (CD45.1/CD45.2). Chimerism was assessed by peripheral blood (PB) flow cytometry analysis every 6 weeks (B). PB engraftment of individual lineages at 24 weeks post-transplant is shown in (C), total bone marrow chimerism (D), and HSC chimerism at 24 weeks post-transplant is shown in (E). Data represent 20 recipients per group from two independent transplantation experiments. (F) Homing was assessed by transferring LSK cells (Lin- Sca-1+ c-Kit+ cells) from WT or *Stmn1*^−/−^ (CD45.2) mice into lethally irradiated CD45.1 WT mice and enumerating CD45.2 cells in the bone marrow 18 h later. (G–K) Outline of non-competitive WBM transplants. WBM cells from WT or *Stmn1*^−/−^ mice (CD45.2) were transplanted without competitor cells into irradiated WT (CD45.1) recipients. Chimerism was assessed by PB flow cytometry analysis every 6 weeks (H). PB engraftment of individual lineages at 24 weeks post-transplant is shown in (I), total bone marrow chimerism (J), and HSC chimerism at 24 weeks post-transplant is shown in (K). Data represent 16–18 recipients per group from two independent transplantation experiments. Error bars represent mean ± SD. ∗*p* < 0.05, ∗∗*p* < 0.01, ∗∗∗*p* < 0.001, and ∗∗∗∗*p* < 0.0001 by unpaired Student’s *t*test or two-way ANOVA.

In addition to competitive transplants, we performed non-competitive transplants in which 2 × 10^6^ WBM cells from WT or *Stmn1*^−/−^ mice were transplanted into lethally irradiated WT recipients, and peripheral blood chimerism was followed over time (Figure 2G). As with the competitive transplants, *Stmn1*^−/−^ cells showed markedly impaired trilineage engraftment (Figures 2H and 2I). *Stmn1*^−/−^ cell bone marrow chimerism (total WBCs and HSCs) was also reduced compared to WT at 24 weeks post-transplant (Figures 2J and 2K).

To further assess HSPC function in a non-transplant context, we treated *Stmn1*^−/−^ and WT littermate mice with a single dose of the chemotherapeutic drug 5-FU or vehicle and measured peripheral blood recovery every 3 days over 30 days. *Stmn1*^−/−^ mice had delayed recovery of leukocytes, erythrocytes, and platelets compared to WT (Figures 3A–3D), consistent with a prior report.^12^ In addition, we sorted individual HSCs from *Stmn1*^−/−^ and WT mice into MethoCult and found that fewer *Stmn1*^−/−^ HSCs formed colonies after 7 days of culture (Figure 3E). We also plated WBM cells and found that *Stmn1*^−/−^ cells formed fewer total colonies than WT and were not capable of serial replating (Figure 3F). Together, these data show that stathmin 1 is necessary for normal HSPC function, including both short-term (progenitor) and long-term (HSC) functions.

**Figure 3:**
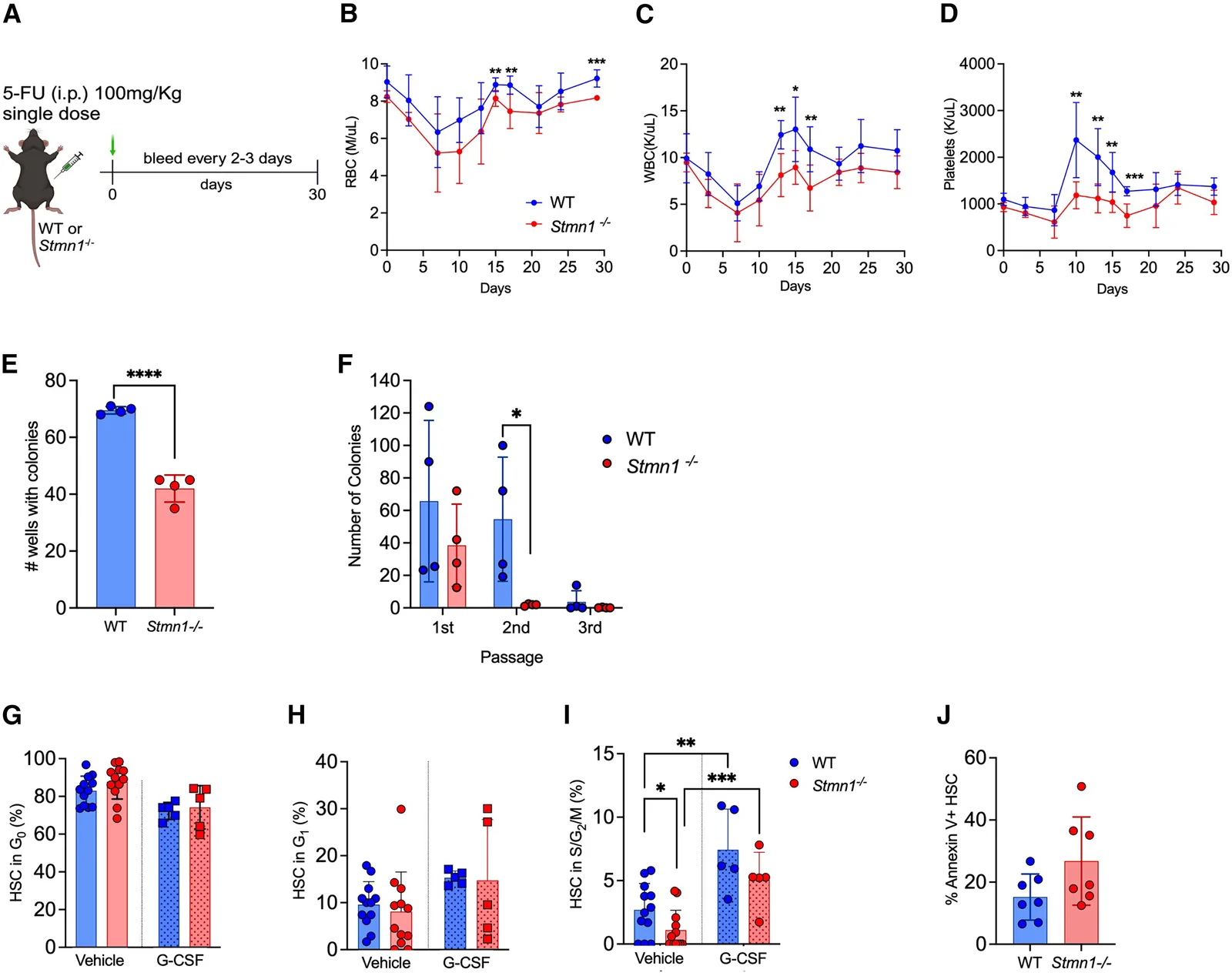
*Stmn1*^*-/-*^ HSPCs have impaired function (A–D) WT or *Stmn1*^−/−^ mice were treated with one single dose of 5-fluorouracil (5-FU, 100 mg/kg). PB was collected to analyze RBCs (B), WBCs (C), and platelets (D) every three days for 30 days. *n* = 7 mice per group from two independent experiments. (E) HSCs were sorted individually into MethoCult 3434 in 96-well plates, and colonies were scored after 7 days. Shown are the total numbers of wells with colonies. (F) *Stmn1*^−/−^ and WT WBM cells were plated in MethoCult 3434 (1 × 10^4^ cells per well), and colonies were scored after 7 days. Cells were then replated (5 × 10^3^ cells/well) and scored every 7 days until no colonies were formed. Data represent four biological replicates over four independent experiments. (G–I) Cell cycling was measured by flow cytometry using Ki-67 and DAPI in WT and *Stmn1*^−/−^ HSCs at baseline (vehicle treatment) and following two doses of G-CSF over 24 h. Shown are the percentages of HSCs in G_0_ (G), G_1_ (H), and S/G_2_/M (I). Data represents 5 (G-CSF treated) and 12 (PBS treated) biological replicates per group over eight independent experiments. See Figure S2B for representative flow plots. (J) Shown are the percent of WT and *Stmn1*^−/−^ HSCs that are annexin V+ at baseline by flow cytometry. Data represent seven biological replicates over six independent experiments. Error bars represent mean ± SD. ∗*p* < 0.05; ∗∗*p* < 0.01; ∗∗∗*p* < 0.001; ∗∗∗∗*p* < 0.0001 by an unpaired Student’s *t* test or two-way ANOVA.

Given the importance of microtubules to cell cycling and the significance of cycling to HSC function,^16^ we next assessed the cell-cycle status of *Stmn1*^−/−^ vs. WT HSCs. At baseline, *Stmn1*^−/−^ HSCs have fewer cells in S/G_2_/M phase (Figures 3G–3I). However, *Stmn1*^−/−^ HSCs can be stimulated to cycle in response to stimulation with the cytokine granulocyte colony-stimulating factor (G-CSF) or the toll-like receptor 2 (TLR2) agonist, PAM_2_CSK_4_, to a similar extent to WT cells (Figures 3G–3I; Figure S1C), suggesting grossly intact stress-induced proliferative capacity. In addition, *Stmn1*^−/−^ HSCs also have slightly increased, albeit not significant, apoptosis at baseline compared to WT as indicated by a trend toward increased annexin V staining (Figure 3J), suggesting that alterations in cell survival may contribute to loss of HSC function in the absence of stathmin 1.

### Stmn1^−/−^ HSPCs have decreased mitochondrial function

To identify mechanisms underlying the loss of HSC function in the absence of stathmin 1, we performed bulk RNA sequencing on sorted HSCs (lineage-c-Kit+ Sca-1+ CD150+ CD48- cells) from *Stmn1*^−/−^ and WT mice. We identified 212 differentially expressed genes between the groups (Table S2). Gene set enrichment analysis (GSEA) of the transcriptome data suggested alterations in metabolism in the *Stmn1*^−/−^ HSCs, particularly indicating altered mitochondrial metabolism with enrichment for Myc targets, mTORC1 signaling, and elevated oxidative phosphorylation (OXPHOS) (Figures 4A and 4B). While Myc and mTOR are important for a myriad of different signaling pathways, they also play a role in regulating genes important for mitochondrial metabolism and enhanced OXPHOS in HSCs.^17^^,^^18^ Genes associated with OXPHOS are highlighted in the volcano plot and heatmap in Figures 4C and 4D (see Table S2 for gene list).

**Figure 4:**
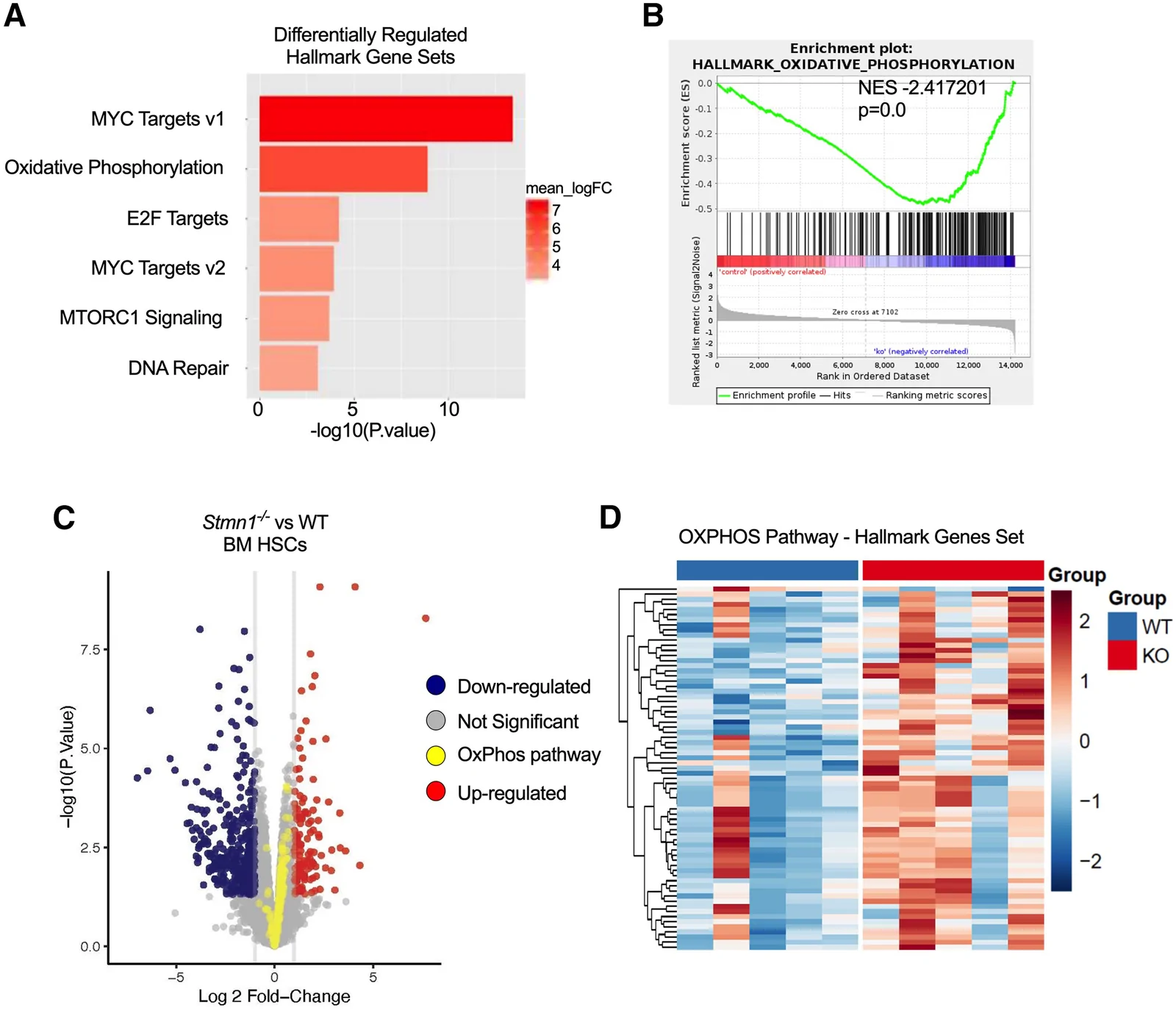
RNA-sequencing identifies altered expression of genes associated with mitochondrial metabolism in *Stmn1*^−/−^ HSCs Bulk RNA sequencing was performed on sorted HSCs (lineage-c-Kit+ Sca-1+ CD150+ CD48- cells) from *Stmn1*^−/−^ and WT mice. (A) Shown are the differentially expressed hallmark gene sets comparing *Stmn1*^−/−^ to WT HSCs. (B) Enrichment plot showing significant differences in oxidative phosphorylation-related genes. (C) Volcano plot of differentially expressed genes comparing *Stmn1*^−/−^ to WT HSCs. Oxidative phosphorylation pathway genes are highlighted in yellow. (D) Oxidative phosphorylation pathway top 71 contributing genes heatmap, *p* < 0.05 (row *Z* score). See Table S2 for gene list. Data represent five biological replicates per group.

To validate the transcriptome data suggesting alterations in mitochondrial metabolism, we next assessed mitochondrial function in the *Stmn1*^−/−^ and WT HSPCs (c-Kit+ cells) using the Seahorse analyzer. We found that while glycolytic capacity is similar between *Stmn1*^−/−^ and WT HSPCs (Figure S4A), mitochondrial respiratory capacity is significantly reduced in the absence of stathmin 1, with *Stmn1*^−/−^ cells showing decreased basal and maximal mitochondrial respiration and reduced spare respiratory capacity (Figures 5A and 5B). We next assessed mitochondrial ROS production and mitochondrial membrane potential using the flow-based MitoSOX and TMRM (tetramethylrhodamine methyl ester) assays and found increased ROS in *Stmn1*^−/−^ HSCs compared to WT (Figures 5C and 5D; Figures S4B and S4C). Of note, elevated ROS is associated with damaged or impaired mitochondria that lack effective mitochondrial quality control mechanisms and/or ROS control measures,^19^ and elevated ROS can be detrimental to HSC function.^17^ These data suggest that the elevated transcript levels of genes associated with OXPHOS are likely a compensatory measure in the face of impaired mitochondrial function.

**Figure 5:**
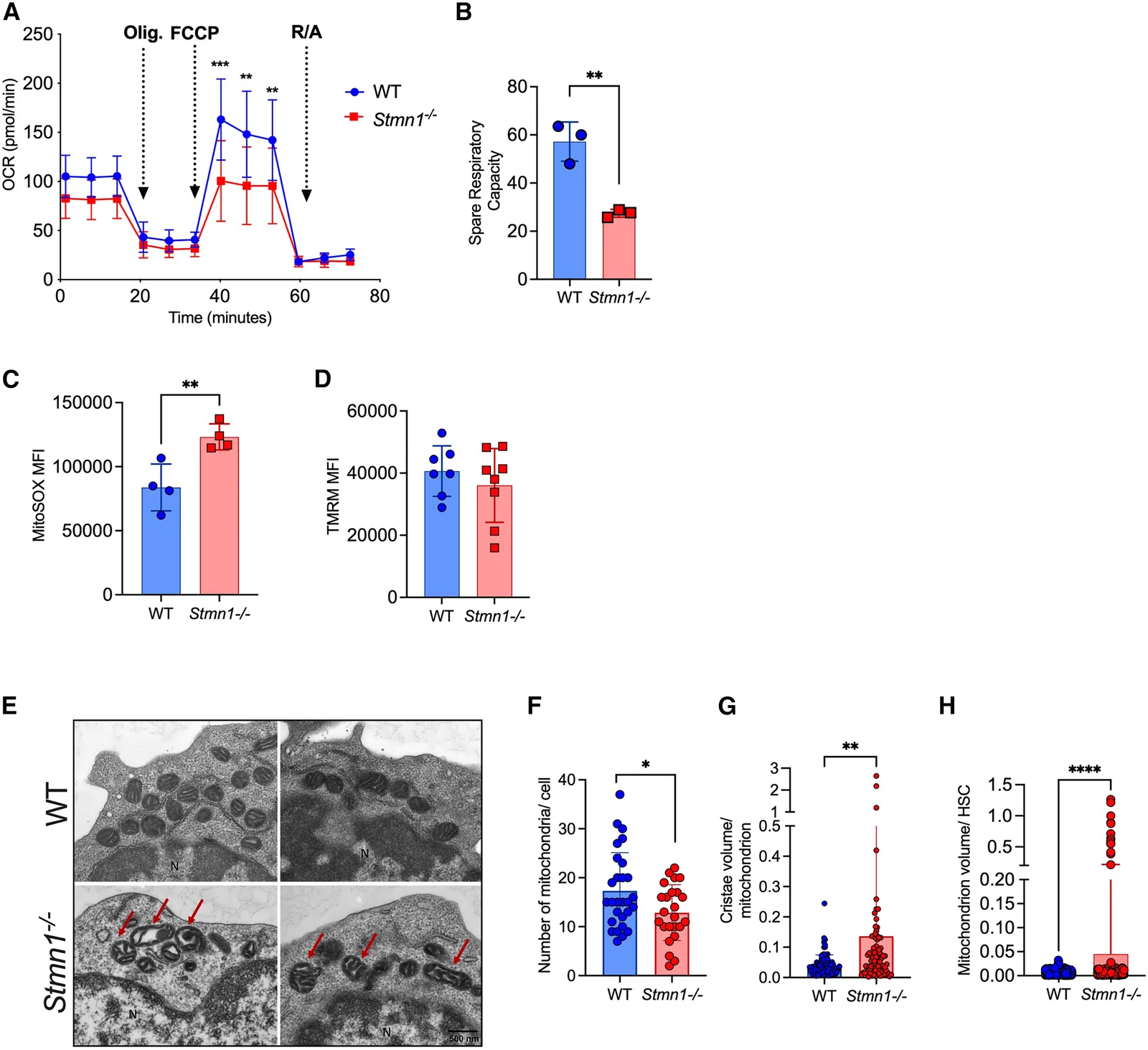
*Stmn1*^*-/-*^ HSPCs have decreased mitochondrial function (A and B) Mitochondrial function (oxygen consumption rate, OCR) was measured on c-Kit+ bone marrow HSPCs from WT and *Stmn1*^−/−^ mice using the Seahorse analyzer. *Stmn1*^−/−^ HSPCs have impaired maximal respiratory capacity (shown following carbonyl cyanide p-trifluoromethoxyphenylhydrazone addition) compared to WT HSPCs (A) and reduced spare respiratory capacity (B), difference between maximal respiratory capacity and basal respiration prior to oligomycin addition. Data are representative of four biological replicates for each group over three independent experiments. (C and D) Shown are HSC mitochondrial superoxide production measured using MitoSOX red (C) and mitochondrial membrane potential measured using TMRM (tetramethylrhodamine methyl ester) (D) by flow cytometry. MFI, mean fluorescence intensity. MitoSOX data represent four biological replicates over four independent experiments. For TMRM data represents eight biological replicates over four independent experiments. (E–H) Representative transmission electron microscopy micrographs (25,000× magnification) of WT and *Stmn1*^−/−^ HSCs showing abnormal mitochondria morphology highlighted by red arrows. *Stmn1*^−/−^ HSCs have reduced mitochondria per cell (F), increased cristae volume per mitochondria (G), and increased mitochondria volume per HSC (H). Data represent two biological replicates per group over the combination of two independent experiments. For (F), 24–28 cells; for (G), 88–94 mitochondria; and for (H), 312–484 mitochondria per cell. Error bars represent mean ± SD. ∗*p* < 0.05, ∗∗*p* < 0.01, ∗∗∗*p* < 0.001 by unpaired Student’s *t* test or (for G and H) Mann-Whitney.

Next, to examine mitochondrial morphology, we performed TEM on isolated HSCs (lineage-c-Kit+ Sca-1+ CD150+ CD48-cells) from WT and *Stmn1*^−/−^ mice. As shown in representative micrographs in Figure 5E, the mitochondria of *Stmn1*^−/−^ HSCs have markedly abnormal morphology. While overall mitochondria numbers per cell are decreased in *Stmn1*^−/−^ HSCs (Figure 5F), they have increased cristae volume and mitochondrial volume compared to WT (Figures 5G and 5H). To determine whether these mitochondrial abnormalities are unique to HSCs, we also sorted GMP (lineage-c-Kit+ Sca-1-CD150- CD105− CD16/32+ cells) and Gr-1+ granulocytes from *Stmn1*^−/−^ and WT mice for assessment of stathmin 1 expression by confocal immunofluorescence and mitochondrial morphology by TEM. Prior studies have suggested lower stathmin 1 expression in mature effector cells compared to HSCs,^8^^,^^9^ and indeed we detected lower (albeit not undetectable) levels of stathmin 1 protein in the GMPs and Gr-1+ cells compared to HSCs (Figures S5A and S5B). As shown in Figures S6A–S6D, the mitochondria in the *Stmn1*^−/−^ GMP population also have fewer mitochondria per cell, with morphologic abnormalities and reduced electron density, increased mitochondrial size and increased cristae volume. Similarly, the mitochondria in the *Stmn1*^−/−^ Gr1+ granulocytes (Figures S6E–S6H) have enlarged mitochondria with increased cristae volume. Importantly, however, the microtubules in Ficoll-separated granulocytes from *Stmn1*^−/−^ mice are not significantly different from WT, with no differences in tubulin as determined by mean gray value (Figures S6I–S6K), although the *Stmn1*^−/−^ granulocytes are significantly smaller than WT (Figure S6L). These data suggest that the loss of stathmin 1 primarily affects the microtubule architecture of HSPCs, although some mitochondrial morphologic abnormalities persist in mature granulocytes.

### HSPC mitophagy is impaired in the absence of stathmin 1

Together, our data suggest that stathmin 1 is necessary to maintain normal microtubule architecture, as well as mitochondrial and cellular function, in HSPCs. We considered that mitophagy, a specialized form of autophagy and a major regulator of mitochondrial quality, may be altered in *Stmn1*^−/−^ HSCs. High autophagic flux, and, in particular, the ability to clear damaged mitochondria by mitophagy, are key factors in maintaining HSC function.^20^ While the majority of autophagy-associated genes were not differentially expressed in *Stmn1*^−/−^ HSCs compared to WT (Figures S7A and S7B), microtubules are important for the trafficking of lysosomes needed for the completion of mitophagy. Thus, alterations in microtubule architecture could potentially impair this process and lead to accumulation of dysfunctional mitochondria.^21^^,^^22^^,^^23^

To assess mitophagy, we first used confocal microscopy on sorted HSCs to evaluate the expression of Drp1 (dynamin-related protein 1), a protein involved in mitochondrial fission that promotes mitophagy.^24^^,^^25^ While we did not detect any differences in localization of Drp1 to mitochondria (indicated by staining for the outer mitochondrial membrane protein Tom20), Drp1 levels are decreased in *Stmn1*^−/−^ HSCs compared to WT (Figures 6A–6C). Drp1-mediated fission is often the first step in segregating damaged mitochondria that are subsequently targeted for autophagic degradation by PTEN-induced kinase 1 (PINK1) and Parkin (an E3 ubiquitin ligase).^26^ The latter two accumulate on the outer membrane of damaged mitochondria, and thus their immunofluorescence intensity can reflect the degree of mitophagy. PINK1 showed reduced levels in *Stmn1*^−/−^ HSCs and reduced co-localization with mitochondria (Figures 6D–6F). Parkin, which also attaches to the outer membrane of damaged mitochondria,^27^ co-localizes with mitochondria (Tom20) in *Stmn1*^−/−^ HSCs. However, we detected overall reduced levels of Parkin in *Stmn1*^−/−^ HSCs compared to WT (Figures 6G–6I), suggesting reduced basal mitophagy. Next, we wanted to know if stathmin 1 more profoundly impacted mitophagy under conditions that drive mitochondria damage. To that end, we stained for Parkin in sorted HSCs treated with an established inhibitor of mitochondria OXPHOS, CCCP (carbonyl cyanide *m*-chlorophenyl hydrazone), to induce mitophagy. Compared to untreated baseline, CCCP-treated WT HSCs had an expected increase in Parkin puncta, while *Stmn1*^−/−^ HSCs had a significantly diminished increase in puncta consistent with decreased mitophagy (Figures 6J and 6K). Of note, impaired mitochondrial fission and defective mitophagy can result in enlarged or swollen mitochondria with abnormal cristae due to the accumulation of damaged mitochondria that cannot be segregated and eliminated,^28^^,^^29^^,^^30^ consistent with our TEM imaging results (Figure 5E).

**Figure 6:**
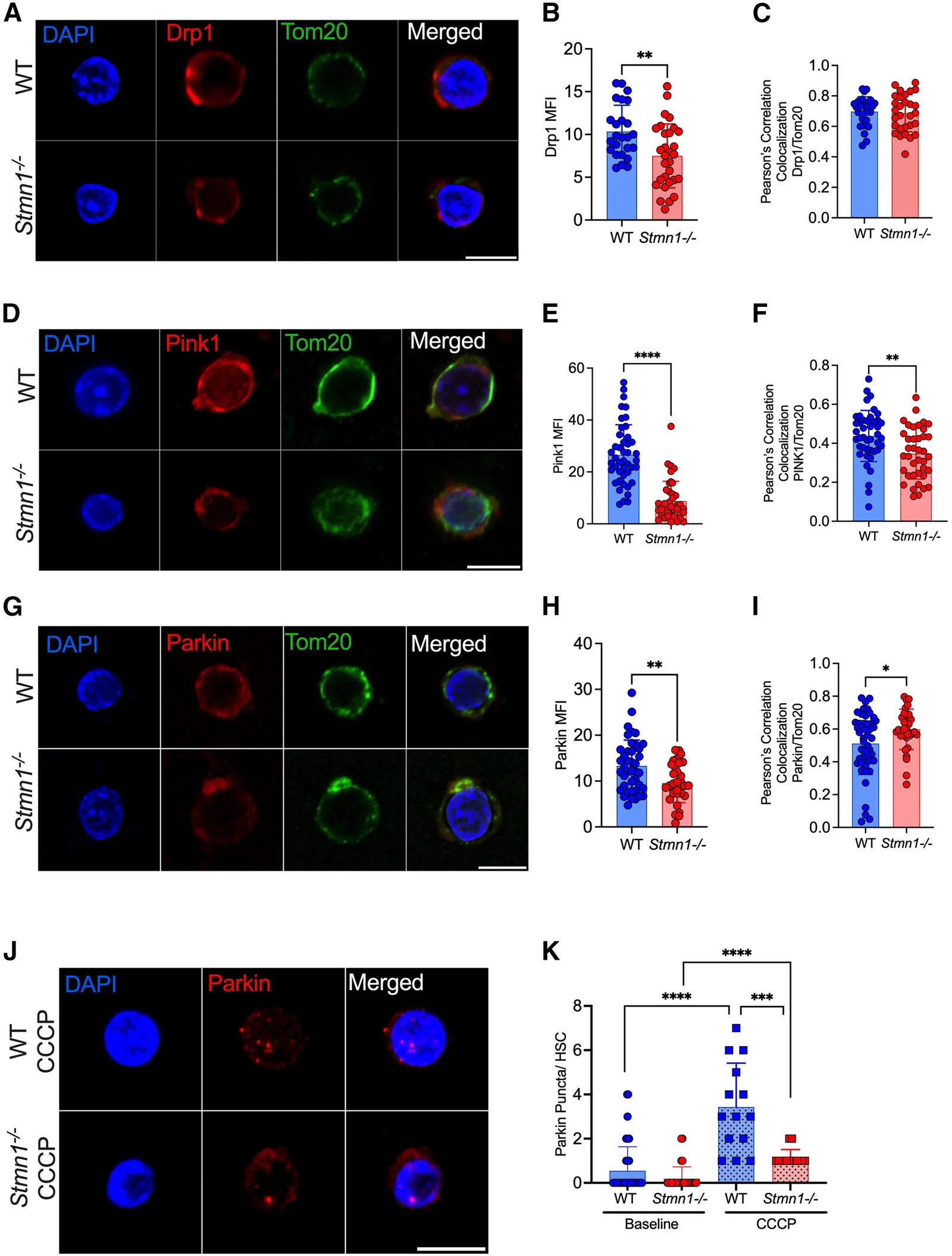
*Stmn1*^−/−^ HSCs have impaired mitophagy (A) Representative confocal images of HSCs (lineage-c-Kit+ Sca-1+ CD150+ CD48-cells) from WT and *Stmn1*^−/−^ mice stained for DAPI, Drp1 and Tom20. (B and C) Quantification of Drp1 mean fluorescence intensity (MFI) and colocalization with the mitochondria marker Tom20. (D) Representative confocal images of HSCs stained for DAPI, PINK1, and Tom20. (E and F) Quantification of PINK1 MFI and colocalization with Tom20. (G) Representative confocal images of HSCs stained for DAPI, Parkin, and Tom20. (H and I) Quantification of Parkin MFI and colocalization with Tom20. (J) Representative confocal images of HSCs stained for Parkin following treatment with CCCP. (K) Quantification of Parkin puncta per HSC at baseline and following CCCP treatment. Data represent the combination of two biological replicates over two independent experiments and 40+ cells per group. Error bars represent mean ± SD. ∗*p* < 0.05, ∗∗*p* < 0.01, ∗∗∗*p* < 0.001, ∗∗∗∗*p* < 0.0001 by unpaired Student’s *t* test. Scale bars in all representative images represent 5 μm.

Following Parkin-mediated ubiquitination, mitochondria are captured by LC3-containing autophagosomes prior to fusion with lysosomes.^31^^,^^32^ LC3 (microtubule-associated protein light chain 3) is a key protein in the autophagy pathway,^33^ where it serves as an anchor for autophagy adapter proteins that bind to ubiquitinylated proteins, and thus its abundance correlates with the degree of autophagy. Therefore, we measured LC3 levels in sorted HSCs both at baseline and following treatment with an inhibitor of lysosomal proteolysis, leupeptin. Autophagic flux is measured by the accumulation of LC3 in the presence of leupeptin.^34^ At baseline, LC3 levels measured by immunofluorescence are similar between WT and *Stmn1*^−/−^ HSCs (Figures 7A–7C). However, following leupeptin treatment, LC3 levels were significantly higher in the WT HSCs compared to *Stmn1*^−/−^ HSCs (Figures 7D and 7E). Thus, autophagic flux is reduced in *Stmn1*^−/−^ HSCs (Figure 7F). Finally, to more definitively show that autophagic clearance of mitochondria was reduced in *Stmn1*^−/−^ cells, we used the pH-sensitive mitochondrial reporter mt-Keima to assess mitochondrial delivery to acidic autolysosome compartments. HSPCs from WT and *Stmn1*^−/−^ mice were transduced with a mt-Keima-expressing lentivirus and analyzed by flow cytometry using dual excitation at 405 nm (indicating neutral/alkaline pH or cytoplasmic localization) and 561 nm (indicating acidic pH or autolysosomal localization).^35^ *Stmn1*^−/−^ HSPCs treated with CCCP showed a decreased mt-Keima 561/405 excitation ratio compared to WT cells, indicating reduced localization of mitochondria to acidic compartments (Figure 7G). These findings suggest that stathmin 1 not only promotes mitochondrial health by promoting mitophagy but is important for autophagy in general.

**Figure 7:**
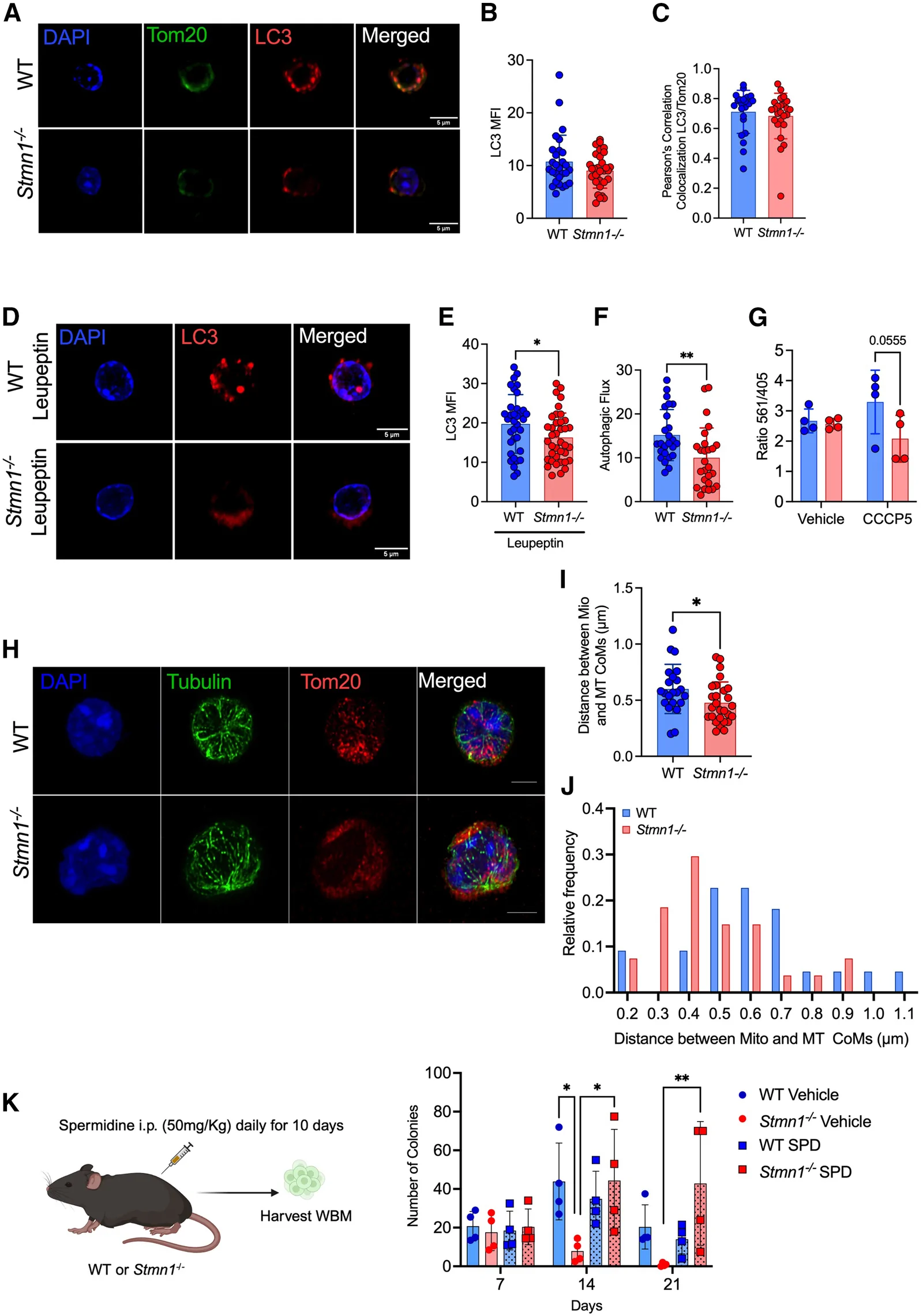
*Stmn1*^−/−^ HSCs have reduced autophagic flux (A) Representative confocal images of HSCs (lineage-c-Kit+ Sca-1+ CD150+ CD48-cells) from WT and Stmn1^−/−^ mice stained for Tom20 and LC3. (B and C) Quantification of LC3 MFI at baseline and co-localization of LC3 and Tom20. (D–F) Autophagy flux was measured by treating sorted HSCs with leupeptin. Shown are representative confocal images of WT and *Stmn1*^−/−^ HSCs stained for LC3 following leupeptin treatment (D), quantification of LC3 MFI in the treated cells (E), and autophagic flux as measured by the difference in LC3 +/− leupeptin (F). Data represent the combination of two biological samples per condition over two independent experiments with 38 cells per group. (G) c-Kit+ cells from WT and *Stmn1*^−/−^ mice were transduced with a mKeima-expressing lentivirus and analyzed by flow cytometry using dual excitation at 405 and 561 nm. Shown are the ratio of acidic (561 nm, lysosomal) to neutral (405 nm, cytoplasmic) mitochondria at baseline (vehicle) and following treatment with the autophagy inducer CCCP (5 μM). Data represent four biological replicates over four independent experiments. (H–J) Representative confocal images of sorted HSPCs (c-Kit+ cells) showing microtubule (α-tubulin) and mitochondria (Tom20) staining. Quantification of the difference between the microtubule mitochondria center-of-mass (CoM, image [I]) and the frequency distribution of these differences (J) is plotted on the right. Data represent two biological replicates over two independent experiments with 22–27 cells per group. (K) WT and *Stmn1*^−/−^ mice were treated with spermidine (50 mg/kg once daily) or vehicle for 10 days. Bone marrow from spermidine-treated and vehicle control mice was plated in MethoCult 3434 (1 × 10^4^ cells per well), and colonies were scored after 7 days. Cells were then replated (5 × 10^3^ cells/well) and scored every 7 days. The data represent three biological replicates per group over three independent experiments. Error bars represent mean ± SD. ∗*p* < 0.05, ∗∗*p* < 0.01 by unpaired Student’s *t* test or two-way ANOVA. Scale bars in all representative images represent 5 μm.

### Microtubule-mitochondria associations are altered in the absence of stathmin

Microtubules not only serve as tracks to support the motility of organelle-carrying motor proteins but their dynamics can directly influence a myriad of organelle activities. For mitochondria, these activities include fission and the clearance of damaged mitochondria by mitophagy. Given the alterations in the microtubule network and the impairment in mitophagy in *Stmn1*^−/−^ HSPCs, we predicted that the co-localization of mitochondria and microtubules may be affected in *Stmn1*^−/−^ HSPCs. We thus quantified the association between microtubules and mitochondria in the presence and absence of stathmin 1 using immunofluorescence. In both glutaraldehyde-fixed WT and *Stmn1*^−/−^ HSPCs, mitochondria showed strong colocalization with microtubules (Figure 7H), indicating that stathmin 1 loss does not influence association of mitochondria with microtubules. However, upon visual inspection of these cells, we found mitochondria were less uniformly distributed in *Stmn1*^−/−^ HSPCs when compared to WT HSPCs. Quantification of the 3D distribution of mitochondria relative to the 3D distribution of microtubules revealed that the center of mass of the mitochondria was significantly closer to the center of mass of microtubules in *Stmn1*^−/−^ HSPCs (Figure 7I) and biased toward separations of 0.3–0.4 μm (Figure 7J). This suggests that stathmin-mediated regulation of microtubule architecture may be important for the trafficking and positioning of mitochondria throughout the HSPCs.

### Autophagy stimulation improves Stmn1^−/−^ HSPC colony formation

Together, our data suggest that the reduced function of *Stmn1*^−/−^ HSPCs may be due, at least in part, to impairment in mitophagy. We therefore reasoned that stimulation of autophagy/mitophagy may improve *Stmn1*^−/−^ HSPC function, and to this end we treated mice with the autophagy inducer spermidine (50 mg/kg daily × 10 days, Figure 7I).^36^ As shown in Figure 7K, WBM from spermidine-treated *Stmn1*^−/−^ mice produced colonies with improved serial replating capacity in MethoCult compared to vehicle-treated controls, suggesting some improvement in HSPC function. Importantly, while spermidine treatment increased LC3 levels in both WT and *Stmn1*^−/−^ HSCs (consistent with autophagy stimulation), the levels in *Stmn1*^−/−^ HSCs did not reach those of WT (Figure S8), supporting the idea that autophagy is impaired in the absence of stathmin 1.

## Discussion

Prior studies of stathmin 1 have established a role for this microtubule-destabilizing protein in highly proliferative cells, where it regulates microtubules and mitotic spindle architecture to promote cell cycling and division. In hematopoiesis, stathmin 1 was previously shown to be important for normal erythropoiesis and megakaryopoiesis,^11^^,^^12^ where relatively high levels are thought to be important in proliferating progenitor populations and downregulation of stathmin 1 expression supports normal cell differentiation. Importantly, these prior studies did not assess the role of stathmin 1 in HSCs, though they did report, consistent with our findings, normal HSPC numbers in *Stmn1*^−/−^ mice.^12^ However, the highest expression of stathmin 1 in the hematopoietic system is in long-term HSCs, which are largely quiescent, and we now show that loss of stathmin 1 leads to severely reduced repopulating activity in transplantation experiments and reduced colony formation of sorted HSCs. These findings suggest a long-term HSC defect, as *Stmn1*^−/−^ bone marrow cells fail to engraft myeloid and lymphoid lineage cells in transplanted recipient mice (Figure 2). Furthermore, *Stmn1*^−/−^ mice have impaired recovery of RBCs, WBCs, and platelets following 5-FU treatment, suggesting that this HSC defect is not simply a context-dependent effect of transplantation.

Given that the *Stmn1*^−/−^ HSCs are capable of stress-induced cycling to the same extent as their WT counterparts (Figures 3G–3I), this loss of function is not likely simply a result of reduced proliferative capacity, but rather due, at least in part, to the effects of stathmin 1 loss on mitochondrial function. Thus, our data expand the role of stathmin 1 beyond the support of cell proliferation, as we now show that it is essential to maintain HSPC mitochondrial health and metabolism in even the most primitive, quiescent HSCs.

HSCs display unique metabolic characteristics compared to differentiated cells. Specifically, they are characterized by high levels of relatively inactive mitochondria, and they rely heavily on glycolysis over OXPHOS for ATP production.^17^^,^^37^^,^^38^ Because of this, they produce low levels of ROS. HSCs are very sensitive to ROS, as any alterations in mitochondrial metabolism that switches from glycolysis to OXPHOS and increased ROS production are associated with HSC differentiation.^17^ Consequently, mutations that compromise mitochondrial function or increase ROS are often associated with a reduction in the HSC pool and loss of HSC function,^17^ and alterations in mitochondrial function are thought to contribute to the decline in HSC function with normal aging.^38^

To maintain reduced OXPHOS and ROS, HSCs rely on high levels of mitophagy to maintain quiescence.^39^ This is even maintained during cell division, where one daughter cell, destined for differentiation, will inherit healthy mitochondria, and the self-renewing daughter cell will inherit mitochondria associated with autophagosomes.^40^^,^^41^ Any reduction in autophagy can persistently elevate ROS, and both are associated with loss of HSC stemness and reduced function.^39^^,^^42^ Despite the importance of tight mitochondrial quality control to HSC function, the mechanisms that maintain it are not well understood. We now show that stathmin 1 is a critical regulator of this process and provide evidence that stathmin 1 influences mitophagy in a quiescent cell population. While a prior study found stathmin 1 to promote mitochondrial integrity in stressed hepatocytes via downregulation of c-Jun N-terminal kinase (JNK),^43^ and a second study showed that stathmin 1 promotes paclitaxel-induced autophagy in osteosarcoma cell lines,^44^ this is the first report of a role for stathmin 1 specifically in supporting mitophagy.

In addition to mitophagy, microtubule dynamics play a crucial role in the positioning of mitochondria, lysosomes, and other organelles within the cell. For example, it was shown that local disassembly and catastrophe of MTs are important for determining destinations of cargoes transported by kinesins in neurons and dendrites.^45^^,^^46^ stathmin 1, specifically, was shown to regulate the localization of the GluA2 subunit of the AMPAR receptor in dendrites.^46^ Thus, stathmin 1 may serve as similar function for the spatially distributing mitochondria. Indeed, we did observe that depletion of stathmin 1 resulted in a mitochondria distribution that accumulated closer to the MTOC than was seen in WT HSPCs (Figure 7I). Interestingly, stathmin 1 has been shown to be important for MTOC polarization in mature T cells,^47^ and loss of MTOC polarization has been shown to impede the localization of mitochondria (as well as other organelles) to the immune synapse.^48^ This repositioning of mitochondria was shown to be independent of microtubule motors, but instead was shown to depend on actin and DRP1,^48^ which localizes more strongly to mitochondria when microtubules are destabilized.^49^ Thus, stathmin 1 could regulate mitochondria localization, fission, and mitophagy through localized instability of microtubules and polarization of the MTOC.

While our data clearly support a role for stathmin 1 in regulating mitochondrial function in HSPCs, it is not clear whether it similarly promotes mitochondrial function in differentiated effector cell populations. Our imaging studies of isolated granulocytes show that mitochondrial morphology remains altered in these cells, suggesting that stathmin 1 may be required for their mitophagy and cellular function. However, microtubules were not grossly altered in *Stmn1*^−/−^ bone marrow granulocytes (Figures S6I–S6K). This could be explained by the reduced dependence of terminally differentiated granulocytes on microtubule turnover, or by compensatory mechanisms during differentiation or selection for daughter cells that normalize the effects of Stmn1 loss. For example, other stathmin family members (Stmn2–4), while normally largely confined to cells of the nervous system,^50^ could potentially compensate for stathmin 1 loss. Further studies are needed to assess the expression of these genes, as well as other microtubule regulators, in hematopoietic cells. Further studies are also needed to elucidate the specific, cell-intrinsic role of stathmin 1 in HSPCs and other lineages in the hematopoietic system. Whether stathmin 1’s role in HSCs is distinct from its role in megakaryocytes or erythrocytes is an open question requiring further study in conditional knockout mice. Further, a contribution of stathmin 1 in the bone marrow niche cells (e.g., abnormal megakaryocytes) to the observed phenotypes in HSPCs cannot be excluded based on our analyses of constitutional knockout mice, and also awaits further study in conditional knockout models.

Finally, the role of stathmin 1 in malignant HSPCs is not clear. Stathmin 1 is highly expressed in both myeloid and lymphoid leukemias, where it has been shown to promote cell growth and survival.^5^^,^^6^^,^^7^^,^^9^ Leukemic cells are metabolically distinct from healthy HSPCs, with a stronger reliance on OXPHOS,^51^ suggesting that elevated levels of stathmin 1 may facilitate leukemogenesis via promotion (at least in part) of mitochondrial activity. While inhibition of stathmin 1 has been shown to induce cell-cycle arrest and apoptosis in leukemia cell lines,^52^ the specific mechanisms by which it promotes leukemic cell growth and survival are not well understood. Further studies are thus needed to determine whether stathmin 1 regulates mitochondrial quality in malignant HSPCs.

### Limitations of the study

While our study clearly indicates a novel role for stathmin 1 in the promotion of mitophagy and mitochondrial quality control in HSPCs, the specific effects of stathmin 1 loss on microtubule dynamics in these cells require further study. Further, the mechanisms by which stathmin 1 influences mitophagy (e.g., organelle and lysosome positioning, autophagosome formation, etc.) await further investigation using live-cell imaging. Finally, we performed the experiments in this study using constitutional knockout mice, which precludes the ability to test cell-intrinsic and cell type-specific effects of stathmin 1 loss. Future studies using conditional knockout mice, as well as overexpression and rescue experiments, will be necessary to fully elucidate the roles of stathmin 1 in HSPCs and other hematopoietic cells.

## Resource availability

### Lead contact

Further information and request for resources and reagents should be directed to and will be fulfilled by the lead contact, Laura Schuettpelz (schuettpelz_l@wustl.edu).

### Materials availability

This study did not generate new unique reagents.

### Data and code availability

• RNA-seq data have been deposited to Gene Expression Omnibus at GEO: GSE328105.
• This study does not generate any new custom code.
• Any additional information required to reanalyze the data reported will be shared by the lead contact upon request.

## Acknowledgments

The authors thank the DCM of Washington University for animal care. This work was supported by funding from The Children’s Discovery Institute of St. Louis Children’s Hospital Foundation (C.J.L. and L.G.S.), the National Heart, Lung, and Blood Institute (R01 HL134896; L.G.S.), the National Institute for General Medical Science (R35 GM158363; D.J.K.), and the Alvin J. Siteman Cancer Center Siteman Investment Program (supported by the 10.13039/100007338Foundation for Barnes-Jewish Hospital, Cancer Frontier Fund).

## Author contributions

L.C. and L.G.S. designed the research and wrote the manuscript; L.C., M.S., Q.D., M.W., L.V., A.J., P.H., C.P., J.W., N.R., Z.J.G., W.Y., and C.R.Z. performed and analyzed the results of the experiments; L.C. and M.S. prepared the figures; G.A.C., C.J.L., R.A.J.S., W.L.B., S.S., W.L., and D.J.K. provided critical resources and supervision of personnel.

## Declaration of interests

The authors declare no competing interests.

## STAR★Methods

### Key resources table

**Table:** 

### Experimental model and study participant details

#### Animal studies

*Stmn1*^−/−^ (B6.129P2-*Stmn1*^*tm1Wed*^/J), C57BL/6J (CD45.2), and C57BL/6 mice (B6.SJL-Ptprc^a^ Pepc^b^/BoyJ) carrying the CD45.1 allele were purchased from the Jackson Laboratory (Bar Harbor, ME). WT littermates were used throughout the study as controls. Young adult (8–10 weeks) sex- and age-matched mice were used for all experiments in accordance with the guidelines of the Washington University Animal Studies Committee, and all experiments conform to the relevant regulatory standards. Animal experiments were approved by the Institutional Animal Care and Use Committee of Washington University in St. Louis (24–0337). Both male and female mice were used in the study, and no obvious differences were noted in the results between the sexes.

### Method details

#### Flow cytometry

Peripheral blood was obtained by retro-orbital sampling, and cell counts determined using an Element HT5 (Heska Corporation, Loveland, CO, USA) automated cell counter. Bone marrow cells were isolated by centrifugation of long bones at 6,000 rpm for 3 min. After lysis of red blood cells, samples were stained (see Table S1 for list of antibodies) and analyzed on NovoCyte flow cytometer (Agilent Technologies, San Diego, CA, USA) or an Aurora flow cytometer (Cytek Biosciences, Freemont, CA, USA). Data was analyzed with FlowJo software (version 10.5.3; TreeStar, Ashland, OR, USA).

#### Data sharing

RNA-seq data are available at GEO under accession number GSE328105.

#### Long-term repopulating assays

Whole bone marrow (WBM) cells from *Stmn1*^−/−^ mice or WT littermates (CD45.2) were transplanted with or without WT (CD45.1) competitor WBM cells (2 × 10^6^ cells total) via retro-orbital injection into lethally irradiated (two doses of 550 cGy spaced 4h apart) WT (CD45.1/CD45.2) recipients. For intra-tibial transplants, a total of 2.5 × 10^5^ cells were transplanted directly into the tibias of lethally irradiated recipients. Peripheral blood chimerism was determined via retro-orbital sampling every 6 weeks.

#### Seahorse Mito Stress and glycolysis stress tests

Seahorse Mito Stress Test and Glycolysis Stress Test kits (Agilent Technologies, Santa Clara, CA, USA) were used to measure oxygen consumption rate (OCR) and extracellular acidification rate (ECAR) using a 96-well Seahorse Bioanalyzer XFe96 following manufacturer’s instructions. c-Kit+ cells were plated at a density of 1 × 10^5^ cells per well into 96 well plates with StemSpan media with thrombopoietin (TPO; 100 ng/mL, PeproTech, Cranbury, NJ, USA), stem cell factor (SCF; 100 ng/mL, PeproTech), IL-3 (10 ng/mL, PeproTech), FLT3L (50 ng/mL, PeproTech), and G-CSF (10 ng/mL, PeproTech) for 18 h at 37C with 5%CO_2_. For Mito Stress Test (OCR), cells were then washed and resuspended in XF base media (RPMI supplemented with Glutamine (200 mM), glucose (2.5 M), and Sodium-pyruvate (100 mM)). Plates were then spun for 5 min at 1600 rpm. The injection port A on the sensor cartridge was loaded with 1 μM Oligomycin (Oligo), port B was loaded with 2 μM FCCP and port C was loaded with 0.5 μM Rotenone/Antimycin (ROT/AA). For sensor calibration, cells were incubated at 37C in a non-CO2 incubator, then plate was immediately placed onto calibrated XF96 extracellular flux analyzer for the Mito Stress test. For the Glycolysis Stress Test (ECAR) cells were resuspended in XF base media supplemented with L-glutamine (200 nM). The injection port A on the sensor cartridge was loaded with 10 mM glucose, port B with 2 μM oligomycin and 50 mM 2-DG was loaded in port C. Plate was incubated in 37C non-CO2 incubator during sensor calibration. Then, plate was loaded into calibrated XF96 extracellular flux analyzer for glycolysis stress test. For Seahorse analysis, equal number of cells were plated per well and oxygen-consumption measurements were normalized to total protein content measured by BCA assay after completion of the experiment. Thus, the reported OCR/ECAR values account for differences in cell recovery and cellular biomass between wells.

#### Immunofluorescence staining, imaging and analysis

HSCs were sorted in Opti-MEM media onto poly-L-lysine coated slides, then incubated for 1 h at 4C. Cells were then fixed with 4% paraformaldehyde for 20 min at room temperature (RT), permeabilized with 0.1% Triton X-100 and blocked with 2% bovine serum albumin. Cells were immuno-stained with Drp1(DNM1L) monoclonal antibody (1:100, Invitrogen #MA26255), Parkin polyclonal antibody (1:100, Invitrogen #PA13399), Pink1 polyclonal antibody (1:100 Invitrogen #PA1-16604), LC3B polyclonal antibody (1:100, Invitrogen #PA5-32254), Tom20 mouse monoclonal antibody (1:100, Santa Cruz, #sc17764), Stathmin 1 rabbit polyclonal antibody (1:50, Proteintech #11157-1-AP) or tubulin-alpha monoclonal antibody (1:100, BioLegend, #627910) in blocking buffer at RT for 1 h. Secondary staining in AF488 goat anti-mouse (Invitrogen, #A11001) and AF568 donkey anti-rabbit (Invitrogen, #A10042) were done after washes and incubated at RT for 1 h. Slides were mounted using anti-fade gold reagent with DAPI (Vectashield, #H-1200–10). z stack images were acquired with a Zeiss LSM 880 confocal microscope equipped with Airyscan Super Resolution Image module using a 63×/1.46 NA Alpha Plan Apochromat objective lens (Zeiss MicroImaging, Jena, Germany). Imaging measurements were quantified within segmented individual cells and protein intensity were normalized to cell area. Colocalization analyses were assessed using Pearson’s coefficient. For microtubule/mitochondria staining in HSCs, cells were then fixed with ice-cod methanol 10 min at room temperature (RT), permeabilized with 0.5% Triton X-100 for 10 min and blocked with 3% bovine serum albumin for 1 h at RT prior to immunostaining with mouse α-Tubulin (1:500, Sigma #DM1A) and rabbit Tom20 (1:500, Cell Signaling Technologies). Images were acquired using a 60×/1.42 NA Plan Apo Objective on an inverted Nikon Ti2 laser scanning microscope system equipped with a Nikon AXR galvanometric/resonance scanner and an ultra-low light NSPARC detector. All images were collected using Nyquist sampling with a Z-step of 0.172 μm per step.

Integrated fluorescence intensity density of images was calculated using Fiji software on unadjusted raw images. Background subtraction and adjustment of brightness were done on all sets of images avoiding low-end and high-end clipping of data. Colocalization was determined using Volocity using the colocalization feature or with FIJI using the COLOC2 feature. Puncta counts were done on Volocity software using the count objects feature, separating objects bigger than 3 μm. The 3D center of masses (CoMs) of microtubule and mitochondria were determined by first segmenting mitochondria and microtubules immunofluorescence in each plane of the z stack using Fiji. The 2D X-Y CoMs were then calculated for each plane by determining the weighted average x-y position of all the immunofluorescence intensities bounded by the segmented microtubule or mitochondria areas within a given image plane. The 3D X-Y CoM was calculated by the intensity weighted average of all the 2D X-Y CoMs from each plane of the z stack, whereas the Z CoM was calculated by weighting each plane by the microtubule or mitochondria intensities of each plane. The RMS difference between the microtubule and mitochondria CoMs for each cell is plotted in Figure 7.

#### Mitophagy flux

To determine mitophagy flux, HSCs were sorted into StemSpan SFEM media with TPO (100 ng/mL) and SCF (100 ng/mL), and Leupeptin (100 μM, ThermoFisher, Waltham, MA, USA) was added to the media for 2 h. Cells were then plated onto poly-L-lysine slides, allowed to rest for 1 h prior to fixation, permeabilization and LC3 immunostaining as described above. Mitophagy flux was calculated by subtracting the baseline levels of LC3 and leupeptin treated LC3 integrated density levels.

#### Cell sorting

Bone marrow cells were obtained by crushing bones in FACS Buffer. Cell suspensions were filtered using a 40-μm filter before staining with primary antibodies (Table S1) on ice for 40 min. Cells were enriched for c-Kit using a biotin-conjugated CD117, followed by streptavidin-conjugated magnetic beads using MACS gravity positive labeling (Miltenyi Biotec, Auburn, CA, USA) before double sorting Lineage-c-Kit+ Sca-1+ CD150+ CD48- HSCs on a BD FACSAria Fusion (BD Biosciences, Franklin Lakes, NJ, USA). Non-viable cells were excluded by 4′, 6-diamidino-2-phenylindole (DAPI) staining.

#### Cell cycle analysis

Bone marrow cells were stained with surface markers (Table S1) and fixed with BD Cytofix/Cytoperm (BD Biosciences). Cells were then blocked with 5% goat serum and stained with mouse anti-human Ki67 (clone B56; BD Pharmingen) per manufacturer’s instructions. Samples were washed and resuspended in DAPI- containing FACS buffer for analysis by flow cytometry. For G-CSF treatment, granulocyte-colony stimulation factor (G-CSF; Amgen, Thousand Oaks, CA, USA) was diluted in sterile phosphate-buffered saline (PBS) and injected subcutaneously (3.75 μg/100 μL) every 12 h for a total of 2 injections per mouse. Bone marrow was analyzed as above after 2 h following the last injection. For PAM_2_CSK_4_ (Invivogen, San Diego, CA, USA) treatment, PAM2 was diluted in PBS and injected intraperitoneally (100 μg) for 24 h, for a total of 1 injection per mouse. Control mice were injected with vehicle (PBS).

#### Apoptosis analysis

Bone marrow cells were isolated as described above, stained for surface markers, then washed with Annexin V binding buffer and staining for Annexin V using the Annexin V Apoptosis Detection Kit (eBioscience, San Diego, CA, USA) according to manufacturer instructions and resuspended in DAPI-containing FACs buffer for analysis by flow cytometry.

#### Homing assay

1 × 10^5^ LSK (Lineage-cKit+ Sca-1+) cells from WT and *Stmn1*^−/−^ mice (CD45.2) were sorted and transplanted retro-orbitally into lethally irradiated WT (CD45.1) mice. After 18 h, bone marrow was harvested from recipient mice and bone marrow chimerism was analyzed by flow cytometry.

#### 5-FU recovery analysis

Mice were weighed, peripheral blood was collected, and 5-fluorouracil (5-FU) was injected intraperitoneally (IP; 100 mg/kg) on day zero. Peripheral blood was collected every 2–3 days and complete blood counts were analyzed on the Element HT5 automated cell counter until blood counts returned to baseline levels.

#### MethoCult colony growth assays

Whole bone marrow cells (1 × 10^4^ cells) or sorted HSCs mice were incubated in MethoCult GF M3434 (Stem Cell Technologies, Vancouver, BC, Canada). Colonies were scored after 7 days at 37°C with 5% CO_2_. For serial replating analyses, wells were washed and replated at a density of 5 × 10^3^ cells per well every 7 days.

#### Spermidine treatment

Spermidine (Sigma-Aldrich, St. Louis, MO, USA) was injected IP (50 mg/kg) daily for 10 days 16 h following the last treatment, bone marrow was harvested for MethoCult growth assays and HSCs were sorted for LC3 analysis by confocal microscopy and Western blot.

#### Mitochondria membrane potential and mitochondrial ROS measurements

To measure mitochondrial membrane potential, whole bone marrow was stained with HSC markers. After washing, cells were stained with 50 nM tetramethylrhodamine (TMRM) dye at 37°C for 30 min. CCCP 5 μM (Invitrogen, Waltham, MA, USA) was used as a control, incubated at 37°C for 5 min. The TMRM signal was measured on a Cytek Aurora flow cytometer. For measuring mitochondrial superoxide production, whole bone marrow was stained with MitoSOX Red (Invitrogen) for 30 min at 37°C. Mitochondria ROS levels were obtained by Cytek Aurora flow cytometer using the MitoSOX channel. Mean fluorescence intensity (MFI) was calculated using FlowJo software. Fluorescence intensity was reported on a per cell basis. Therefore, the assay reflects the mitoSOX and membrane potential signal of an average viable cell, not normalized to mitochondrial mass.

#### RNA sequencing

HSCs were double sorted directly into lysis buffer. RNA was isolated using the NucleoSpin RNA XS Kit (Machery-Nagel, Bethlehem, PA, USA). Samples were then prepared according to library kit manufacturer’s protocol, indexed, pooled, and sequenced on an Illumina NovaSeq 6000. Base calls and demultiplexing were performed with Illumina’s bcl2fastq2 software. RNA-seq reads were aligned and quantitated to the Ensembl release 101 primary assembly with an Illumina DRAGEN Bio-IT server running version 3.9.3–8 software. All gene counts were then imported into the R/Bioconductor package EdgeR1 and TMM normalization size factors were calculated to adjust for differences in library size. Differential expression analysis results were filtered for genes with Benjamini-Hochberg false-discovery rate adjusted *p*-values less than or equal to 0.05. Global perturbations in known Gene Ontology (GO) terms, MSigDb, and KEGG pathways were detected using the R/Bioconductor package GAGE4. The R/Bioconductor package heatmap35 was used to display heatmaps across groups of samples for autophagy-related genes.

#### Microtubule network staining

For microtubule network staining, cells were sorted into StemSpan SFEM media with TPO (100 ng/mL) and SCF (100 ng/mL) and incubated for 24 h at 37C and 5% C02. Cells were then plated in poly-L-lysine coated glass coverslips, rested for 45 min at 37°C, washed with warm PBS, fixed with ice-cold methanol for 10 min at −20°C and washed with ice-cold PBS. They were then permeabilized in 0.5% v/v Triton X-100 (Pierce Biotechnology, Waltham, MA, USA) in PBS for 10 min before being blocked in 3% BSA in PBS with 0.05% Tween 20 (Millipore Sigma, Burlington, MA, USA). Primary antibodies were incubated in the block buffer for 1 h at RT before washing twice with 0.01% v/v Triton X-100 in PBS. Secondary antibodies and dyes were also incubated in the block buffer for 1 h at RT before being washed twice with 0.01% v/v Triton X-100 in PBS. Finally, coverslips were incubated with 0.25 μg/mL of DAPI for 10 min at room temperature before being washed twice with 0.01% v/v Triton X-100 in PBS and mounted in a solution of ProLong Glass Antifade mountant (Invitrogen) and allowed to cure for 24 h before imaging. Images were captured using a Nikon AXR confocal system on an Eclipse Ti2 inverted microscope equipped with a Nikon Spatial Array Confocal (NSPARC) detector using a 60× (1.42 NA) Plan-Apo oil immersion objective. Image stacks were captured using galvanometer (resonance) scanning at 16-bit with a resolution of 2048 × 2048, axial spacing of 0.2 μm, and a pinhole size of 2 using the Nikon Elements Software package. Image analysis was performed using the software Fiji.

#### Transmission electron microscopy

For ultrastructural analyses, 2 × 10^4^ sorted HSCs, 5 × 10^4^ sorted GMPs or 1 × 10^5^ sorted Gr1+ cells were fixed in 2% paraformaldehyde/2.5% glutaraldehyde (Ted Pella Inc., Redding, CA, USA) in 100 mM sodium cacodylate buffer for 2 h at room temperature. Samples were washed in sodium cacodylate buffer and post-fixed in 1% osmium tetroxide (Ted Pella Inc.) for 1 h at room temperature. Samples were then rinsed extensively in dH_2_0 prior to *en bloc* staining with 1% aqueous uranyl acetate (Electron Microscopy Sciences, Hatfield, PA, USA) for 1 h. Following several rinses in dH_2_0, samples were dehydrated in a graded series of ethanol, and embedded in Eponate 12 resin (Ted Pella Inc). Ultrathin sections of 95 nm were cut with a Leica Ultracut UCT ultramicrotome (Leica Microsystems Inc., Bannockburn, IL), stained with uranyl acetate and lead citrate, and viewed on a JEOL 1200 EX transmission electron microscope (JEOL USA Inc., Peabody, MA) equipped with an AMT 8 megapixel digital camera and AMT Image Capture Engine V602 software (Advanced Microscopy Techniques, Woburn, MA). Measurements of mitochondria cristae were done on ImageJ following the protocol previously described.^53^

#### Lentivirus production

For the mKeima lentivirus expression, the pHAGE-mt-mKeima plasmid (#131626, Addgene) was used. HEK293T cells (5 × 10^5^) were seeded in a 6-well plate 24 h before transfection and cultured in DMEM medium supplemented with 10% FBS. Cells were transfected with 2 μg of mKeima plasmid, 1.5 μg of psPAX2 packaging vector, 1 μg of VSG-G envelope plasmid, and 9 μL of PEI 25K. After 24 h of incubation, the medium was replaced with fresh DMEM. After 2 days of transfection, the lentivirus-enriched media were collected, and the plate was replaced with fresh media. Collected media was filtered with a 0.45 μm filter and stored at 4 °C. A second collection of virus was performed 24 h later. The mKeima lentivirus was used fresh for every transduction with c-Kit^+^ cells.

#### mKeima assay

For lentivirus transduction, c-Kit+ magnetic selected cells were seeded in a 24-well plate in a concentration of 500,000 cells per well in StemSpan media supplemented with thrombopoietin (TPO; 100 ng/mL, PeproTech, Cranbury, NJ, USA), stem cell factor (SCF; 100 ng/mL, PeproTech). Polybrene (10 μg/μL) was added to the cells, followed by 500 μL fresh mKeima lentivirus. Plates were centrifuged at 1600rpm for 2 h at RT. After, the plates were incubated in 37 °C. The next morning, the media were changed without disturbing the cells. Plates were incubated for 72 h for maximal mKeima expression. On the 3^rd^ day, cells were treated with CCCP (5 μM) for 6 h and/or Bafilomycin A (100 nM) for the final 2 h of incubation. Cells were collected, washed in PBS, and analyzed by flow cytometry. Flow cytometry was performed on a BD Aria that had dual excitation at 405 nm (pH7) and 561 nm (pH4) with both at 610/20 nm emission filter. DAPI was used to exclude cell death. The mt-Keima assay was analyzed as the percentage of mitochondria exhibiting an acidic lysosomal associated mt-Keima signal relative to the total mt-Keima positive population. Mitophagy was calculated by analysis of 561nm/405 nm ratio.^5^ Data processing was performed in FlowJo.

### Quantification and statistical analysis

Data are presented as mean ± standard deviation (SD), unless otherwise stated. In some cases, the data were log-transformed when the data skewed prior to statistical analysis. Unless otherwise stated, statistical significance was assessed using an unpaired two-tailed Student’s *t* test or two-way analysis of variance (ANOVA) for untransformed data and an unpaired Student’s *t* test with Welch’s correction for log-transformed data. GraphPad Prism (Version 9.3.1) was used for all statistical analyses (GraphPad Software, La Jolla, CA, USA). In all cases, ∗*p* < 0.05, ∗∗*p* < 0.01, ∗∗∗*p* < 0.001, and ∗∗∗∗*p* < 0.0001.
