# Supplementary material for: Stathmin 1 regulates mitophagy and non-proliferative cellular function in hematopoietic stem and progenitor cells
Source: iScience. 2026 Oct 14;29(11):117458. doi: 10.1016/j.isci.2026.117458 (PMC13683780; doi:10.1016/j.isci.2026.117458)
Supplement: Supplementary file 1 [file mmc1.pdf]

iScience, Volume *29*

Supplemental information

Stathmin 1 regulates mitophagy

and non-proliferative cellular function

in hematopoietic stem and progenitor cells

Luana Chiquetto, Meg Schuetz, Qian Dong, Mia Warmka, Liana Valin, Ashley Jones, Patrick Hunt, Copper Petermeier, Jie Wang, Nate Roundy, Zev J. Greenberg, Wei Yang, Christine R. Zhang, Grant A. Challen, Cliff J. Luke, Robert A.J. Signer, Wandy L. Beatty, Stephen Sykes, Weikai Li, David J. Kast, and Laura G. Schuettpelz

Supplemental information

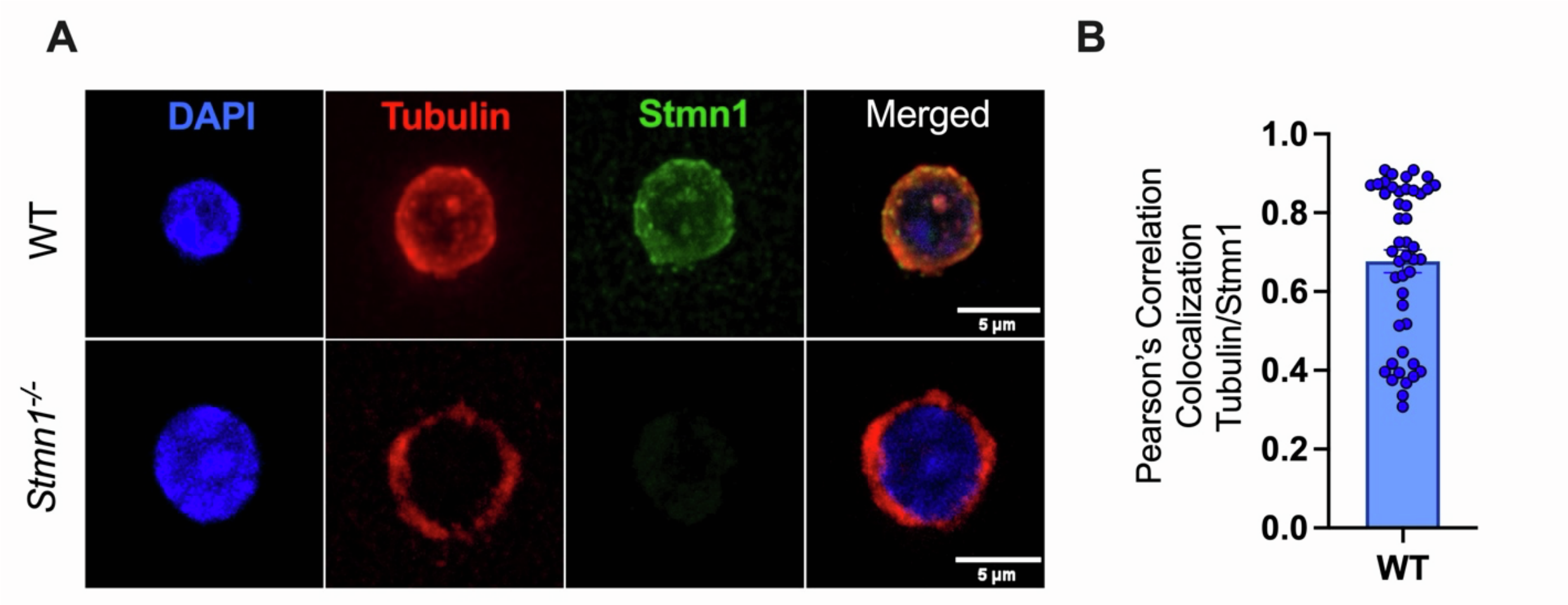

Figure S1. Stathmin 1 is expressed in HSCs and co-localizes with tubulin. (A) Shown are representative confocal immunofluorescence images of sorted HSCs (Lin- c-Kit+ Sca-1+ CD150+ CD48- cells) from WT and *Stmn1*$^{-/-}$ mice including DAPI (blue), alpha-tubulin (red), and Stathmin 1 (green), and colocalization of tubulin and stathmin 1 by Pearson's coefficient in WT cells **(B)**.

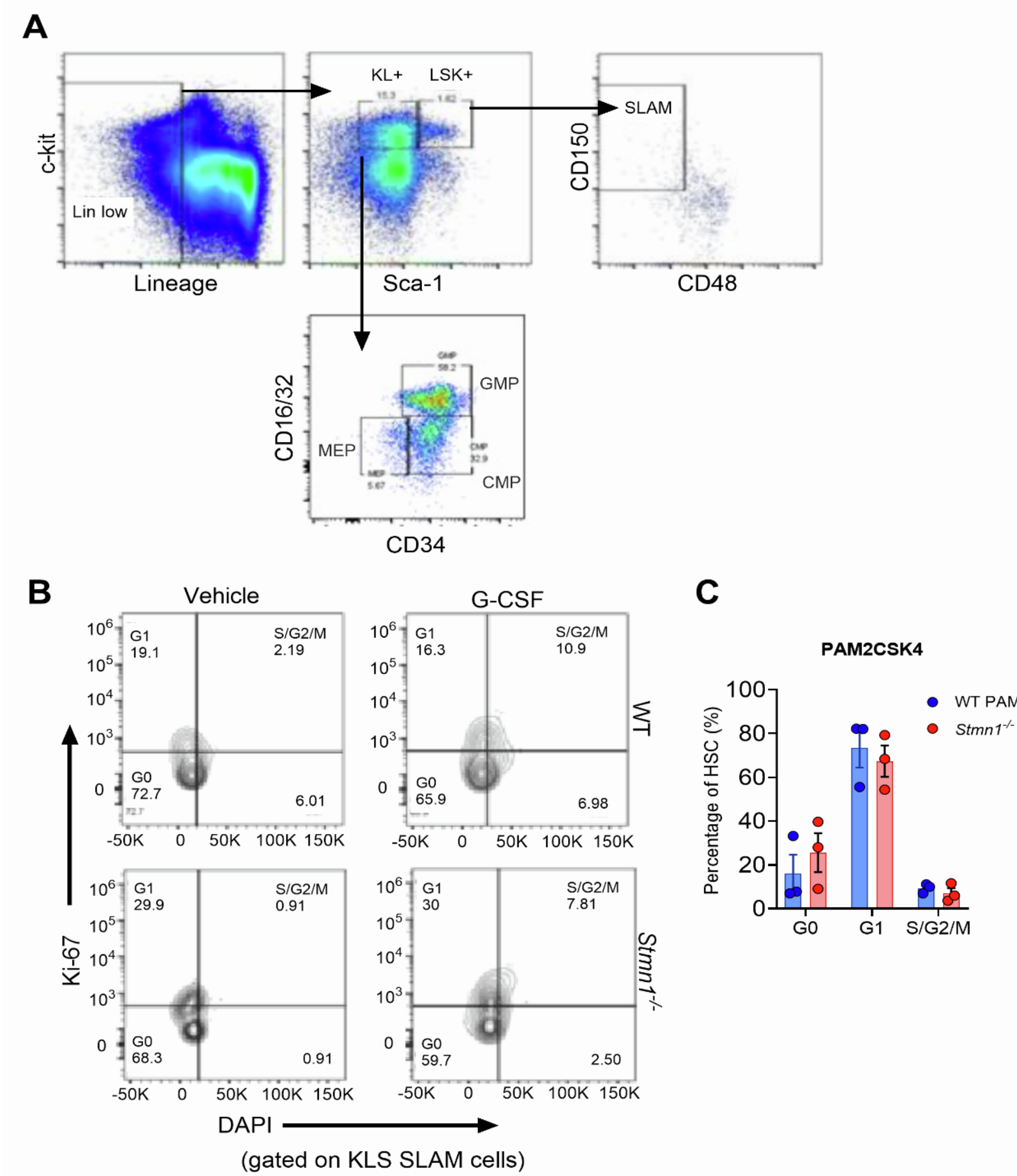

Figure S2. HSC and Ki67/DAPI gating strategies. (A) Representative flow plots of hematopoietic stem cell and myeloid progenitor gating strategy (related to main Figure 1E-H). **(B)** Representative flow plots of HSCs further gated for Ki-67 and DAPI from WT and *Stmn1*$^{-/-}$ young adult mice treated +/- G-CSF (related to main Figure 3G-I). **(C)** Cell cycling was measured by flow cytometry using Ki-67 and DAPI in WT and *Stmn1*$^{-/-}$ HSCs following one dose of PAM_2CSK_4 over 24 hours. Shown are the percentages of HSCs in G_0, G_1, and S/G_2/M. Data represent three biological replicates in two separate experiments.

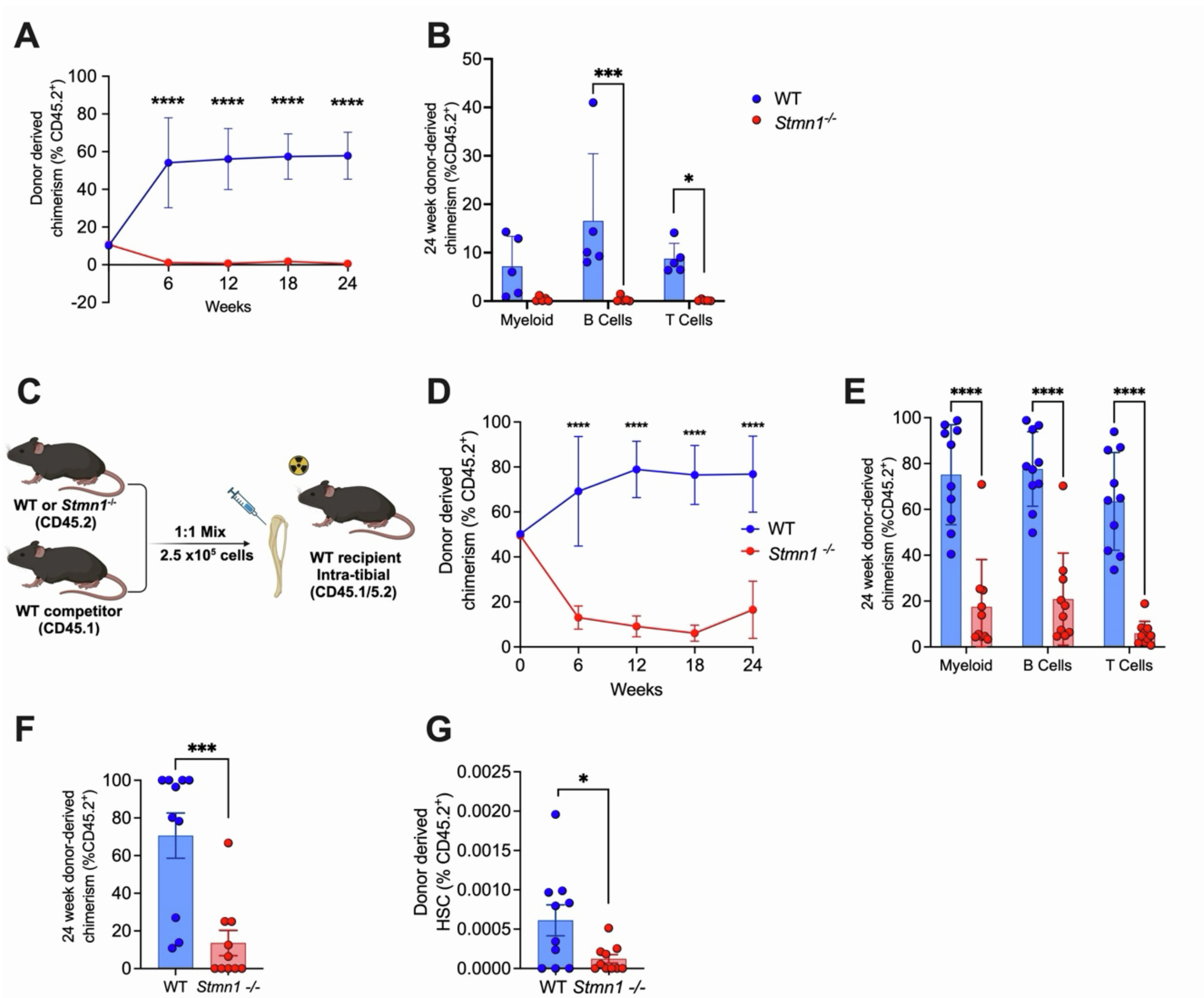

Figure S3. *Stmn1$^{-/-}$* HSCs have markedly impaired function. (A-B) WBM from primary transplant recipient mice as described in main Figure 2A-C was harvested after 24 weeks and transplanted into new lethally irradiated recipients. Chimerism was assessed by peripheral blood (PB) flow cytometry every 6 weeks. Shown are total %CD45.2 donor derived cells in the PB over time **(A)**, and tri-lineage engraftment in the PB at 24 weeks **(B)**. n= 5 mice per group. **(C)** Outline of intra-tibial competitive whole BM transplants. WBM from WT or *Stmn1$^{-/-}$* mice (CD45.2) was mixed 1:1 with WBM from a WT competitor (CD45.1) and 2.5 x 10^5 total cells were injected intra-tibially into lethally irradiated WT (CD45.1/CD45.2) recipients. Chimerism was assessed by PB flow cytometry every 6 weeks. Shown are total %CD45.2 donor derived cells in the PB over time **(D)**, tri-lineage engraftment in the PB at 24 weeks **(E)**, and %CD45.2 donor derived total cells **(F)** and HSCs **(G)** in the bone marrow at 24 weeks. n = 10 recipients per group over 2 independent transplants. Error bars represent mean +/- SD. *p<0.05, ***p<0.001, ****p<0.0001 by unpaired Student's t- test.

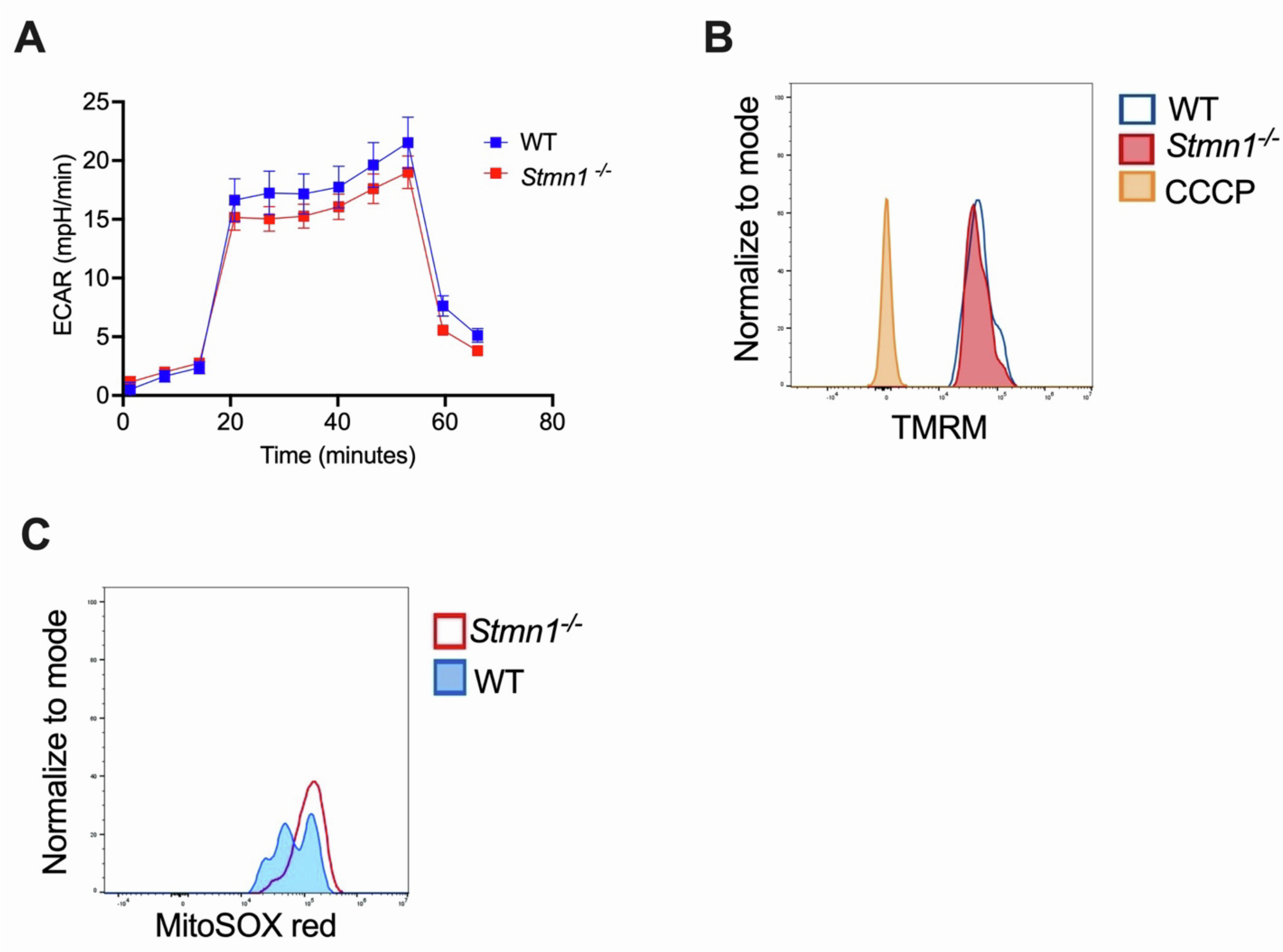

Figure S4. Glycolytic capacity is similar between *Stmn1*$^{-/-}$ and WT HSPCs. (A) Glycolytic capacity of c-Kit+ HSPCs from WT and *Stmn1*$^{-/-}$ mice were measured using the Seahorse Glycolysis Stress test. Shown are combined data from two independent experiments. **(B)** Representative histogram of HSC mitochondria membrane potential determined by flow cytometry using the tetramethylrhodamine (TMRM) dye. CCCP 5uM was used as a control (related to main Figure 5D). **(C)** Representative histogram of MitoSOX Red from WT and *Stmn1*$^{-/-}$ HSCs by flow cytometry (related to main Figure 5C).

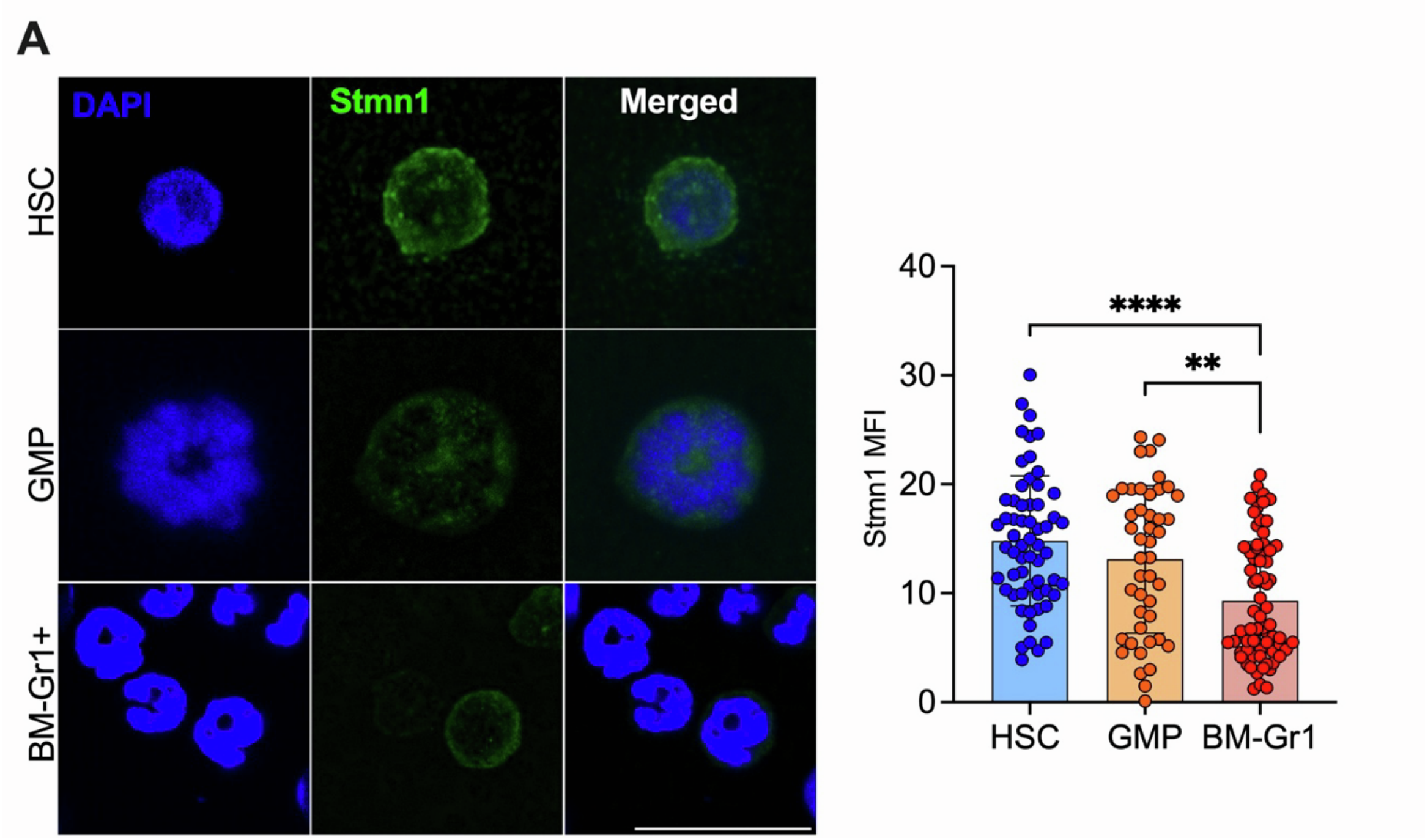

Figure S5. Stathmin 1 is expressed at lower levels in GMPs and Gr1+ cells compared to HSCs. Shown are representative images of sorted HSCs, GMPs, and Gr1+ granulocytes from the bone marrow of adult WT mice stained for stathmin 1 (green) and DAPI (blue), as well as quantification of Stmn1 mean fluorescence intensity (MFI) in those populations. **p<0.01, ****p<0.0001 by unpaired Student's t- test. Scale bar represents 5 µm.

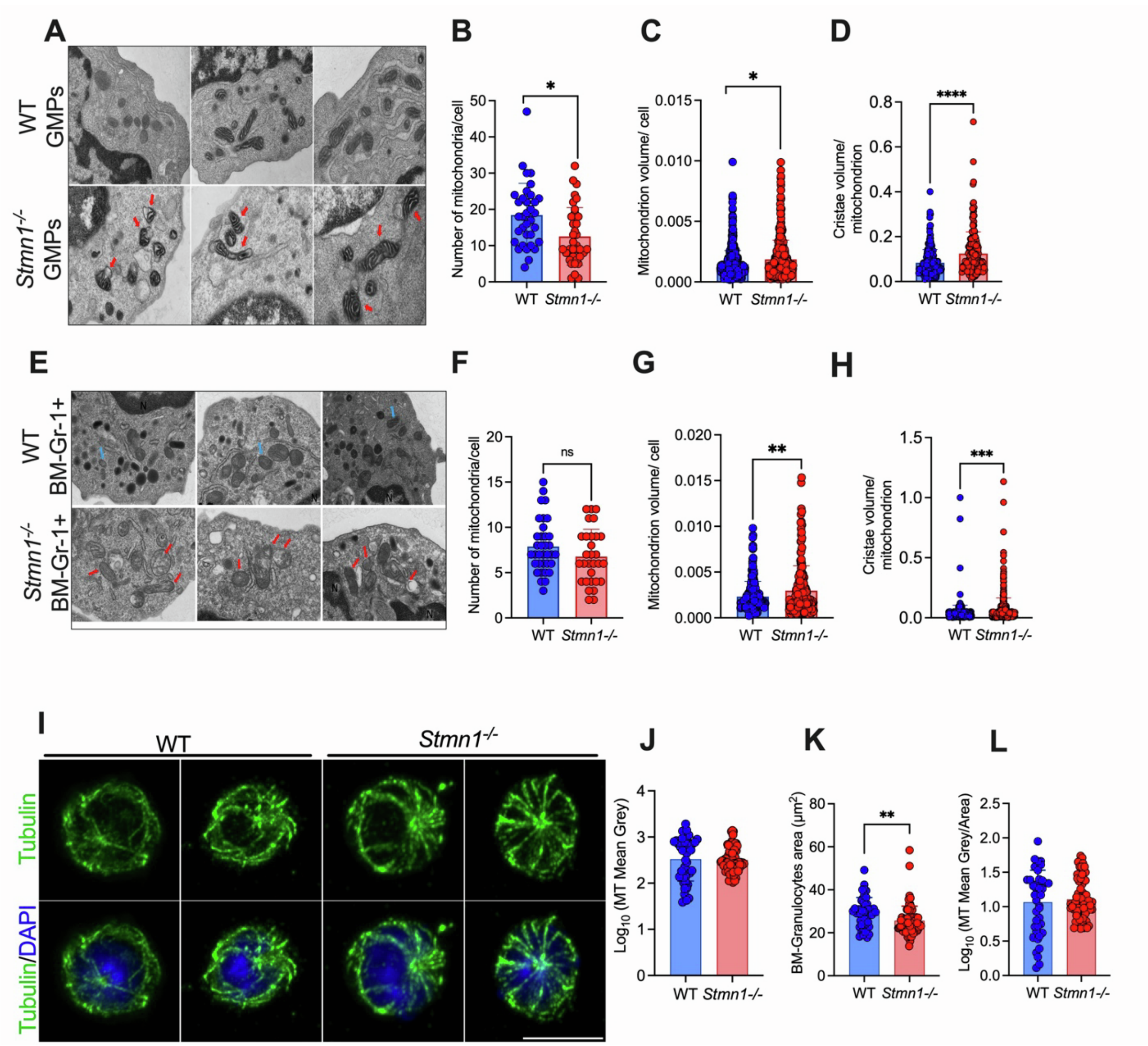

Figure S6. Mitochondrial abnormalities are also found in GMPs and Gr-1+ myeloid cells from *Stmn1*$^{-/-}$ mice. (A) Shown are representative Transmission Electron Microscopy (TEM) micrographs highlighting the mitochondria morphology in sorted GMP cells from WT (top row) and *Stmn1*$^{-/-}$ mouse bone marrow (bottom row; red arrows). **(B-D)** Quantification of individual mitochondria number per cell **(B)**, mitochondrial volume/cell **(C)**, and cristae volume per mitochondrion **(D)**. **(E)** Representative TEM micrographs of sorted Gr-1+ cells from WT and *Stmn1*$^{-/-}$ mouse bone marrow highlighting the mitochondria morphology in sorted Gr-1+ cells from WT (top row; blue arrows) and *Stmn1*$^{-/-}$ mice (bottom row; red arrows). **(E-H)** Quantification of mitochondria volume/cell **(F)**, cristae volume per mitochondrion **(G)**, and individual mitochondria number per cell **(H)**. **(I)**. Representative images of microtubule network in Ficoll-separated granulocytes from the bone marrow of WT and *Stmn1*$^{-/-}$ mice. Microtubules were stained with alpha-tubulin (green) and nucleus with DAPI (blue). **(J-L)** Quantification of tubulin intensity **(J)**, tubulin normalized by cell area **(K)**, and granulocyte area **(L).** Shown are combined data from two independent experiments. *p<0.05, **p<0.01, ***p<0.001, ****p<0.0001. Scale bar represents 5 μm.

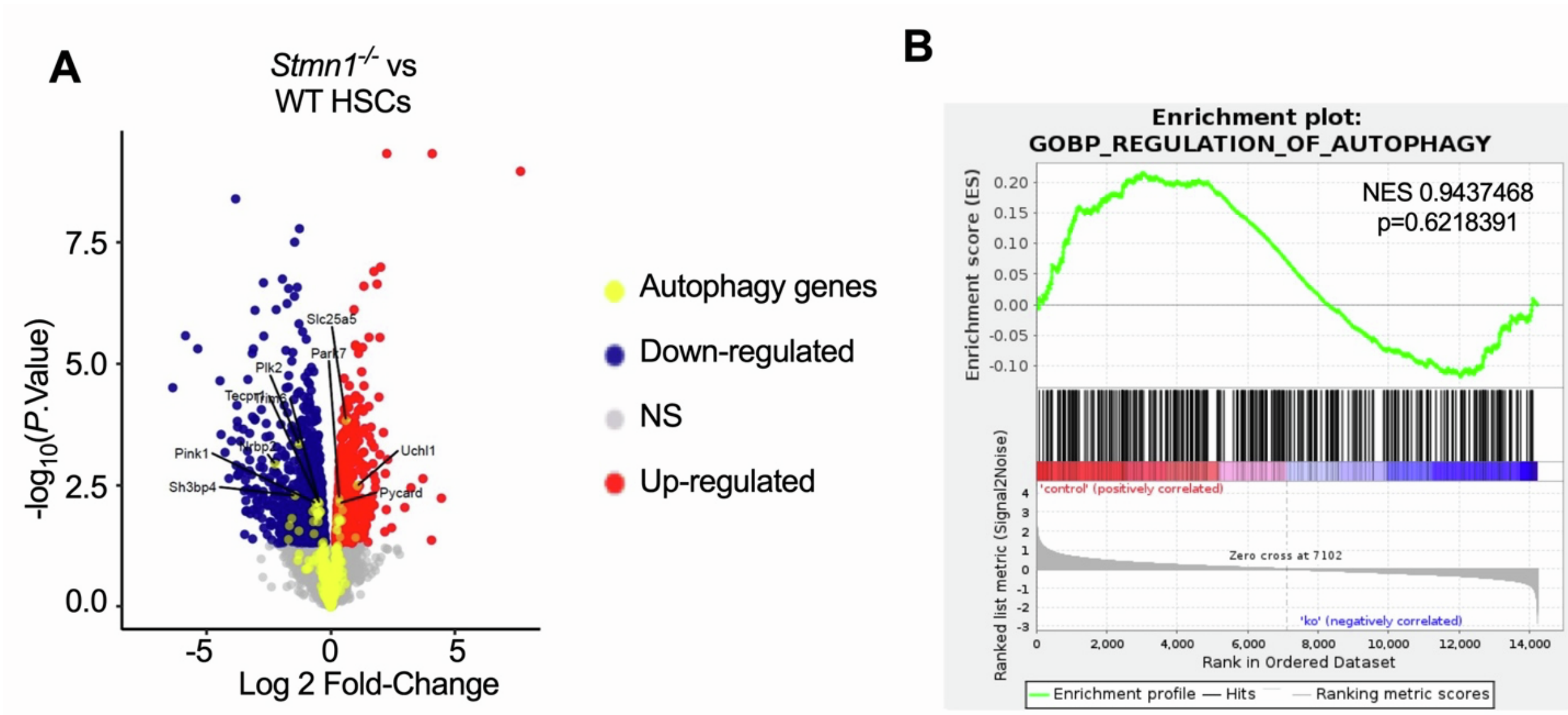

Figure S7. Most autophagy genes are not differentially expressed in *Stmn1$^{-/-}$* HSCs. (A) Volcano plot of differentially expressed genes between *Stmn1$^{-/-}$* and WT HSCs. Autophagy-related genes are highlighted in yellow. **(B)** Enrichment plot for autophagy pathway genes comparing *Stmn1$^{-/-}$* to WT HSCs.

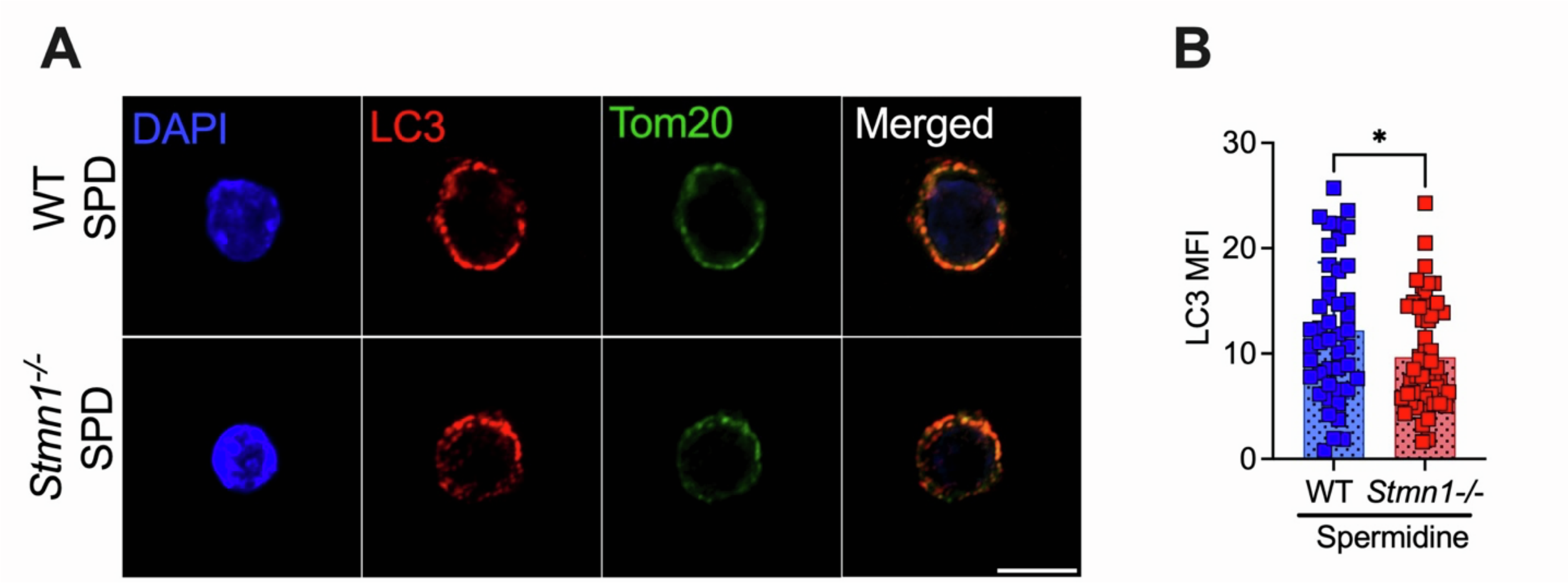

Figure S8. Spermidine induces mitophagy in WT (and to a lesser extent) *Stmn1$^{-/-}$* HSCs. (A) Representative confocal images of HSCs stained for LC3 and Tomm20 from WT and *Stmn1$^{-/-}$* mice treated with spermidine. **(B)** Quantification of LC3 MFI in HSCs from spermidine treated mice. Data represent the combination of two independent experiments. Error bars represent mean +/- SD. *$p<0.05$ by unpaired Student's t test. (Related to Figure 7 K). Scale bar represents 5 µm.

Table S1. Differentially expressed genes in *Stmn1*$^{-/-}$ vs WT HSCs

Feature_ID	external_gene_name	logFC	CI.L	CI.R	AveExpr	t	P.Value	adj.P.Val	B	linearFC	sample.ko5	sample.ko4	sample.ko3	sample.ko2	sample.ko1	sample.wt5	sample.wt4	sample.wt3	sample.wt2	sample.wt1
ENSMUSG00000(	Gm10036	4.08E+00	3.39E+00	4.77E+00	3.180833	1.24E+01	4.74E-10	3.32E-06	12.40647	16.90418	4.798918431	4.82465511	5.84765914	5.150549482	4.91043252	1.73684548	0.906823109	0.36384405	2.167975466	1.10062321
ENSMUSG00000(	Gm10282	2.25E+00	1.87E+00	2.64E+00	6.403425	1.24E+01	4.72E-10	3.32E-06	13.36993	4.7711	8.020511925	6.82928207	7.554847311	7.94618523	7.39654991	5.48766528	5.512240199	5.29848121	4.63409893	5.35438887
ENSMUSG00000(	Gm6594	7.63E+00	6.26E+00	9.00E+00	-2.08136	1.17E+01	1.09E-09	5.09E-06	7.480898	198.3961	1.277714765	0.83183667	3.244222457	1.922291866	1.18104763	-5.83861813	-6.012516974	-5.98736022	-5.53010407	-5.90207931
ENSMUSG00000(	Fscn1	-3.83E+00	-4.57E+00	-3.08E+00	3.199571	-1.08E+01	4.02E-09	1.41E-05	10.04485	-14.1807	0.748138506	1.6540833	1.449748827	1.445788954	0.80013851	5.44389117	5.028457625	4.86048013	5.473413841	5.09156675
ENSMUSG00000(	Art4	-1.26E+00	-1.53E+00	-9.88E-01	6.440894	-9.81E+00	1.66E-08	4.65E-05	9.823734	-2.39154	5.546434925	6.14579263	6.065420532	5.562263817	5.77514728	7.07931331	7.005161403	6.93355084	7.112624573	7.18322716
ENSMUSG00000(	Ndn	-1.45E+00	-1.78E+00	-1.13E+00	5.580836	-9.40E+00	3.13E-08	7.32E-05	9.235096	-2.73401	4.680581589	4.99975208	4.60704622	4.505512732	5.60888532	6.13973482	6.198458704	6.14224479	6.613598006	6.31254338
ENSMUSG00000(	Selp	2.01E+00	1.52E+00	2.49E+00	5.928981	8.66E+00	1.03E-07	2.05E-04	8.002421	4.015091	6.377320844	7.20797273	6.595687297	7.388937879	6.44059582	5.19750052	4.442728039	4.61748948	5.513292386	5.50828724
ENSMUSG00000(	Sell	1.74E+00	1.31E+00	2.16E+00	6.24952	8.54E+00	1.26E-07	2.21E-04	7.762577	3.330846	7.404581799	7.04161771	6.950375942	7.477705804	6.34696346	5.83513261	4.968336633	5.35749046	5.134343214	5.97865212
ENSMUSG00000:	Pde2a	-1.94E+00	-2.43E+00	-1.45E+00	5.02294	-8.32E+00	1.80E-07	2.81E-04	7.53265	-3.84229	3.269759181	4.78895252	4.12569756	3.591290481	5.03016977	6.12538332	5.833145563	6.07321413	5.608679686	5.78310749
ENSMUSG00000(	Hk3	1.33E+00	9.86E-01	1.68E+00	6.003568	8.12E+00	2.53E-07	2.84E-04	7.077412	2.516078	6.701614968	6.93716127	5.971669958	6.92466767	6.37286434	5.22275306	5.19388694	5.49997739	5.945122707	5.26596596
ENSMUSG00000(	Stmn1	-1.35E+00	-1.70E+00	-9.97E-01	9.085948	-8.09E+00	2.67E-07	2.84E-04	6.915655	-2.54427	8.811725952	7.91060152	8.591821838	8.75358306	8.16774097	10.0294033	10.03872809	9.80497001	8.912212255	9.83869492
ENSMUSG00000(	Tns2	-2.70E+00	-3.40E+00	-2.01E+00	4.128156	-8.22E+00	2.15E-07	2.84E-04	7.288852	-6.5203	1.869324852	3.70024075	2.735392804	2.061557501	3.61490138	5.60173199	5.364151088	5.12963345	6.133009237	5.07161806
ENSMUSG00000(	Cd53	1.86E+00	1.38E+00	2.34E+00	5.346511	8.18E+00	2.29E-07	2.84E-04	7.23214	3.642281	6.42696549	5.60263387	6.193417258	6.781404225	5.95737163	4.95985367	3.901868159	4.34019242	4.531942067	4.76946176
ENSMUSG00000(	Obscn	-1.70E+00	-2.14E+00	-1.25E+00	5.319565	-8.06E+00	2.83E-07	2.84E-04	7.070911	-3.23846	3.901442413	5.0801976	4.033138907	4.10984609	5.43669781	6.16384531	6.075277331	6.0937903	6.504351024	5.79705931
ENSMUSG00000(	Mmrn1	-1.46E+00	-1.85E+00	-1.07E+00	7.881127	-7.85E+00	4.10E-07	3.83E-04	6.511782	-2.75072	6.831000842	7.99935428	7.188304752	6.531724649	7.66069425	8.4524806	8.539371493	8.72886978	8.385681963	8.49378949
ENSMUSG00000(	Sult1a1	-1.76E+00	-2.24E+00	-1.27E+00	5.197546	-7.65E+00	5.79E-07	5.07E-04	6.366775	-3.37689	3.384239855	4.95447629	4.199388251	4.350602234	4.94015068	6.33908792	5.812036101	6.20143877	5.911895975	5.88214655
ENSMUSG00000(	Arhgef28	-3.05E+00	-3.90E+00	-2.19E+00	1.971747	-7.47E+00	7.95E-07	5.86E-04	5.452728	-8.25469	0.862471181	1.20191156	-0.31578592	1.269362387	-1.35042117	3.76315265	3.81879027	3.75579117	3.254530775	3.45767025
ENSMUSG00000(	Padi4	9.36E-01	6.73E-01	1.20E+00	7.590504	7.49E+00	7.70E-07	5.86E-04	5.859388	1.913403	7.960703833	8.46599432	7.819552849	8.054874663	7.9474122	7.40856767	6.949836835	7.1433712	7.120723303	7.03400647
ENSMUSG00000(	Igf2bp2	-2.19E+00	-2.81E+00	-1.58E+00	3.208197	-7.49E+00	7.68E-07	5.86E-04	6.035671	-4.57649	1.424350069	1.60137625	3.065805096	2.6260559	1.9772662	4.36961622	4.376576548	4.55856955	3.991496369	4.09085903
ENSMUSG00000(	Dpp4	-1.28E+00	-1.66E+00	-8.99E-01	4.474195	-7.12E+00	1.51E-06	1.06E-03	5.453755	-2.42372	3.976362148	3.73582645	3.193093416	4.053066041	4.41192765	5.26751291	5.138715256	5.06748825	4.804169214	5.09378675
ENSMUSG00000(	Apbb1	-1.13E+00	-1.48E+00	-7.87E-01	4.830286	-6.92E+00	2.20E-06	1.47E-03	5.056829	-2.19153	3.969999645	4.55795164	4.368534133	3.917434931	4.75810329	5.44157266	5.441268082	5.24085722	5.348713214	5.25842244
ENSMUSG00000(	6330403K07Rik	-5.84E+00	-7.65E+00	-4.04E+00	-0.71564	-6.82E+00	2.65E-06	1.61E-03	2.33149	-57.3537	-4.26681184	-1.50296041	-5.807639016	-5.67315212	1.03914564	1.61259298	1.708582215	1.6492644	1.904524157	2.18006973
ENSMUSG00000(	Lat2	1.54E+00	1.06E+00	2.02E+00	3.444053	6.78E+00	2.87E-06	1.61E-03	4.856706	2.905563	4.477663245	3.95234709	4.803902255	4.059931013	3.62737162	3.03703908	2.317772509	2.83766214	2.631985091	2.69485583
ENSMUSG00000(	Gimap4	1.97E+00	1.36E+00	2.59E+00	4.152187	6.78E+00	2.88E-06	1.61E-03	4.818282	3.928235	4.664910613	5.12522993	5.122359046	5.358204477	5.40820181	1.76871218	3.796447201	3.22452807	3.676910249	3.37637015
ENSMUSG00000(	Gm27022	-2.69E+00	-3.53E+00	-1.86E+00	2.122957	-6.81E+00	2.70E-06	1.61E-03	4.449844	-6.46742	0.473396176	0.42849573	0.586051748	0.719165304	1.52016056	3.40650669	3.447037597	3.238522	4.399479832	3.01075016
ENSMUSG00000(	Mpl	-9.85E-01	-1.29E+00	-6.77E-01	10.06902	-6.73E+00	3.13E-06	1.69E-03	4.39249	-1.97911	9.221572287	10.1943373	9.53349754	9.1603804	9.9673306	10.4291378	10.45437202	10.5483252	10.74327267	10.4379968
ENSMUSG00000(	Fbp1	9.96E-01	6.78E-01	1.31E+00	5.025128	6.59E+00	4.12E-06	2.14E-03	4.35899	1.994404	5.496400531	5.48208456	5.005340455	5.78166123	5.74085553	4.6681853	4.38942915	4.41245174	4.600466492	4.67440504
ENSMUSG00000(	Socs2	9.93E-01	6.74E-01	1.31E+00	6.275646	6.56E+00	4.35E-06	2.18E-03	4.170382	1.990895	6.884314667	6.40480997	6.632534584	6.779073675	7.23664373	6.07987246	5.390761402	5.83799612	5.678398557	5.83205447
ENSMUSG00000(	Gem	1.28E+00	8.66E-01	1.69E+00	4.820832	6.53E+00	4.63E-06	2.23E-03	4.267998	2.426146	5.779857469	5.72239165	5.38896311	5.072522205	5.46771359	4.37933956	3.783522635	4.64654319	3.991496369	3.9759716
ENSMUSG00000(	Mettl24	-5.35E+00	-7.08E+00	-3.61E+00	-2.20051	-6.50E+00	4.93E-06	2.24E-03	1.868663	-40.7273	-5.85177434	-1.19262029	-5.807639016	-5.67315212	-5.99427736	0.79073849	0.137230146	0.52043442	0.94562936	0.1202885
ENSMUSG00000(	Gm11223	-3.11E+00	-4.12E+00	-2.10E+00	2.846773	-6.49E+00	4.95E-06	2.24E-03	4.213223	-8.6477	1.24667891	0.03865355	3.22902915	1.524752578	0.92148254	4.74561156	4.757568734	4.11605873	3.237021006	4.65087791
ENSMUSG00000(	Tinagl1	-1.80E+00	-2.39E+00	-1.22E+00	5.176163	-6.46E+00	5.31E-06	2.33E-03	4.17772	-3.49361	3.122640253	5.12639621	4.419976927	3.734115645	5.30435805	6.05884921	6.070629897	5.98812953	6.424455776	5.51207737
ENSMUSG00000(	Nkg7	1.12E+00	7.49E-01	1.49E+00	5.885028	6.38E+00	6.12E-06	2.45E-03	3.849591	2.170845	6.8449759	5.95154453	6.149100129	6.935795696	6.19709036	5.64871948	5.396343462	5.2030818	5.034998007	5.48862706
ENSMUSG00000(	Kazald1	-1.53E+00	-2.03E+00	-1.03E+00	4.441602	-6.41E+00	5.85E-06	2.45E-03	4.139315	-2.88592	2.879544694	3.84895056	3.578223385	3.746808059	4.10769831	5.32314361	5.095353941	5.03431382	6.044962394	4.75702465
ENSMUSG00000(	Ncam1	-3.16E+00	-4.20E+00	-2.11E+00	2.268986	-6.38E+00	6.13E-06	2.45E-03	3.807573	-8.91155	0.170593476	-0.08072741	1.134875489	1.041093399	1.5371041	3.0832228	3.97758699	4.25462293	3.757608309	3.81388268
ENSMUSG00000(	Mtss2	-1.57E+00	-2.10E+00	-1.03E+00	3.459532	-6.20E+00	8.85E-06	3.44E-03	3.781271	-2.96335	2.831220247	3.17285553	2.825356181	1.826693768	3.01974311	3.94110122	4.271728776	4.25223831	4.202911251	4.25147272
ENSMUSG00000(	Paqr7	-7.80E-01	-1.05E+00	-5.08E-01	6.709672	-6.05E+00	1.19E-05	4.49E-03	3.137455	-1.71692	6.150689102	6.66676421	6.343694703	6.039804703	6.6578954	7.03885773	6.977054925	7.28074311	6.904263531	7.03694887
ENSMUSG00000(	Serpinf1	-8.96E-01	-1.21E+00	-5.78E-01	6.628291	-5.94E+00	1.49E-05	5.21E-03	2.915685	-1.86143	6.1478734	6.21210927	5.945995603	6.379755509	6.06269898	7.53601385	7.032047909	6.9313167	7.198454227	6.83664631
ENSMUSG00000(	Maged2	-6.82E-01	-9.23E-01	-4.40E-01	6.627621	-5.95E+00	1.46E-05	5.21E-03	2.933645	-1.60409	6.216668079	6.46536079	6.152724993	6.212925466	6.50382389	6.81937014	7.044290062	7.10853124	6.963501236	6.78900988
ENSMUSG00000(	Siglecg	1.24E+00	8.03E-01	1.68E+00	3.438496	5.94E+00	1.47E-05	5.21E-03	3.26809	2.368482	4.655029101	3.92866255	4.012508028	4.12288749	3.24173683	2.89947413	2.817205761	2.79071691	2.957735963	2.9590076
ENSMUSG00000(	Ephb4	-1.69E+00	-2.30E+00	-1.08E+00	2.625451	-5.85E+00	1.75E-05	5.99E-03	3.097698	-3.23186	1.869324852	1.54667036	1.486981733	1.675576035	2.6019124	3.25677889	3.618660082	3.58059585	3.506069542	3.11194116
ENSMUSG00000(	Adam11	-1.04E+00	-1.41E+00	-6.62E-01	4.128689	-5.82E+00	1.88E-05	6.27E-03	2.996411	-2.05196	3.233034051	4.03433566	3.686216433	3.655522808	3.52732308	4.70538032	4.813236859	4.57392772	4.457159941	4.60075249
ENSMUSG00000(	Taldo1	5.41E-01	3.44E-01	7.38E-01	8.292598	5.79E+00	2.00E-05	6.52E-03	2.521893	1.454946	8.626748831	8.44747535	8.417490077	8.707106002	8.52736234	8.05477821	8.035968197	7.93494542	8.050269926	8.12383885
ENSMUSG00000(	H19	-3.33E+00	-4.55E+00	-2.11E+00	0.18722	-5.76E+00	2.11E-05	6.72E-03	1.717889	-10.0577	-2.39234272	-1.50296041	-0.522236797	-1.76626152	-0.86499434	1.96228177	1.814031514	1.5748822	1.764516678	1.80527982
ENSMUSG00000(	Ntf5	-4.45E+00	-6.09E+00	-2.82E+00	-1.53051	-5.73E+00	2.25E-05	7.01E-03	1.08079	-21.9052	-5.85177434	-2.9768916	-2.348207397	-4.08818962	-3.67234926	0.66917651	0.781898893	0.12116423	1.853600221	0.20644515
ENSMUSG00000(	Itsn1	-9.17E-01	-1.26E+00	-5.78E-01	5.909803	-5.70E+00	2.38E-05	7.25E-03	2.527645	-1.88832	5.155953778	5.77390842	5.835766261	4.886224972	5.97474934	6.32723101	6.329835536	6.35332443	6.239320571	6.22171878
ENSMUSG00000(	Vdr	-8.53E-01	-1.17E+00	-5.35E-01	5.602607	-5.66E+00	2.62E-05	7.82E-03	2.455538	-1.80596	4.943453629	5.51451616	5.683713057	5.074201711	4.63780906	6.08061765	6.000456675	6.22726246	5.925736838	5.93830524
ENSMUSG00000(	Fyb	7.47E-01	4.67E-01	1.03E+00	6.177173	5.62E+00	2.84E-05	8.17E-03	2.267458	1.678076	6.468371832	6.28531812	7.01534202	6.751287143	6.30138828	5.90543361	5.940755542	5.7469006	5.405753424	5.95118345
ENSMUSG00000(	Hbb-bt	1.33E+00	8.33E-01	1.83E+00	6.161815	5.61E+00	2.86E-05	8.17E-03	2.262897	2.520896	6.81366818	6.16571472	6.477948152	7.893995897	6.26779399	5.56455371	5.533403145	5.59961088	5.590950195	5.71051278
ENSMUSG00000(	Smtnl1	-1.70E+00	-2.34E+00	-1.06E+00	3.126239	-5.59E+00	3.01E-05	8.43E-03	2.620835	-3.24504	1.514547878	2.06750253	2.242209533	1.963472502	3.98728992	3.67905125	3.770481235	4.02526432	4.416802203	3.59577253
ENSMUSG00000(	Rhbdf1	-1.80E+00	-2.48E+00	-1.12E+00	3.322123	-5.57E+00	3.15E-05	8.48E-03	2.578268	-3.47418	1.287777016	3.2857253	3.250352707	1.858229342	2.45280587	3.94438008	4.188381631	4.2829351	4.754141679	3.91650287
ENSMUSG00000(	Meg3	-6.35E+00	-8.75E+00	-3.95E+00	2.343223	-5.57E+00	3.11E-05	8.48E-03	1.941197	-81.7308	-1.60384682	-3.8248885	1.885847941	-0.3877499	0.29887238	5.993362	5.66310492	4.57006518	5.110592767	5.72686739
ENSMUSG00000(	Tns1	-7.88E-01	-1.09E+00	-4.86E-01	6.103418	-5.51E+00	3.57E-05	9.44E-03	2.092036	-1.72643	5.543224178	5.97504065	5.819438824	5.205665166	6.14367438	6.47738049	6.318119851	6.41645071	6.713367467	6.42181987
ENSMUSG00000(	Pdgfd	-1.26E+00	-1.74E+00	-7.74E-01	3.486854	-5.48E+00	3.77E-05	9.77E-03	2.37884	-2.39058	2.52760403	3.24335236	2.795987329	3.463838993	1.85747168	4.09359662	4.222300457	4.16619181	4.137007471	4.36118989
ENSMUSG00000(	Cracr2b	-1.34E+00	-1.86E+00	-8.19E-01	4.114932	-5.44E+00	4.12E-05	1.05E-02	2.24868	-2.52695	2.698510712	3.84311713	3.029168792	3.452261352	4.56926215	5.12116651	4.685006665	4.64701523	4.606528245	4.49728407
ENSMUSG00000(	H2bc4	1.01E+00	6.13E-01	1.40E+00	5.557954	5.39E+00	4.53E-05	1.13E-02	1.890665	2.008001	6.30957173	5.58973755	5.513917086	5.94085829	6.6803872	4.66409435	4.812012235	5.05425786	6.005783641	5.00892311
ENSMUSG00000(	Rtkn	-1.09E+00	-1.51E+00	-6.60E-01	4.938614	-5.37E+00	4.71E-05	1.16E-02	1.992075	-2.12279	4.084686935	5.04321793	4.474569094	3.898828001	4.6106094	5.26109787	5.461624968	5.69740593	5.69009285	5.16400988
ENSMUSG00000(	Slco2a1	1.94E+00	1.18E+00	2.70E+00	2.263219	5.35E+00	4.89E-05	1.18E-02	2.163788	3.829704	2.810003761	3.64922301	2.916874837	3.340868351	2.77059423	1.49229874	0.177307585	1.30726053	1.836218143	2.33154037
ENSMUSG00000(	Klk8	-8.83E-01	-1.23E+00	-5.35E-01	5.033149	-5.35E+00	4.96E-05	1.18E-02	1.913861	-1.8439	4.626995283	3.97074859	4.647708755	4.631769551	4.9504347	5.42114866	5.37256888	5.24637927	5.939028949	5.52470958
ENSMUSG00000(	Prss57	1.17E+00	7.03E-01	1.63E+00	4.073055	5.31E+00	5.35E-05	1.21E-02	1.924	2.2431	4.653640273	4.36639778	4.286280268	5.285965964	4.07852518	3.68983598	3.410598936	3.1780493	4.017715886	3.76354085
ENSMUSG00000(	Cd69	8.91E-01	5.38E-01	1.25E+00	4.798792	5.31E+00	5.37E-05	1.21E-02	1.81977	1.854891	5.261316647	5.23310322	5.267171445	5.036793261	5.59396879	4.24486119	4.397934378	3.97119249	4.165124221	4.81645357
ENSMUSG00000(	Gm10053	6.85E-01	4.13E-01	9.58E-01	6.815659	5.30E+00	5.45E-05	1.21E-02	1.560814	1.608224	7.566254813	6.70615759	7.124579427	7.333286173	6.81836125	6.52671462	6.531568441	6.46709331	6.650557263	6.43201516
ENSMUSG00000:	Snrpn	-1.48E+00	-2.06E+00	-8.90E-01	2.860421	-5.30E+00	5.45E-05	1.21E-02	2.063852	-2.7829	1.632041441	2.09635739	1.857696901	2.343656169	3.188117	3.45347339	3.658139275	3.87217456	3.116922562	3.38563307
ENSMUSG00000(	Pkn3	-9.57E-01	-1.34E+00	-5.72E-01	4.59859	-5.23E+00	6.26E-05	1.36E-02	1.741272	-1.94085	4.23966105	4.497941	3.908322974	3.906163819	4.39481616	4.84525373	5.315596921	5.07805591	4.5718716	5.22822096
ENSMUSG00000(	Tle2	-8.44E-01	-1.18E+00	-5.04E-01	5.247591	-5.23E+00	6.31E-05	1.36E-02	1.637808	-1.79467	4.464507195	5.42919528	4.867435904	4.544805579	4.80095061	5.58817076	5.709863662	5.49897203	5.875569261	5.6964385
ENSMUSG00000(	Gdf3	6.35E-01	3.78E-01	8.93E-01	5.983803	5.20E+00	6.73E-05	1.43E-02	1.422374	1.553443	6.31281838	6.22577644	6.141093224	6.536605838	6.02913049	5.53913466	5.647033023	5.59994851	5.890382542	5.91610339
ENSMUSG00000(	Hid1	-9.39E-01	-1.32E+00	-5.58E-01	5.381706	-5.18E+00	6.96E-05	1.45E-02	1.526592	-1.91742	4.43015569	5.64149391	4.619673828	4.613405643	5.49907776	5.66868099	5.905973619	5.79194875	5.844392075	5.80225666
ENSMUSG00000(	Dlk1	-3.77E+00	-5.31E+00	-2.23E+00	-0.32963	-5.17E+00	7.20E-05	1.46E-02	0.537626	-13.6426	-2.39234272	-4.5618541	-1.559711503	-1.9727124	-0.06354002	1.84086197	1.318399904	1.27002762	1.546711526	1.27782978
ENSMUSG00000:	Gm42031	-1.87E+00	-2.63E+00	-1.11E+00	2.261776	-5.17E+00	7.20E-05	1.46E-02	1.781848	-3.6533	2.41931513	0.58735107	1.463263633	1.3498814	0.41850417	3.02940184	3.332932629	2.95039611	3.439944283	3.62676546
ENSMUSG00000(	Itgb2	6.95E-01	4.10E-01	9.79E-01	5.807476	5.15E+00	7.51E-05	1.48E-02	1.333659	1.618392	6.185429405	6.33170025	5.759841785	6.327200059	5.87493089	5.39799407	5.422632365	5.30783473	5.743108739	5.72408601
ENSMUSG00000(	Cavin2	-8.35E-01	-1.18E+00	-4.94E-01	4.699868	-5.15E+00	7.48E-05	1.48E-02	1.546267	-1.78391	4.420855448	4.13742915	3.805229481	4.364394835	4.90885132	5.09728355	5.119982756	5.03985466	4.826347899	5.2784515
ENSMUSG00000(	Pvt1	1.53E+00	9.03E-01	2.16E+00	2.46722	5.14E+00	7.62E-05	1.48E-02	1.749434	2.886914	2.303043772	3.58280415	3.497784373	3.194126621	3.48060479	1.14665458	1.624107647	1.88915672	2.309099717	1.64481515
ENSMUSG00000(	Ehd3	-1.09E+00	-1.54E+00	-6.43E-01	4.823096	-5.13E+00	7.83E-05	1.50E-02	1.511294	-2.12978	3.516732125	4.45402552	4.497282653	3.954381766	5.27543376	4.95660983	5.303198684	5.44047638	5.603680338	5.22913459
ENSMUSG00000(	Ncf4	7.21E-01	4.25E-01	1.02E+00	4.508061	5.12E+00	7.94E-05	1.50E-02	1.476031	1.648854	4.925480979	5.00736861	4.594307108	4.86697592	4.72425552	4.18790531	4.054917387	4.10407516	4.496419372	4.11890063
ENSMUSG00000(	Plac8	1.21E+00	7.10E-01	1.71E+00	6.286432	5.11E+00	8.20E-05	1.52E-02	1.191163	2.309946	6.716419317	6.45796826	6.83057061	7.531571845	6.33691952	5.97476225	5.180392245	5.54353376	5.898779481	6.39340266
ENSMUSG00000(	P2ry14	6.10E-01	3.58E-01	8.61E-01	5.837574	5.11E+00	8.23E-05	1.52E-02	1.241206	1.526067	6.234693657	5.80191564	6.19833634	6.141029765	6.40473348	5.45312815	5.579472794	5.61487468	5.601109834	5.34644129
ENSMUSG00000(	Ppa1	5.43E-01	3.18E-01	7.67E-01	7.648284	5.08E+00	8.65E-05	1.55E-02	1.054965	1.45654	8.052201628	7.91261237	7.808565194	7.931285282	7.78554457	7.57249558	7.344347608	7.40777222	7.536153334	7.13185983
ENSMUSG00000(	Syde1	-8.47E-01	-1.20E+00	-4.96E-01	4.85594	-5.08E+00	8.65E-05	1.55E-02	1.373439	-1.79829	4.044558067	4.9649581	4.384801759	4.510483263	4.34668541	5.52714657	5.304307588	5.18682895	4.952703886	5.33692245
ENSMUSG00000(	Nipal2	-2.79E+00	-3.95E+00	-1.63E+00	0.826134	-5.07E+00	8.82E-05	1.56E-02	1.166254	-6.91653	-1.09688683	-1.01753358	0.792273826	-1.02929593	0.02809046	2.46058988	2.182239881	2.42626771	2.016790389	1.49880013
ENSMUSG00000(	Pde1b	-1.02E+00	-1.44E+00	-5.94E-01	4.800099	-5.06E+00	9.03E-05	1.58E-02	1.361265	-2.02344	3.638073624	5.0374389	3.877109604	4.291188749	4.52240759	5.27055957	5.204834932	5.38604874	5.677520397	5.09580582
ENSMUSG00000(	Lat	-5.77E-01	-8.18E-01	-3.37E-01	6.725237	-5.05E+00	9.16E-05	1.58E-02	1.065429	-1.49199	6.168858425	6.63105512	6.473421656	6.255736321	6.64800002	6.90642587	6.880215559	7.03344614	7.304564487	6.95064525
ENSMUSG00000(	Xlr4a	1.10E+00	6.43E-01	1.56E+00	3.815828	5.05E+00	9.25E-05	1.58E-02	1.434387	2.147676	4.446311149	3.85197126	3.835351297	4.510929772	5.20743001	3.35056399	2.984831894	3.04936288	3.420597619	3.50093271
ENSMUSG00000(	Krt80	1.31E+00	7.62E-01	1.86E+00	3.860251	5.04E+00	9.52E-05	1.61E-02	1.389551	2.47821	4.483839328	4.27883992	3.419639978	5.223664634	4.93081713	3.25493148	3.211388196	3.11151406	3.503869472	3.18400382
ENSMUSG00000(	Runx1t1	-1.74E+00	-2.47E+00	-1.01E+00	4.034415	-5.00E+00	1.02E-04	1.70E-02	1.407133	-3.33815	2.208921595	4.02185552	3.955573351	1.842547719	4.32426545	4.68592358	4.525671958	4.99777015	5.172934318	4.60868486
ENSMUSG00000(	Vipr2	-6.07E-01	-8.64E-01	-3.51E-01	5.517937	-4.98E+00	1.07E-04	1.77E-02	1.0469	-1.52361	5.349737008	5.20025106	4.939714813	5.255366256	5.18500975	5.8956681	5.695273522	5.78866149	5.994927274	5.87476492
ENSMUSG00000(	Smim24	-8.41E-01	-1.20E+00	-4.84E-01	5.314847	-4.95E+00	1.13E-04	1.84E-02	1.043663	-1.79143	4.356460022	5.08740208	5.281811465	4.797506755	4.7730795	5.86398747	5.514471232	5.76543854	6.188856841	5.5194596
ENSMUSG00000(	Cox5a	5.63E-01	3.23E-01	8.03E-01	7.398714	4.94E+00	1.16E-04	1.87E-02	0.768299	1.477134	7.817828272	7.32769043	7.71592539	7.868580982	7.39435886	7.1889458	7.167109146	7.00842459	7.428937918	7.06933491
ENSMUSG00000(	Gm10131	5.86E-01	3.36E-01	8.37E-01	7.117535	4.93E+00	1.20E-04	1.89E-02	0.748243	1.501187	7.578474342	7.08443759	7.677858253	7.538051767	7.00388288	7.02823963	6.748959463	6.85861195	6.822231838	6.83460589
ENSMUSG00000(	Ccnd1	6.22E-01	3.56E-01	8.88E-01	5.680145	4.93E+00	1.20E-04	1.89E-02	0.872942	1.538771	6.225782455	6.0697959	5.989138791	6.040131281	5.44861432	5.53869002	5.510858753	5.26969755	5.429955799	5.27878711
ENSMUSG00000(	Hdac7	-5.91E-01	-8.44E-01	-3.37E-01	5.66277	-4.91E+00	1.24E-04	1.90E-02	0.87907	-1.50582	5.321278121	5.30696846	5.238802932	5.374653435	5.61628615	5.8038849	5.978650532	6.05669231	6.119601194	5.81087751
ENSMUSG00000(	Tmem47	-3.15E+00	-4.50E+00	-1.80E+00	-0.03145	-4.91E+00	1.24E-04	1.90E-02	0.636639	-8.86328	-1.94488374	-0.6549635	-1.559711503	-0.3877499	-1.40931486	1.3212532	1.970476601	1.7473494	1.412410434	2.19067783
ENSMUSG00000(	Lgals1	9.73E-01	5.56E-01	1.39E+00	4.972068	4.91E+00	1.25E-04	1.90E-02	0.955335	1.963263	5.04076848	4.82974753	5.510338806	5.754684485	5.87106929	4.62388414	4.474318048	4.11985685	5.290872622	4.20513776
ENSMUSG00000(	Rexo2	5.20E-01	2.94E-01	7.45E-01	6.587546	4.86E+00	1.39E-04	2.06E-02	0.636256	1.433673	7.001340247	6.66916703	6.767663859	6.984836157	6.6605824	6.33224582	6.433790957	6.19472371	6.450392565	6.38071955
ENSMUSG00000(	Mturn	-1.16E+00	-1.67E+00	-6.56E-01	3.489556	-4.84E+00	1.44E-04	2.06E-02	1.097691	-2.23605	2.492521571	3.68131989	3.170156064	2.33247243	3.06371436	3.8841894	4.13086624	4.0529295	4.090115755	3.99727761
ENSMUSG00000(	Cux2	-3.04E+00	-4.36E+00	-1.73E+00	0.514915	-4.86E+00	1.38E-04	2.06E-02	0.639323	-8.25306	-0.89757803	-0.16953667	-1.415321593	-1.9727124	-0.16138734	2.65723689	1.801264218	1.48024533	1.746020334	2.08091426
ENSMUSG00000(	Glipr1	4.65E-01	2.63E-01	6.67E-01	6.712646	4.84E+00	1.43E-04	2.06E-02	0.592746	1.380434	6.926590317	6.93998309	7.004650619	7.05413021	6.8470747	6.43267564	6.533871104	6.57345502	6.264766627	6.54926576
ENSMUSG00000(	Il11ra1	-5.49E-01	-7.88E-01	-3.10E-01	7.4576	-4.85E+00	1.43E-04	2.06E-02	0.567673	-1.46304	7.133356037	7.41982259	6.97869188	7.01248586	7.43114838	7.73086014	7.779526976	7.69476752	7.929406955	7.46593053
ENSMUSG00000(	Airn	-3.05E+00	-4.38E+00	-1.73E+00	-0.3364	-4.84E+00	1.43E-04	2.06E-02	0.380551	-8.3012	-1.32821238	-2.239926	-0.678355999	-1.42522461	-4.40931486	1.49229874	1.471298804	1.39634407	1.388759166	0.96828541
ENSMUSG00000(	Slc25a5	6.13E-01	3.46E-01	8.80E-01	10.48378	4.83E+00	1.47E-04	2.07E-02	0.466501	1.529476	11.15351862	10.589547	10.38775396	10.97134779	10.4775055	10.117969	10.19356218	10.0883604	10.61999701	10.238264
ENSMUSG00000(	Alcam	-9.77E-01	-1.40E+00	-5.51E-01	4.316817	-4.83E+00	1.47E-04	2.07E-02	0.945465	-1.96899	3.467897784	3.63618161	4.168208952	3.856278435	4.34890836	4.92873872	4.579005373	4.77834026	4.256165557	5.14844959
ENSMUSG00000(	Gng11	-6.54E-01	-9.40E-01	-3.68E-01	5.524755	-4.81E+00	1.53E-04	2.11E-02	0.689033	-1.57328	4.871886608	5.29768069	5.276504994	5.256845944	5.46975779	6.08878948	5.872798087	5.77460647	5.603680338	5.73499835
ENSMUSG00000(	Rian	-3.76E+00	-5.40E+00	-2.11E+00	-0.12431	-4.81E+00	1.53E-04	2.11E-02	0.419414	-13.5022	-1.94488374	-2.68738498	0.300885441	-0.5438691	-5.99427736	2.19425412	2.627727963	1.76083263	1.00905474	2.03455863
ENSMUSG00000(	Rras	-1.23E+00	-1.77E+00	-6.92E-01	3.208327	-4.80E+00	1.57E-04	2.14E-02	1.045166	-2.3512	1.695120123	3.03060294	2.396932128	2.588942727	3.30721884	3.62286131	3.843908555	3.66906464	4.199516673	3.72909774

ENSMUSG00000(	Abcg3	-7.69E-01	-1.11E+00	-4.30E-01	5.623326	-4.78E+00	1.63E-04	2.20E-02	0.60968	-1.70444	5.04228551	5.87520437	5.451515753	5.197983065	4.51054163	6.27284361	5.985720848	5.98741284	5.833483176	6.07627365
ENSMUSG00000(	Mpped2	-2.59E+00	-3.74E+00	-1.44E+00	1.126428	-4.76E+00	1.71E-04	2.26E-02	0.796546	-6.02531	-0.89757803	1.2368877	1.203588239	-0.628758	-2.08738676	2.73502905	2.810850266	2.30265862	2.614554172	1.97443764
ENSMUSG00000(	Xlr4b	9.91E-01	5.52E-01	1.43E+00	3.985167	4.76E+00	1.72E-04	2.26E-02	0.795587	1.986991	4.637792682	4.21051567	4.001871896	4.521973979	5.06566191	3.14636988	3.856953491	3.33740539	3.895825134	3.1772988
ENSMUSG00000(	Rbm47	9.20E-01	5.13E-01	1.33E+00	3.906917	4.76E+00	1.73E-04	2.26E-02	0.78981	1.892302	4.265868765	3.98889269	4.531097366	4.638596196	4.67726371	3.65122983	3.316157954	3.37238934	2.759914776	3.86775853
ENSMUSG00000(	Obsl1	-9.92E-01	-1.43E+00	-5.52E-01	4.007275	-4.75E+00	1.75E-04	2.27E-02	0.834609	-1.98899	3.099510378	3.47706489	3.775218019	3.278132596	3.90809776	4.13436165	4.577134173	4.43154651	5.092862874	4.29881929
ENSMUSG00000(	Lancl3	-2.01E+00	-2.90E+00	-1.12E+00	1.620815	-4.74E+00	1.77E-04	2.28E-02	0.924867	-4.02137	1.067088901	1.43061223	1.501428004	0.701887313	-2.29383764	2.8927009	2.725575286	2.82885546	2.334082074	3.01976163
ENSMUSG00000(	Cd68	8.34E-01	4.61E-01	1.21E+00	4.393386	4.70E+00	1.94E-04	2.42E-02	0.620225	1.782754	4.612771414	4.87901507	4.753648929	4.624910449	4.86448074	4.29965367	3.723884958	3.65107569	4.70710589	3.81730951
ENSMUSG00000(	Plekhg1	-1.57E+00	-2.27E+00	-8.66E-01	1.781654	-4.71E+00	1.91E-04	2.42E-02	0.868608	-2.9608	0.591169159	1.44564044	0.668094415	1.068314868	1.06100508	2.6652076	2.591109371	2.37458355	2.900348481	2.45106751
ENSMUSG000001	Gm29946	6.49E-01	3.59E-01	9.40E-01	5.12424	4.71E+00	1.92E-04	2.42E-02	0.491534	1.568492	5.069322751	5.54449073	5.842066249	5.409662219	5.48398667	4.82405024	4.844686498	4.71740802	4.690274257	4.81645357
ENSMUSG00000(	Scrn1	-1.16E+00	-1.68E+00	-6.41E-01	3.563186	-4.70E+00	1.95E-04	2.42E-02	0.793922	-2.23856	2.292883906	3.67495739	2.781075619	2.885268594	3.11685831	4.075767	3.81879027	4.15602299	4.504694892	4.32553663
ENSMUSG00000(	1700003E16Rik	-3.74E+00	-5.42E+00	-2.06E+00	0.385796	-4.69E+00	2.01E-04	2.47E-02	0.382229	-13.377	-3.52984624	0.56742892	-3.000284094	1.338075137	-4.40931486	2.77976737	2.201802147	2.86126272	2.624714038	2.42435018
ENSMUSG000001	Gm37795	1.62E+00	8.90E-01	2.35E+00	1.989324	4.68E+00	2.02E-04	2.47E-02	0.84664	3.069421	3.052107509	2.79862724	2.680201018	2.251660385	3.42988893	0.60432536	1.518864487	1.60509681	1.746020334	0.20644515
ENSMUSG00000(	Cfp	9.97E-01	5.47E-01	1.45E+00	4.98064	4.67E+00	2.09E-04	2.53E-02	0.425281	1.996407	5.610216614	5.41685636	4.546610366	6.015535304	5.38672475	4.83292293	4.308283575	4.27120581	4.815301176	4.60273968
ENSMUSG00000(	A530040E14Rik	1.36E+00	7.45E-01	1.98E+00	3.028498	4.65E+00	2.15E-04	2.58E-02	0.704202	2.568714	3.175799968	3.94858043	3.182691903	4.470741242	3.2788655	2.7306299	1.837607341	2.53274768	2.55204497	2.57527422
ENSMUSG00000(	Enho	-3.52E+00	-5.11E+00	-1.92E+00	-0.98473	-4.64E+00	2.22E-04	2.63E-02	-0.2306	-11.4397	-1.45945691	-2.44637688	-5.807639016	-2.50322712	-1.35042117	0.70054068	0.857847746	0.61255262	0.73668247	0.81216621
ENSMUSG00000(	Ephb6	-1.12E+00	-1.62E+00	-6.09E-01	3.493103	-4.64E+00	2.23E-04	2.63E-02	0.671811	-2.16732	2.706646377	3.33498284	2.704113638	2.748912647	3.04189625	3.67115687	3.806065204	4.21353838	4.437122188	4.26659281
ENSMUSG00000(	Fgfr3	-1.50E+00	-2.18E+00	-8.17E-01	3.221567	-4.63E+00	2.29E-04	2.67E-02	0.688384	-2.82655	2.68350104	2.92196168	2.688216011	2.006327981	1.81950383	4.0332871	3.448962474	4.49340979	4.229784112	3.89071098
ENSMUSG00000(	Hnf4a	-9.70E-01	-1.42E+00	-5.24E-01	4.076401	-4.58E+00	2.52E-04	2.87E-02	0.434876	-1.9589	3.259361334	3.56226722	3.666066733	4.279589128	3.06911772	4.68983598	4.764738342	4.79153825	4.36319746	4.31829902
ENSMUSG00000(	Plxdc2	-7.66E-01	-1.12E+00	-4.14E-01	6.153148	-4.59E+00	2.49E-04	2.87E-02	0.122341	-1.70081	5.685353214	6.41852353	6.095112929	5.267161478	5.70399322	6.61747955	6.582040906	6.64085746	6.17941059	6.35854625
ENSMUSG00000(	Pafah2	5.52E-01	2.98E-01	8.05E-01	7.086236	4.58E+00	2.52E-04	2.87E-02	0.00552	1.465809	7.092022483	7.57417575	7.534296711	7.148023937	7.67737427	6.84152158	6.61615639	6.91746253	6.661880442	6.79944376
ENSMUSG00000(	Cav2	-1.37E+00	-2.00E+00	-7.36E-01	3.431511	-4.56E+00	2.64E-04	2.89E-02	0.500823	-2.57945	3.0700666	3.18185833	2.337019227	3.206431131	1.83227113	4.60121275	4.635840608	3.99420706	3.642323438	3.81388268
ENSMUSG00000(	Gfi1	2.12E+00	1.14E+00	3.10E+00	1.618052	4.56E+00	2.62E-04	2.89E-02	0.603372	4.343823	2.073038167	2.04794026	2.655885357	2.616866728	4.199248	1.70827633	-0.081779636	0.7808241	1.128107412	-0.947883
ENSMUSG00000(	Dock10	6.19E-01	3.34E-01	9.04E-01	5.134899	4.57E+00	2.56E-04	2.89E-02	0.194388	1.535507	5.608169512	5.57385519	5.696683432	5.377376787	5.08587395	4.78800352	4.843130192	4.98633714	4.360160206	5.02939692
ENSMUSG00000(	H2-Eb1	1.07E+00	5.75E-01	1.56E+00	5.437662	4.56E+00	2.62E-04	2.89E-02	0.140551	2.096125	5.403844413	6.37796996	5.850126019	6.007646916	5.80864284	5.68347294	4.60861914	4.51148898	5.622814505	4.50199783
ENSMUSG00000(	Gm20427	-1.63E+00	-2.39E+00	-8.79E-01	3.252033	-4.56E+00	2.62E-04	2.89E-02	0.553015	-3.10252	1.639918451	2.90303195	1.903718222	3.117294129	2.32729002	3.42253654	4.530379467	4.20419256	4.62860659	3.84335973
ENSMUSG00000(	Rab19	1.29E+00	6.95E-01	1.89E+00	2.756101	4.55E+00	2.66E-04	2.89E-02	0.54959	2.450716	3.184399276	3.42493605	3.710030372	2.877594667	3.49155195	2.25413901	1.388362463	2.40065706	3.066085685	1.76325661
ENSMUSG00000(	Rorc	-3.26E+00	-4.77E+00	-1.75E+00	1.574238	-4.55E+00	2.71E-04	2.92E-02	0.400976	-9.58374	-2.68184934	0.74800117	-0.598185651	0.054768336	3.27951824	3.58974204	2.932926863	3.29108924	2.989532182	2.13683968
ENSMUSG00000(	Timm23	4.75E-01	2.54E-01	6.96E-01	6.294308	4.53E+00	2.78E-04	2.94E-02	-0.03362	1.390027	6.620540805	6.34321194	6.544062184	6.504248731	6.50474133	5.98060297	5.934623153	6.1051723	6.345668314	6.06020942
ENSMUSG00000(	Higd1a	5.73E-01	3.07E-01	8.39E-01	6.007214	4.53E+00	2.78E-04	2.94E-02	-0.00562	1.487394	6.505612716	5.90032095	6.185072775	6.470256607	6.17067355	5.61942956	5.786181623	5.62113487	6.04877805	5.76467728
ENSMUSG00000(	Clca3a1	-1.11E+00	-1.63E+00	-5.95E-01	3.29895	-4.53E+00	2.79E-04	2.94E-02	0.479783	-2.16143	2.510169437	3.117626	1.848285197	2.661791512	3.82430482	3.8097754	3.757122548	3.76585653	3.820835111	3.87372847
ENSMUSG00000(	Tnfaip8l2	8.23E-01	4.40E-01	1.21E+00	4.066048	4.53E+00	2.82E-04	2.95E-02	0.290668	1.768868	4.047582586	4.52118194	4.350970672	4.758345485	4.60098012	3.43517747	3.62591894	3.51845133	3.78956805	4.01230582
ENSMUSG00000(	Cldn5	-4.41E+00	-6.46E+00	-2.35E+00	-0.74614	-4.52E+00	2.87E-04	2.98E-02	-0.35572	-21.2463	-1.7643115	-6.1468166	-1.900748421	-3.35122402	-2.29383764	2.33630755	0.906346264	0.55179859	3.005171306	1.19595277
ENSMUSG00000(	Aplp2	-4.91E-01	-7.21E-01	-2.62E-01	7.556527	-4.51E+00	2.94E-04	3.03E-02	-0.16679	-1.40589	7.208414942	7.69563197	7.396473572	7.212353611	6.95354219	7.8595439	7.754736413	7.86760553	7.897339731	7.71962919
ENSMUSG00000(	Adamtsl4	-2.45E+00	-3.61E+00	-1.30E+00	0.345664	-4.49E+00	3.09E-04	3.11E-02	0.136722	-5.47737	-1.09688683	-0.72055184	-0.450087012	-0.46369875	-2.29383764	1.64519764	1.127034379	1.98418333	1.800812807	1.92446918
ENSMUSG00000(	Trim47	-7.33E-01	-1.08E+00	-3.89E-01	4.984557	-4.49E+00	3.09E-04	3.11E-02	0.092865	-1.66252	3.985853597	4.54055909	4.840718566	4.65888443	5.15911	5.2913088	5.188994371	5.2764958	5.472007705	5.43163512
ENSMUSG00000(	Eng	-5.05E-01	-7.43E-01	-2.68E-01	8.033772	-4.48E+00	3.12E-04	3.11E-02	-0.24534	-1.41961	7.490439259	8.10311163	7.608498752	7.835756811	7.84954557	8.2527346	8.389495792	8.24408599	8.449410441	8.11464194
ENSMUSG00000(	Pnpla8	-6.14E-01	-9.02E-01	-3.25E-01	5.597886	-4.48E+00	3.12E-04	3.11E-02	-0.0397	-1.53009	5.329999102	5.25991989	5.332551687	5.293353333	5.61351528	6.12824777	6.025373111	5.89490071	5.256982254	5.84401507
ENSMUSG00000(	C130026I21Rik	1.55E+00	8.24E-01	2.29E+00	2.175795	4.48E+00	3.13E-04	3.11E-02	0.427904	2.937541	2.356606357	3.20553587	3.446302991	3.225177179	2.75411678	1.88698671	2.092658219	0.47239924	0.770019654	1.54814219
ENSMUSG00000(	Slc22a17	-7.73E-01	-1.14E+00	-4.08E-01	4.433897	-4.46E+00	3.25E-04	3.13E-02	0.132776	-1.70852	3.481381014	4.31568568	4.082625261	4.217112158	4.14143193	4.94846819	4.853989239	4.63377589	4.810858693	4.85364284
ENSMUSG00000(	Mecr	5.24E-01	2.76E-01	7.71E-01	5.407648	4.46E+00	3.26E-04	3.13E-02	-0.07745	1.437451	5.689806323	5.4655912	5.659457018	5.692623516	5.69441007	5.11630516	5.091426456	5.00487004	5.42772349	5.23427103
ENSMUSG00000(	Snrpa1	4.42E-01	2.34E-01	6.50E-01	7.441164	4.47E+00	3.18E-04	3.13E-02	-0.24831	1.358722	7.842039281	7.52638204	7.697552264	7.692760181	7.64811285	7.13669248	7.354488173	7.26633956	6.97645146	7.27081694
ENSMUSG00000(	Chchd4	4.90E-01	2.59E-01	7.22E-01	6.153445	4.46E+00	3.25E-04	3.13E-02	-0.1808	1.404687	6.620154851	6.38041603	6.239825757	6.428495331	6.40473348	5.99782678	5.973680614	6.01580628	5.716042704	5.75747069
ENSMUSG00000(	Cyp26b1	-2.60E+00	-3.83E+00	-1.38E+00	2.558265	-4.47E+00	3.21E-04	3.13E-02	0.422647	-6.07088	-1.60384682	2.06750253	1.857696901	1.842547719	2.16054075	4.45255257	3.615016911	3.17503111	4.111947622	3.90366456
ENSMUSG00000(	Nhp2	4.61E-01	2.43E-01	6.79E-01	7.117252	4.45E+00	3.34E-04	3.13E-02	-0.28008	1.376502	7.540945568	7.20260287	7.235900983	7.327728164	7.20186367	6.80591416	6.867257704	6.8096933	7.29704012	6.88357749
ENSMUSG00000(	Cfl1	4.09E-01	2.16E-01	6.03E-01	10.28743	4.46E+00	3.29E-04	3.13E-02	-0.3401	1.327912	10.65059972	10.3567657	10.35845461	10.54313184	10.4150781	10.0794289	10.10226485	9.97622905	10.26192259	10.130449
ENSMUSG00000(	Osm	9.39E-01	4.94E-01	1.38E+00	3.576109	4.45E+00	3.36E-04	3.13E-02	0.184403	1.916773	4.174749106	4.29301429	4.00774428	4.10984609	4.16937232	3.15856135	3.109016544	3.2196541	1.782778884	3.7363566
ENSMUSG00000(	Mfap2	-1.29E+00	-1.90E+00	-6.79E-01	3.565666	-4.45E+00	3.36E-04	3.13E-02	0.272171	-2.44461	1.5491051	3.58619873	2.846997012	3.368507033	3.00290212	4.06375698	4.285545594	4.15602299	4.806402489	3.99122222
ENSMUSG00000(	Igf2r	-8.45E-01	-1.25E+00	-4.44E-01	4.200407	-4.44E+00	3.40E-04	3.14E-02	0.129969	-1.79585	3.693190096	3.47706489	3.942230411	3.954381766	3.73873796	4.26072968	4.659024093	4.59666272	4.737853013	4.9441946
ENSMUSG00000(	Leng8	-5.67E-01	-8.35E-01	-2.98E-01	6.994698	-4.44E+00	3.38E-04	3.14E-02	-0.27363	-1.48108	6.611591658	7.27202163	6.695714689	6.40711247	6.73048374	7.22810681	7.291386467	7.22387105	7.125851438	7.36084355
ENSMUSG00000(	Psmc5	4.54E-01	2.38E-01	6.70E-01	7.321178	4.43E+00	3.47E-04	3.17E-02	-0.32797	1.369777	7.719622803	7.40019841	7.347653586	7.549189984	7.53478711	7.11102622	7.019011936	6.99509866	7.445922969	7.08926538
ENSMUSG00000(	Srgap3	-8.53E-01	-1.26E+00	-4.47E-01	4.481108	-4.42E+00	3.53E-04	3.19E-02	0.055955	-1.80586	4.068578519	4.0718435	4.322931547	3.677787063	4.18935802	4.79346828	5.141668236	4.53129293	5.130783199	4.88337316
ENSMUSG00000(	Psme2	4.42E-01	2.32E-01	6.52E-01	7.436634	4.43E+00	3.51E-04	3.19E-02	-0.34806	1.358278	7.91713777	7.56113132	7.534032686	7.726977497	7.46915761	7.3288596	7.267575199	7.21695109	7.202351695	7.1421611
ENSMUSG00000(	Evc2	-1.42E+00	-2.09E+00	-7.40E-01	2.651739	-4.41E+00	3.63E-04	3.26E-02	0.288322	-2.66817	2.465638277	2.77502434	2.006142175	1.810663658	0.1142471	3.23016014	3.563022273	3.71134444	3.637314075	3.2038292
ENSMUSG00000(	Exoc6b	-7.45E-01	-1.10E+00	-3.88E-01	5.35549	-4.39E+00	3.77E-04	3.36E-02	-0.16843	-1.67546	4.626995283	5.49749037	4.824447397	4.713788126	5.47077881	5.87347892	5.50859267	5.84037943	5.632916569	5.56603558
ENSMUSG00000(	Ggta1	-7.11E-01	-1.05E+00	-3.70E-01	4.718244	-4.39E+00	3.79E-04	3.36E-02	-0.07465	-1.63657	4.346670705	4.79202786	4.273178511	4.126129503	4.62319011	4.98076266	5.177925045	5.112645	4.548046737	5.20186412
ENSMUSG00000(	Tyrobp	1.13E+00	5.87E-01	1.67E+00	2.620175	4.38E+00	3.91E-04	3.38E-02	0.189352	2.189562	3.040009367	2.75706525	2.903167418	3.562862073	3.22730976	2.18928786	1.652818944	1.86438882	2.644821612	2.36001553
ENSMUSG00000(	Acsbg1	-3.99E+00	-5.91E+00	-2.07E+00	-1.24753	-4.38E+00	3.91E-04	3.38E-02	-0.57387	-15.9017	-4.26681184	-3.8248885	0.382185543	-3.35122402	-5.99427736	1.25941395	1.353805241	0.24145847	1.649805019	0.07520061
ENSMUSG00000(	Tubb4b	-5.43E-01	-8.05E-01	-2.82E-01	8.327725	-4.38E+00	3.86E-04	3.38E-02	-0.46667	-1.45748	8.374308154	7.9463616	7.677443097	8.066782455	7.94732051	8.47380824	8.650385009	8.56624147	9.129837207	8.44476126
ENSMUSG00000(	Insl6	9.08E-01	4.72E-01	1.34E+00	4.378471	4.38E+00	3.86E-04	3.38E-02	-0.07677	1.876318	4.506876864	4.34403428	5.297614764	5.062403905	5.08187017	4.39859183	3.71710377	3.68329603	3.554704317	4.13821041
ENSMUSG00000(	Tnfrsf25	-3.64E+00	-5.39E+00	-1.88E+00	0.266164	-4.37E+00	3.94E-04	3.39E-02	-0.29947	-12.4243	-5.85177434	-1.01753358	-0.192929172	-0.71895581	0.69222317	2.06224867	2.211484701	1.66369147	2.231447161	1.58173647
ENSMUSG00000(	Gapt	1.64E+00	8.41E-01	2.43E+00	1.006709	4.34E+00	4.25E-04	3.63E-02	0.151164	3.105884	1.799277355	1.46051372	1.626989212	2.511723224	1.58315147	0.35120643	0.396873962	-0.31493488	-0.10383932	0.75613217
ENSMUSG00000(	Fcgrt	-6.70E-01	-9.98E-01	-3.43E-01	6.105082	-4.31E+00	4.54E-04	3.83E-02	-0.46855	-1.59155	5.320027952	6.08410449	5.650254821	5.617442769	6.25276006	6.55985837	6.122871678	6.51820325	6.622497673	6.30279746
ENSMUSG00000(	Usp44	1.00E+00	5.12E-01	1.50E+00	2.716734	4.30E+00	4.68E-04	3.83E-02	0.004999	2.005513	3.534088064	3.26892517	3.308704945	3.016845853	2.61674744	2.47879448	2.211484701	2.02944806	2.703515606	1.9987875
ENSMUSG00000(	Itgb3	-9.46E-01	-1.41E+00	-4.83E-01	4.530964	-4.30E+00	4.65E-04	3.83E-02	-0.23234	-1.92678	4.278796226	4.56828741	3.852356876	3.979692854	3.27484932	4.48893451	5.207256577	5.10341383	5.370007992	5.18604638
ENSMUSG00000(	Plk2	-1.27E+00	-1.89E+00	-6.46E-01	4.750711	-4.30E+00	4.60E-04	3.83E-02	-0.21135	-2.405	2.990576007	4.69788917	3.752693818	3.78422876	6.03466469	5.53370282	5.26186118	5.34523583	5.281270623	4.82499025
ENSMUSG00000(	Slc6a13	7.71E-01	3.93E-01	1.15E+00	4.194307	4.30E+00	4.65E-04	3.83E-02	-0.22186	1.706973	4.289055434	4.61223934	4.33829313	4.933253094	4.25720505	3.87734386	3.536304935	3.8119214	4.390248784	3.89720231
ENSMUSG00000(	Med12l	-6.40E-01	-9.53E-01	-3.26E-01	4.766526	-4.30E+00	4.66E-04	3.83E-02	-0.28944	-1.55805	4.207732368	4.49246984	4.81942058	4.425880026	4.32976814	5.29076793	4.99115553	5.05503788	4.933031989	5.11999716
ENSMUSG00000(	Efcc1	-8.99E-01	-1.34E+00	-4.59E-01	3.655645	-4.30E+00	4.63E-04	3.83E-02	-0.07099	-1.86486	2.530979365	3.48777667	3.695908489	3.103544095	3.27016524	4.1886244	4.063710739	3.9976278	4.15478478	4.06332982
ENSMUSG00000(	Gsap	-6.58E-01	-9.82E-01	-3.35E-01	5.165719	-4.29E+00	4.72E-04	3.85E-02	-0.36682	-1.5783	4.598405911	5.11233817	4.630113056	4.770827424	5.15864122	5.38598655	5.495773012	5.68550705	5.617736823	5.20186412
ENSMUSG00000(	Spaca6	-1.05E+00	-1.56E+00	-5.32E-01	3.39895	-4.29E+00	4.79E-04	3.87E-02	-0.08088	-2.06425	2.419688691	3.0504001	3.239484896	3.384839604	2.45280587	4.15148583	4.267093607	3.99705824	3.254530775	3.77211296
ENSMUSG00000(	Cbx7	-8.81E-01	-1.31E+00	-4.48E-01	4.530982	-4.28E+00	4.84E-04	3.88E-02	-0.2527	-1.84191	3.485847565	4.43908485	4.351551167	3.586591145	4.97510916	4.97114999	4.844686498	4.9892039	4.980897395	4.68569821
ENSMUSG00000(	Ahnak	-1.60E+00	-2.39E+00	-8.12E-01	3.011248	-4.28E+00	4.83E-04	3.88E-02	0.002538	-3.029	1.424350069	2.50064183	2.818069827	2.061557501	2.67460763	3.7063463	4.361435682	3.82802307	3.729639193	3.00781377
ENSMUSG00000(	Ifi44	-6.40E-01	-9.56E-01	-3.25E-01	5.800633	-4.27E+00	4.90E-04	3.90E-02	-0.50948	-1.55887	5.136200188	5.96334042	5.569029046	5.297672783	5.5482709	6.29484521	5.897750982	6.15059151	5.945122707	6.20350187
ENSMUSG00000(	Apol7e	9.66E-01	4.90E-01	1.44E+00	4.415593	4.27E+00	4.93E-04	3.90E-02	-0.33983	1.953007	4.834848744	5.24598379	4.764606448	5.400119182	3.69861707	4.41286428	3.616584602	4.19244937	4.036292519	3.95356786
ENSMUSG00000(	Eif2s2	4.39E-01	2.22E-01	6.57E-01	6.968683	4.26E+00	5.07E-04	3.99E-02	-0.69281	1.355946	7.295818563	6.97556817	7.408197294	7.234174225	7.00053215	6.86683283	6.770800525	6.67629572	6.627577602	6.83103349
ENSMUSG00000(	Meis3	-2.17E+00	-3.25E+00	-1.09E+00	1.866059	-4.25E+00	5.18E-04	4.03E-02	-0.02549	-4.50405	0.5576166	1.12930781	-1.28407706	1.221665644	2.08787168	2.71212865	3.351617681	2.3657866	3.847106459	2.67156788
ENSMUSG00000(	Pbx1	-6.14E-01	-9.18E-01	-3.10E-01	6.720425	-4.25E+00	5.18E-04	4.03E-02	-0.67985	-1.53043	6.546167635	6.24308358	7.013138285	6.210636684	6.13982804	6.84240061	7.13707139	6.98148654	6.807796532	7.2826411
ENSMUSG00000(	Slc17a8	1.74E+00	8.76E-01	2.60E+00	2.404249	4.25E+00	5.22E-04	4.04E-02	-0.05553	3.3355	2.852129237	3.44750801	3.741182892	2.945233383	3.03638978	2.60017372	0.009850839	1.92552911	1.764516678	1.71997251
ENSMUSG00000(	Etfb	5.34E-01	2.68E-01	7.99E-01	8.193884	4.23E+00	5.38E-04	4.10E-02	-0.79896	1.447676	8.624831388	8.20612341	8.111131069	8.691349184	8.2618576	7.94243415	7.882410655	7.79710082	8.511625229	7.90997763
ENSMUSG00000(	Tspan3	-4.98E-01	-7.45E-01	-2.50E-01	7.438464	-4.23E+00	5.38E-04	4.10E-02	-0.76582	-1.41193	7.270891912	7.33157385	7.148192488	6.92466767	7.36835142	7.79358191	7.672998368	7.70732413	7.798991488	7.36806999
ENSMUSG00000(	Rbp1	-6.39E-01	-9.57E-01	-3.21E-01	6.199831	-4.23E+00	5.35E-04	4.10E-02	-0.64956	-1.55734	5.772564209	5.51896343	6.146193666	5.891473744	5.75684823	6.64947304	6.493046503	6.26914334	7.219974792	6.2806254
ENSMUSG00000(	B4galnt1	5.99E-01	3.00E-01	8.97E-01	5.770444	4.22E+00	5.47E-04	4.14E-02	-0.65167	1.514433	6.489800081	5.90326331	5.619684305	6.199499678	6.01379405	5.58977345	5.320560089	5.6044052	5.493081962	5.47057901
ENSMUSG00000(	Cep85	-5.94E-01	-8.92E-01	-2.97E-01	6.16999	-4.21E+00	5.64E-04	4.25E-02	-0.70897	-1.50977	6.165047877	6.05705137	5.712910916	5.708498976	5.99501069	6.22796892	6.677053016	6.6040546	6.036149799	6.51615808
ENSMUSG00000(	Uqcc2	5.35E-01	2.67E-01	8.02E-01	5.967472	4.21E+00	5.68E-04	4.26E-02	-0.70948	1.448548	6.248559482	5.76569855	6.252043431	6.331419496	6.46027899	5.60794828	5.713275298	5.52290945	6.02496432	5.74762595
ENSMUSG00000(	Hif3a	-1.31E+00	-1.97E+00	-6.54E-01	2.560372	-4.20E+00	5.82E-04	4.33E-02	-0.13123	-2.4859	2.108227595	2.31670778	1.449748827	1.095032206	2.71652908	2.9911046	3.279804659	3.07603486	3.70111711	2.86941016
ENSMUSG00000(	Fhdc1	-1.30E+00	-1.95E+00	-6.45E-01	2.579397	-4.19E+00	5.86E-04	4.34E-02	-0.14766	-2.45558	0.806437146	2.75102886	1.940553833	2.061557501	2.10901045	3.10096108	3.24722629	3.06520783	3.267557455	3.44443442
ENSMUSG00000(	Mrps10	4.27E-01	2.12E-01	6.42E-01	6.155192	4.19E+00	5.92E-04	4.36E-02	-0.77649	1.344344	6.347499249	6.37354672	6.450838687	6.353585706	6.48798608	5.93637478	6.066547741	5.95272576	5.694247378	5.88857056
ENSMUSG00000(	Hspe1	5.66E-01	2.81E-01	8.51E-01	6.972031	4.19E+00	5.94E-04	4.36E-02	-0.84997	1.480403	7.328927396	6.71611516	7.46722539	7.43461747	6.93548036	6.74315207	6.57364258	6.59702922	7.215477823	6.70864566
ENSMUSG00000(	Nrk	-1.97E+00	-2.96E+00	-9.76E-01	2.685865	-4.17E+00	6.11E-04	4.46E-02	-0.17501	-3.91844	2.342982518	2.00800151	2.473131754	0.108207595	2.79688553	3.31366671	3.907835882	4.15857192	2.604322249	3.1450446
ENSMUSG00000(	Mrto4	4.96E-01	2.45E-01	7.46E-01	5.849849	4.16E+00	6.28E-04	4.51E-02	-0.79441	1.409866	6.302702041	5.967212	5.982904977	5.980857293	6.28844622	5.6110464	5.655029263	5.6268803	5.745380287	5.33803224
ENSMUSG00000(	Rpl36al	5.77E-01	2.85E-01	8.69E-01	7.241638	4.16E+00	6.29E-04	4.51E-02	-0.91932	1.491984	7.350566855	7.20413639	7.831195077	7.573573637	7.29552253	7.07429365	6.819205915	6.72770516	7.68645083	6.85373463
ENSMUSG00000(	Lratd2	-7.12E-01	-1.07E+00	-3.51E-01	4.568268	-4.16E+00	6.28E-04	4.51E-02	-0.55316	-1.6376	4.195022273	4.41405118	4.611752396	4.11122099	4.01244143	5.22279356	5.027937148	4.95414228	4.258418518	4.87489648
ENSMUSG000001	Gm36963	-3.35E+00	-5.04E+00	-1.65E+00	-0.54436	-4.16E+00	6.31E-04	4.51E-02	-0.86442	-10.1646	-1.94488374	-3.33946167	-1.28407706	-4.08818962	1.45693375	0.76129471	1.167392116	1.00132446	0.619643049	0.20644515
ENSMUSG00000(	Dnah1	-1.05E+00	-1.59E+00	-5.19E-01	2.975449	-4.15E+00	6.36E-04	4.51E-02	-0.28397	-2.07576	2.456564694	2.74496711	1.967148043	2.765639734	2.11946481	3.6592337	3.213895219	3.74904171	3.367741385	3.71078919
ENSMUSG00000(	H2-Ab1	9.07E-01	4.47E-01	1.37E+00	6.834002	4.15E+00	6.38E-04	4.51E-02	-0.90886	1.874907	7.037919743	7.4835643	7.031761633	7.354235575	7.08903575	7.08675086	6.189912992	6.02770631	7.202699323	5.83643522
ENSMUSG00000(	Sfxn1	4.22E-01	2.07E-01	6.37E-01	7.452479	4.13E+00	6.66E-04	4.66E-02	-0.98798	1.339722	7.877315282	7.45808406	7.569978232	7.657344659	7.54621397	7.20935778	7.155685033	7.23347152	7.590943718	7.22639841
ENSMUSG00000(	Zmat3	1.33E+00	6.52E-01	2.01E+00	2.386162	4.14E+00	6.63E-04	4.66E-02	-0.28549	2.513313	2.79568409	3.67495739	3.047229367	2.937872678	2.36766642	0.87562738	2.340629852	1.51248566	2.664652783	1.64481515
ENSMUSG00000(	Acsl3	-8.83E-01	-1.33E+00	-4.33E-01	5.143414	-4.13E+00	6.68E-04	4.66E-02	-0.70078	-1.84479	4.823300584	5.14917039	5.135183349	4.082603411	4.78626241	5.70366894	5.869919743	5.64233335	5.072464899	5.16923556
ENSMUSG00000(	Acox2	-4.25E+00	-6.42E+00	-2.08E+00	-1.51235	-4.13E+00	6.73E-04	4.67E-02	-0.90668	-19.0396	-4.26681184	-6.1468166	-5.807639016	0.736238817	-4.40931486	1.64519764	1.085515109	0.38767921	0.659720488	0.99273845
ENSMUSG00000(	Lamb3	1.28E+00	6.25E-01	1.93E+00	2.060677	4.12E+00	6.84E-04	4.72E-02	-0.29299	2.425317	1.962006855	2.85599842	3.015728224	3.278132596	2.33215213	1.38055039	1.666963126	1.73373897	1.388759166	0.99273845
ENSMUSG00000(	Atp5e	7.19E-01	3.51E-01	1.09E+00	6.68493	4.11E+00	6.97E-04	4.76E-02	-0.98379	1.646243	6.835820053	6.2575267	7.353335359	7.136816928	7.3903715	6.44938276	6.223796253	5.98526064	7.026641455	6.19034751
ENSMUSG00000(	Rps27l	6.18E-01	3.02E-01	9.34E-01	6.522446	4.11E+00	6.95E-04	4.76E-02	-0.9707	1.534655	6.835382608	6.22686424	7.092276748	6.903096011	6.95973715	6.42494451	6.104801452	5.89947948	6.453245857	6.32463391
ENSMUSG00000(	Mamdc2	-6.24E-01	-9.43E-01	-3.04E-01	5.431689	-4.11E+00	7.00E-04	4.76E-02	-0.80043	-1.54087	5.014731876	5.1924767	5.119398153	4.79953872	5.74339251	5.77378966	5.642566472	5.88030463	5.731403238	5.41928512
ENSMUSG00000(	Ndufa12	4.86E-01	2.36E-01	7.36E-01	6.824863	4.10E+00	7.20E-04	4.82E-02	-1.02959	1.400669	6.966879734	6.60438851	7.220149515	7.147318066	7.32341789	6.64403355	6.487094755	6.52482715	6.859339055	6.47117738
ENSMUSG00000(	Epb41	-4.94E-01	-7.48E-01	-2.40E-01	6.458885	-4.10E+00	7.17E-04	4.82E-02	-0.98017	-1.40832	6.16538205	6.54120636	6.320677587	5.962819879	6.15260948	6.54966829	6.642781182	6.79584267	6.626295074	6.83157161
ENSMUSG00000(	Tacstd2	6.34E-01	3.08E-01	9.59E-01	5.523027	4.10E+00	7.17E-04	4.82E-02	-0.89048	1.551679	5.9632086	5.97504065	5.806610712	5.949357336	5.46156355	5.23886522	4.952545783	5.37403531	4.821939355	5.68710366
ENSMUSG00000(	Myl10	1.97E+00	9.56E-01	2.99E+00	6.802132	4.08E+00	7.42E-04	4.95E-02	-1.05409	3.929103	7.425948945	8.59628219	7.85647489	8.183954133	5.69790222	6.80906513	5.024343473	4.72774379	6.245917644	7.45368471
ENSMUSG00000(	Ltv1	4.83E-01	2.33E-01	7.32E-01	6.028331	4.08E+00	7.53E-04	4.98E-02	-1.00964	1.397529	6.442559223	6.30876749	6.512879508	6.326495618	5.83821713	5.84481516	5.897001141	5.90480309	5.323985109	5.8837818
ENSMUSG00000(	Rnf208	-1.83E+00	-2.77E+00	-8.84E-01	1.935177	-4.08E+00	7.54E-04	4.98E-02	-0.34706	-3.55326	1.067088901	0.36097804	-0.253050164	1.842547719	1.75391549	2.16700642	3.012622589	2.60135441	3.544037392	3.25526762

Table S2. Hallmark Oxidative Phosphorylation Top 71 Differentially Expressed Genes

Feature ID	External Gene Name	logFC	CI.L	CI.R	AveExpr	t	P.Value	adj.P.Value	B	linearFC	ko5	ko4	ko3	ko2	ko1	wt5	wt4	wt3	wt2	wt1
ENSMUSG00000030246	Ldhb	-0.37052	-0.61579	-0.12525	7.85463	-3.18190	0.00534	0.12315	-3.05250	-1.29282	7.30590	7.71913	7.75900	7.54122	8.01726	7.92208	7.92354	8.05241	8.43867	7.86715
ENSMUSG00000024900	Cpt1a	0.37102	0.06218	0.67986	6.90282	2.53040	0.02132	0.22350	-4.33749	1.29327	7.15613	7.39440	6.65358	7.18191	6.69025	6.70755	6.42757	6.73737	7.08161	6.99784
ENSMUSG00000025393	Atp5b	0.24155	0.01451	0.46860	9.73167	2.24093	0.03836	0.28266	-4.96814	1.18227	10.17148	9.79306	9.62010	9.96169	9.46224	9.70269	9.64784	9.55975	9.82177	9.57609
ENSMUSG00000063229	Ldha	0.25717	0.02194	0.49240	9.87571	2.30279	0.03390	0.26994	-4.85609	1.19514	10.21677	10.02113	9.72600	10.07805	9.61083	9.73960	9.66104	9.68614	10.22778	9.78980
ENSMUSG00000006057	Atp5g1	0.44074	0.17868	0.70280	7.01805	3.54243	0.00243	0.08760	-2.25037	1.35730	7.47693	7.00143	7.10264	7.31714	6.92166	6.94651	6.71368	6.61208	7.30353	6.78490
ENSMUSG00000004610	Etfb	0.53374	0.26808	0.79940	8.19388	4.23174	0.00054	0.04097	-0.79896	1.44768	8.62483	8.20612	8.11113	8.69135	8.26186	7.94243	7.88241	7.79710	8.51163	7.90998
ENSMUSG00000016319	Slc25a5	0.61304	0.34571	0.88036	10.48378	4.83026	0.00015	0.02066	0.46650	1.52948	11.15352	10.58955	10.38775	10.97135	10.47751	10.11797	10.19356	10.08836	10.62000	10.23826
ENSMUSG00000037916	Ndufv1	0.24642	0.01494	0.47791	7.52065	2.24224	0.03826	0.28236	-4.91587	1.18626	7.77131	7.64506	7.36713	7.73478	7.46298	7.45212	7.44371	7.34061	7.78575	7.20301
ENSMUSG00000009863	Sdhb	0.35789	0.10773	0.60804	7.68651	3.01339	0.00768	0.14313	-3.40600	1.28155	8.01695	7.67306	7.64215	7.97239	7.78267	7.69289	7.40388	7.50862	7.89760	7.27487
ENSMUSG00000022956	Atp5o	0.32688	0.06834	0.58542	8.02561	2.66309	0.01617	0.19786	-4.13014	1.25430	8.32992	7.83287	7.92771	8.41555	8.10696	7.90695	7.84489	7.74893	8.37232	7.77001
ENSMUSG00000028167	Bdh2	0.62641	0.26414	0.98867	5.15839	3.64208	0.00196	0.07786	-1.81451	1.54372	4.95960	5.52163	5.75603	5.74312	5.10310	5.12644	4.59574	4.72431	5.02160	5.03235
ENSMUSG00000027673	Ndufb5	0.27791	0.02171	0.53412	6.71324	2.28478	0.03514	0.27395	-4.79081	1.21244	6.90561	6.46570	6.83923	7.16536	6.77661	6.76993	6.56727	6.50110	6.51048	6.63110
ENSMUSG00000030647	Ndufc2	0.39030	0.07399	0.70661	6.61691	2.59901	0.01849	0.21073	-4.18685	1.31067	6.80844	6.33926	7.21871	7.12471	6.49130	6.75127	6.46043	6.29602	6.16295	6.51604
ENSMUSG00000021079	Timm9	0.28371	0.02573	0.54169	5.32296	2.31642	0.03298	0.26583	-4.55108	1.21732	5.46394	5.38017	5.47603	5.76095	5.13951	5.25612	5.21329	5.24566	5.05580	5.23811
ENSMUSG00000021794	Glud1	0.27973	0.02164	0.53783	5.35192	2.28288	0.03528	0.27446	-4.61463	1.21397	5.50082	5.29042	5.63994	5.68165	5.28359	5.19114	5.36307	5.21538	5.20883	5.14436
ENSMUSG00000032314	Etfa	0.21557	0.00182	0.42933	7.39259	2.12420	0.04829	0.30806	-5.12271	1.16117	7.62889	7.41483	7.20899	7.59232	7.71578	7.37373	7.23289	7.33161	7.15355	7.27331
ENSMUSG00000024099	Ndufv2	0.39426	0.16087	0.62765	7.33299	3.55813	0.00235	0.08652	-2.23523	1.31427	7.74756	7.19790	7.45284	7.61874	7.63951	7.21480	7.26869	7.02159	7.11127	7.05702
ENSMUSG00000024902	Mrpl11	0.30283	0.06043	0.54523	5.76234	2.63143	0.01728	0.20531	-4.01230	1.23356	5.99802	5.64801	5.75603	6.01291	6.18096	5.70103	5.59528	5.53767	5.60625	5.58727
ENSMUSG00000000563	Atp5pb	0.24593	0.03660	0.45526	8.51524	2.47453	0.02392	0.23329	-4.51033	1.18585	8.76310	8.43836	8.56695	8.77341	8.51140	8.48344	8.28848	8.49904	8.48786	8.34041
ENSMUSG00000004264	Phb2	0.27814	0.04723	0.50905	7.04929	2.53711	0.02102	0.22239	-4.33584	1.21263	7.37389	7.08901	6.98128	7.35043	7.04527	7.02731	7.05153	6.83461	6.95193	6.78770
ENSMUSG00000008892	Vdac3	0.23289	0.03336	0.43242	8.18519	2.45848	0.02472	0.23700	-4.53462	1.17519	8.50749	8.18047	8.13959	8.40380	8.22480	8.08445	8.15712	8.04751	8.04263	8.06402
ENSMUSG00000025968	Ndufs1	0.26670	0.03215	0.50126	6.74497	2.39502	0.02813	0.24926	-4.58840	1.20306	7.11786	7.00628	6.86833	6.85909	6.60042	6.51061	6.80425	6.71011	6.35934	6.61339
ENSMUSG00000032602	Slc25a20	0.32757	0.07768	0.57747	6.05133	2.76105	0.01316	0.18025	-3.80093	1.25490	6.35249	6.47592	6.05293	6.21445	6.00467	6.07987	5.92596	5.77627	5.62028	6.01044
ENSMUSG00000021771	Vdac2	0.24134	0.04767	0.43502	7.97799	2.62471	0.01752	0.20664	-4.20539	1.18209	8.28802	8.09490	8.03716	8.09617	7.91344	7.85520	7.90439	7.79427	7.84592	7.95045
ENSMUSG00000032279	Idh3a	0.31504	0.09176	0.53832	7.69302	2.97191	0.00840	0.14745	-3.49305	1.24405	8.24486	7.74183	7.68434	7.77122	7.84680	7.54090	7.53088	7.55434	7.40598	7.60906
ENSMUSG00000033845	Mrpl15	0.37063	0.14012	0.60115	6.50010	3.38662	0.00342	0.10379	-2.54540	1.29292	7.03449	6.46766	6.65256	6.60093	6.60540	6.28712	6.30687	6.28614	6.39952	6.36031
ENSMUSG00000063694	Cycs	0.70134	0.32263	1.08004	6.70043	3.90071	0.00111	0.06068	-1.46313	1.62601	7.49807	6.58624	7.19649	7.43070	6.69121	6.63361	6.69797	6.25042	5.48566	6.53390
ENSMUSG00000022427	Tomm22	0.37789	0.09838	0.65739	6.09856	2.84772	0.01095	0.16493	-3.63358	1.29944	6.51919	6.05194	6.12780	6.53721	6.09749	6.19170	5.75360	5.99528	5.74427	5.96713
ENSMUSG00000062580	Timm17a	0.29195	0.08539	0.49852	6.55172	2.97695	0.00831	0.14690	-3.41376	1.22430	6.80709	6.58001	6.59611	6.80029	6.72786	6.49928	6.39284	6.45630	6.27274	6.38470
ENSMUSG00000036880	Acaa2	0.32486	0.08750	0.56223	6.62749	2.88271	0.01017	0.15988	-3.60809	1.25255	6.72306	6.80720	6.66429	6.73013	6.77287	6.43663	6.24811	6.39229	6.99811	6.50226
ENSMUSG00000019179	Mdh2	0.37891	0.16482	0.59301	9.29364	3.72777	0.00162	0.07312	-1.91560	1.30036	9.59754	9.45272	9.20293	9.48581	9.47476	8.97822	9.02936	9.09898	9.52708	9.08904
ENSMUSG00000052738	Suclg1	0.33878	0.11249	0.56506	7.49142	3.15339	0.00568	0.12680	-3.10459	1.26468	7.73986	7.76785	7.32951	7.69522	7.51735	7.31002	7.24708	7.21859	7.73478	7.35398
ENSMUSG00000029198	Grpel1	0.35020	0.12490	0.57551	6.68786	3.27392	0.00437	0.11470	-2.80047	1.27474	6.93665	6.64983	6.68045	6.95825	6.95170	6.53832	6.35623	6.49065	6.72108	6.59548
ENSMUSG00000031633	Slc25a4	0.23081	0.02974	0.43187	8.87007	2.41788	0.02685	0.24475	-4.62385	1.17349	9.07781	8.91198	8.76026	8.98935	9.08408	8.81361	8.71864	8.65734	9.01745	8.67021
ENSMUSG00000042148	Cox10	0.42641	0.08396	0.76885	5.65155	2.62273	0.01760	0.20685	-4.00748	1.34388	6.14717	5.96595	5.61074	5.51234	6.19153	5.03406	5.40797	5.37623	5.49434	5.77520
ENSMUSG00000003873	Bax	0.42499	0.18257	0.66741	6.62074	3.69256	0.00175	0.07364	-1.89388	1.34256	6.84324	6.71214	6.54909	6.78500	7.30793	6.18194	6.40975	6.39553	6.66311	6.35972
ENSMUSG00000005510	Ndufs3	0.25537	0.03156	0.47919	6.68229	2.40332	0.02766	0.24737	-4.55951	1.19364	6.63891	6.78477	6.76849	6.72876	7.13855	6.50405	6.46680	6.59055	6.83019	6.37187
ENSMUSG00000017778	Cox7c	0.44397	0.05658	0.83135	6.90943	2.41396	0.02707	0.24531	-4.56014	1.36034	6.87166	6.42584	7.51102	7.40227	7.08711	6.95684	6.57977	6.28765	7.24886	6.72325
ENSMUSG00000050856	Atp5k	0.49318	0.06217	0.92418	5.45802	2.41012	0.02728	0.24600	-4.37799	1.40754	5.41910	4.84966	6.20812	5.72613	6.08754	5.35059	5.06497	4.80462	5.97223	5.09722
ENSMUSG00000016252	Atp5e	0.71918	0.35087	1.08748	6.68493	4.11288	0.00070	0.04762	-0.98379	1.64624	6.83582	6.25753	7.35334	7.13682	7.39037	6.44938	6.22380	5.98526	7.02664	6.19035
ENSMUSG00000022820	Ndufb4	0.61597	0.22123	1.01071	4.17526	3.28676	0.00425	0.11351	-2.36729	1.53259	4.43400	3.84629	4.84323	4.53618	4.51516	4.00891	3.75199	3.63297	4.42456	3.75934
ENSMUSG00000022820	Ndufb4	0.61597	0.22123	1.01071	4.17526	3.28676	0.00425	0.11351	-2.36729	1.53259	4.43400	3.84629	4.84323	4.53618	4.51516	4.00891	3.75199	3.63297	4.42456	3.75934
ENSMUSG00000022820	Ndufb4	0.61597	0.22123	1.01071	4.17526	3.28676	0.00425	0.11351	-2.36729	1.53259	4.43400	3.84629	4.84323	4.53618	4.51516	4.00891	3.75199	3.63297	4.42456	3.75934
ENSMUSG00000020219	Timm13	0.49243	0.17009	0.81477	6.78361	3.21776	0.00494	0.11931	-2.92300	1.40682	7.05643	6.59275	6.82876	7.11943	7.04447	6.39110	6.40085	6.26383	7.56612	6.57239
ENSMUSG00000033938	Ndufb7	0.39340	0.07546	0.71135	6.64091	2.60618	0.01821	0.20880	-4.16299	1.31349	6.93429	6.34077	6.73998	6.85701	6.89580	6.40961	6.39395	6.13708	7.38745	6.31315
ENSMUSG00000044894	Uqcrq	0.59116	0.23694	0.94537	6.45495	3.51525	0.00258	0.08940	-2.25916	1.50646	6.67154	6.17821	6.71959	6.90357	6.74487	6.21700	6.01573	5.77710	7.20058	6.12133
ENSMUSG00000059734	Ndufs8	0.27246	0.02257	0.52235	7.37583	2.29655	0.03432	0.27150	-4.80803	1.20787	7.48821	7.20177	7.39831	7.69057	7.45732	7.28146	7.19190	7.05959	7.79899	7.19018
ENSMUSG00000039016	Timm8b	0.39030	0.05328	0.72731	5.05653	2.43931	0.02570	0.24103	-4.24645	1.31066	5.20960	4.69789	5.28973	5.25240	5.56175	4.88845	4.73316	4.61534	5.59078	4.72622
ENSMUSG00000061518	Cox5b	0.32413	0.02236	0.62590	6.82175	2.26237	0.03675	0.27800	-4.83239	1.25191	6.98770	6.54017	6.93725	6.96497	7.19983	6.74166	6.54629	6.30662	7.38902	6.60402
ENSMUSG00000020163	Uqcr11	0.39785	0.02545	0.77025	6.70057	2.25022	0.03766	0.28036	-4.84312	1.31754	6.68374	6.29535	6.94579	7.03529	6.99671	6.58055	6.23036	6.20586	7.64560	6.38649
ENSMUSG00000059534	Uqcr10	0.48736	0.07621	0.89852	6.64755	2.49669	0.02285	0.22906	-4.37452	1.40188	6.67839	6.14637	6.93404	7.06135	7.05199	6.50373	6.15145	6.10374	7.68952	6.15490
ENSMUSG00000021606	Ndufs6	0.35962	0.01752	0.70173	5.45393	2.21414	0.04045	0.28882	-4.74203	1.28309	5.57082	5.08380	5.78806	5.68605	5.66884	5.42406	5.09011	4.98203	6.05157	5.19398
ENSMUSG00000038690	Atp5j2	0.46094	0.12370	0.79818	6.46826	2.87886	0.01025	0.15988	-3.59891	1.37644	6.55310	6.15497	6.84274	6.73332	6.85493	6.40783	6.05995	5.89643	7.06492	6.11438
ENSMUSG00000038717	Atp5l	0.41990	0.10091	0.73889	6.48569	2.77263	0.01284	0.17903	-3.81809	1.33783	6.54866	6.15224	6.90951	6.78046	6.75645	6.40921	6.14052	5.97229	6.97307	6.21449
ENSMUSG00000031231	Cox7b	0.43701	0.11742	0.75660	6.44660	2.88015	0.01022	0.15988	-3.59335	1.35379	6.45855	6.13714	6.89907	6.66084	6.85806	6.26696	6.05122	5.96429	7.03951	6.13031
ENSMUSG00000016427	Ndufa1	0.54850	0.16221	0.93479	5.25555	2.99075	0.00807	0.14586	-3.18950	1.46256	5.37524	4.82545	5.63789	5.64143	5.69354	4.96471	4.79081	4.74820	6.10697	4.77123
ENSMUSG00000036751	Cox6b1	0.45672	0.12002	0.79342	6.95996	2.85715	0.01073	0.16326	-3.68464	1.37242	7.00912	6.58726	7.26868	7.24030	7.44724	6.66942	6.58949	6.44857	7.73361	6.60596
ENSMUSG00000021520	Uqcrb	0.39620	0.14037	0.65203	7.49818	3.26195	0.00449	0.11544	-2.87742	1.31603	7.71535	7.22750	7.98331	7.87629	7.55237	7.47484	7.23121	7.23688	7.32186	7.36224
ENSMUSG00000014313	Cox6c	0.41026	0.09695	0.72357	7.05787	2.75808	0.01324	0.18029	-3.89880	1.32893	7.09768	6.69936	7.58165	7.59562	7.08372	7.10676	6.75909	6.73123	7.08121	6.84236
ENSMUSG00000026032	Ndufb3	0.31611	0.02236	0.60985	5.30861	2.26666	0.03644	0.27673	-4.62995	1.24497	5.40738	5.01746	5.73366	5.60936	5.43670	5.30413	5.04885	5.02875	5.31774	5.18206
ENSMUSG00000021764	Ndufs4	0.28228	0.01375	0.55081	6.18315	2.21413	0.04046	0.28882	-4.86203	1.21611	6.34636	5.87590	6.48555	6.46031	6.34251	6.22781	6.05458	5.91464	6.23269	5.89112
ENSMUSG00000037152	Ndufc1	0.48526	0.14212	0.82839	5.97680	2.97866	0.00828	0.14690	-3.34351	1.39983	6.05248	5.46927	6.46878	6.53965	6.43277	5.77563	5.74279	5.52390	5.98313	5.77960
ENSMUSG00000032330	Cox7a2	0.37203	0.04815	0.69591	6.22704	2.41945	0.02677	0.24475	-4.48363	1.29417	6.47774	5.84364	6.51232	6.60093	6.11718	6.05506	5.93622	5.81717	6.85980	6.05030
ENSMUSG00000062683	Atp5g2	0.30623	0.02739	0.58507	6.98217	2.31318	0.03320	0.26646	-4.75526	1.23647	7.09916	6.83235	7.13318	7.40833	6.71812	6.82866	6.75620	6.66240	7.45587	6.92741
ENSMUSG00000018770	Atp5g3	0.31057	0.03939	0.58175	7.18717	2.41223	0.02717	0.24531	-4.58354	1.24020	7.58194	6.92682	7.37584	7.54405	6.93072	7.21582	6.98024	6.92609	7.37246	7.01772
ENSMUSG00000025781	Atp5c1	0.29384	0.06906	0.51862	8.71606	2.75346	0.01337	0.18118	-3.96426	1.22590	9.00742	8.66200	8.69786	9.05886	8.65773	8.70520	8.50029	8.47303	8.73778	8.66046
ENSMUSG00000034566	Atp5h	0.28467	0.02383	0.54551	7.45406	2.29871	0.03418	0.27108	-4.80939	1.21813	7.66769	7.22596	7.47557	7.86982	7.41751	7.39050	7.24447	7.14655	7.66747	7.43504
ENSMUSG00000002416	Ndufb2	0.30137	0.00073	0.60201	5.41958	2.11143	0.04950	0.31191	-4.92747	1.23231	5.54852	5.18107	5.53696	5.79338	5.55111	5.39080	5.29864	4.98634	5.65019	5.25880
ENSMUSG00000026895	Ndufa8	0.27207	0.05117	0.49297	7.26052	2.59425	0.01867	0.21152	-4.23434	1.20754	7.47001	7.10511	7.38912	7.49285	7.34181	7.10884	7.18362	7.01035	7.45592	7.04757
ENSMUSG00000000088	Cox5a	0.56280	0.32298	0.80262	7.39871	4.94299	0.00012	0.01867	0.76830	1.47713	7.81783	7.32769	7.71593	7.86858	7.39436	7.18895	7.16711	7.00842	7.42894	7.06933
ENSMUSG00000030869	Ndufab1	0.34539	0.08397	0.60681	6.81155	2.78290	0.01257	0.17737	-3.83241	1.27050	7.07545	6.66002	6.80819	7.29325	6.88921	6.74545	6.76559	6.49540	6.81990	6.56301
